# Detecting alpha-synuclein aggregates with small molecules on single-molecule array†

**DOI:** 10.1039/d4sc07649d

**Published:** 2025-06-16

**Authors:** Jeff Y. L. Lam, Timothy S. Chisholm, Hadia Almahli, Elizabeth A. English, Zengjie Xia, Yunzhao Wu, Matthew R. Cheetham, Christopher A. Hunter, David Klenerman

**Affiliations:** a Yusuf Hamied Department of Chemistry, University of Cambridge Cambridge CB2 1EW UK mrc79@cam.ac.uk ch664@cam.ac.uk dk10012@cam.ac.uk; b UK Dementia Research Institute at University of Cambridge Cambridge CB2 0AH UK; c UK Dementia Research Institute at UCL London W1T 7NF UK; d Division of Life Science and State Key Laboratory of Molecular Neuroscience, The Hong Kong University of Science and Technology Hong Kong

## Abstract

Protein aggregates are promising biomarkers for early diagnosis of neurodegenerative disorders. Single-Molecule Array (SiMoA) is a powerful method to detect these aggregates at ultra-low concentrations in biofluids. Herein, we report a next-generation SiMoA assay using chemically synthesized small molecules, rather than antibodies, to capture alpha-synuclein aggregates, a protein hallmark in Parkinson's Disease and other synucleinopathies. These small molecule-based capturing agents contain aggregate-binding head groups, and a backbone functionalized with a primary amine for bead conjugation in the SiMoA assay. The most promising molecule, BF-79-2, captured recombinant alpha-synuclein aggregates, specifically excluding monomers, at picomolar concentrations. BF-79-2 also captured alpha-synuclein aggregates in human blood. Replacing antibodies with small molecules as capturing agents on the SiMoA platform enhances the assay versatility, since small molecules can be screened *in silico* and synthesized without laborious molecular biology techniques. The application of small molecules as capturing agents broadens the capabilities of the SiMoA platform, rendering it more adaptable for biomarker discovery and disease diagnostics.

## Abstract 2

A new assay leverages the small molecule BF-79-2, instead of antibodies, to specifically capture and detect α-synuclein aggregates at picomolar levels in biofluids, enhancing versatility for biomarker disease diagnostics.

## Graphical abstract

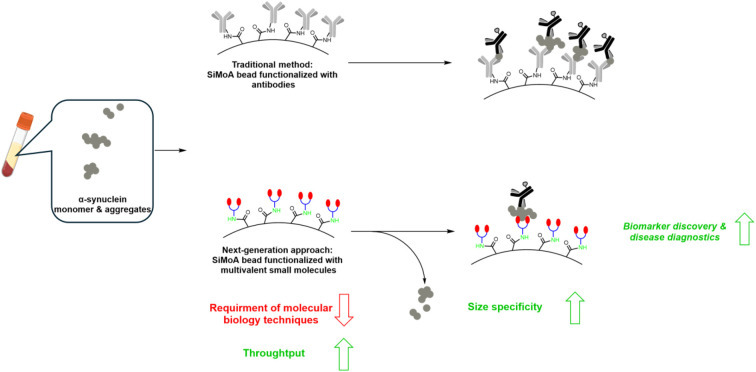

## Introduction

Parkinson's Disease (PD) is the leading cause of parkinsonism, with more than ten million people currently diagnosed with PD globally.^1^ The exact cause(s) of PD remains unknown. Yet, the aggregation of alpha-synuclein (α-synuclein) has been observed in PD patients' brains and is implicated in PD progression.^2^ Additionally, α-synuclein is the main component of Lewy bodies, which are large, insoluble, beta-sheet (β-sheet) rich formations formed in synucleinopathies, including PD and other Lewy body dementias.^3^ α-synuclein is a component of the presynaptic SNARE complex involved in neurotransmitter release.^4^ Large aggregates of α-synuclein are potentially toxic to neurons,^5^ and their accumulation can lead to deficits in neuronal signaling and function, ultimately resulting in symptom presentation. The mechanisms underlying the formation of these aggregates remain elusive. Nonetheless, detecting small, soluble protein aggregates that are at ultra-low concentrations and potentially cytotoxic,^2,6^ in biofluids such as serum could enable efficient diagnosis, given the accessibility of these samples. Hence, assays capable of specifically detecting α-synuclein aggregates with an ultra-high sensitivity are highly desirable.

One problem with detecting protein aggregates in biofluids, *e.g.*, serum, is the matrix effect, which refers to the steric hindrance to both the analytes and binding molecules in the crowded environment of many biofluids.^7^ To reduce the impact of the matrix effect, the biofluids used can be diluted or the protein aggregates present can be extracted from the matrix. However, dilution may further complicate the detection of low-concentration protein aggregates, while extraction can alter the disease-relevant morphology, *e.g.*, size and structure, of these aggregates. Other approaches to reduce the matrix effect include optimizing assay conditions, *e.g.*, pH and temperature, or introducing additives, such as surfactants. These methods, however, may not be suitable for immunoassays since they may affect the performance of the antibodies being used.

Unlike antibodies, synthetic small molecules that bind protein aggregates present a significant advantage,^8^ as they exhibit greater tolerance to the adverse assay conditions, thereby reducing matrix effects. In particular, thioflavin-T (ThT) is one of the most commonly employed fluorescent small molecules that binds to protein aggregates.^9^ We previously also demonstrated that a small molecule, CAP-1, which is based on the structure of Pittsburgh Compound B (PiB) used to detect beta-amyloid plaques with positron emission tomography,^10^ can be attached to magnetic beads to capture the cross β-sheets of amyloid with strong affinity.^11^ Also, an increasing number of radioligands are being reported to bind to α-synuclein fibrils, enabling the imaging of these aggregates in the brain.^12,13^ The main challenge with these small molecules is the lack of specificity, as they often bind to all β-sheet structures without discriminating between different types of protein fibrils such as beta-amyloid and α-synuclein. Distinguishing and quantifying different aggregates in PD and other synucleinopathies progression is clinically important, as the relative amounts of different aggregates may be informative on disease phenotype.

One strategy to improve both binding affinity and selectivity is the use of multivalent ligands.^11,14^ Linking two binding headgroups together can afford cooperative binding in the resultant multivalent ligand. Protein fibrils are an attractive target for multivalent ligands as the β-sheet structure present leads to a regular distribution of structurally similar binding sites. The binding of the first headgroup therefore brings the second headgroup in closer proximity to other nearby binding sites, increasing the effective molarity and the overall binding affinity. We have also demonstrated that matching the length of the linker joining the two headgroups to the distance between binding sites on a protein aggregate can increase the selectivity for that specific aggregate.^14^

There is a range of techniques available for detecting specific proteins within a sample. These include Enzyme-Linked Immunosorbent Assay (ELISA), western blotting, and mass spectrometry. ELISA involves the formation of an immunocomplex on a surface. The application of capture and detection antibodies in ‘sandwich ELISA’ allows for the detection of an antigen with high specificity.^15^ Sandwich ELISA assays are most commonly designed with capture and detection antibodies to different epitopes on a protein to detect total concentration of the target protein, including monomers and aggregates. In contrast, to specifically detect protein aggregates, the capture and detection monoclonal antibody could be the same, since a monomeric protein has only one epitope and thus alone would not be detected. However, when a sample contains a high concentration of monomers with a small number of aggregates, the capture antibodies may become saturated with monomers, hindering their ability to capture aggregates and thus reducing the sensitivity of the assay. This highlights the value of using an aggregate-specific capture agent. Western blotting and mass spectrometry are other frequently used techniques to identify specific proteins in a sample,^16,17^ but the sensitivities of these technique are insufficient to detect protein aggregates at picomolar levels in biofluids.^18^ Recently, a new platform has been developed called Single Molecule Array (SiMoA).^19^ SiMoA is based on the same principles of sandwich ELISA, but uses paramagnetic beads, instead of a flat surface, coated with capture antibodies to capture the analytes in the sample. The paramagnetic beads can then be pulled down by a magnet, enabling in the following steps the enrichment of analyte, formation of immunocomplexes, and effective and efficient rinsing that significantly reduces the background. These beads will be loaded into a microfluidic array and sealed in individual microwells, allowing the hydrolysis of a fluorogenic substrate in the well by an enzyme in the immunocomplex. The digital readout can then be obtained by calculating the ratio of fluorescent microwells over all loaded microwells to quantify the analyte concentration in the sample. Notably, SiMoA provides a femtomolar sensitivity, which is sufficient to detect specific protein aggregates at picomolar concentrations in biofluids. Despite its advantageous sensitivity and specificity, SiMoA still suffers from the aforementioned matrix effects, which reduce the performance in serum samples.

We herein present a next-generation approach using synthesized multivalent small molecules to specifically capture fibrillar α-synuclein aggregates on the SiMoA platform. This new assay offers optimized assay conditions to reduce the matrix effects, and allows selected protein aggregates in biofluids to be detected selectivity at ultra-high specificity.

## Results

### Selection of head groups targeting α-synuclein fibrils

**Fig. 1:**
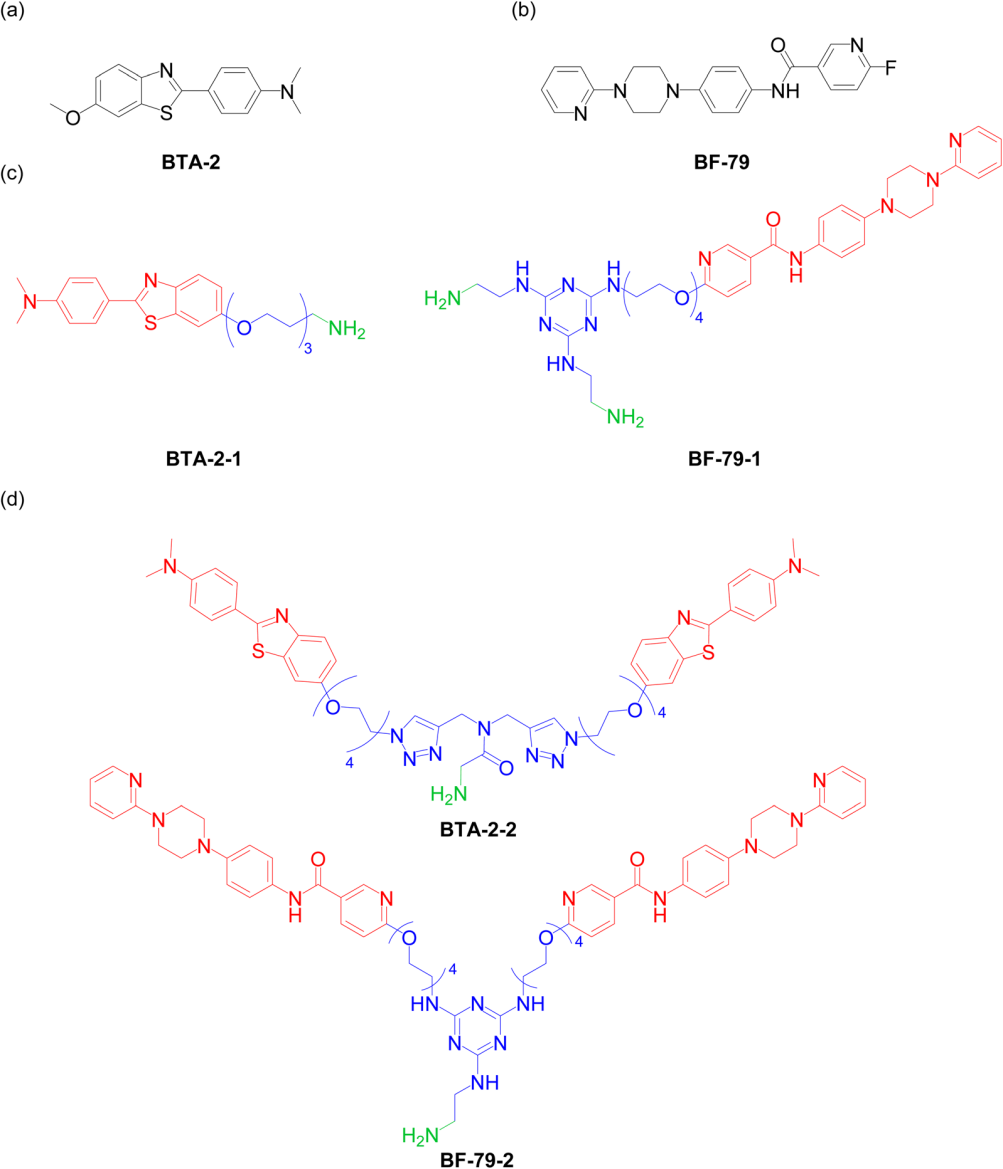
(a and b) Chemical structure of the head groups (a) BTA-2 and (b) BF-79. (c and d) Functionalization of head groups to (c) monovalent or (d) divalent small molecules. The head groups are labelled in red, while the linker and the amine moiety are labelled in blue and green respectively.

A head group that specifically targets α-synuclein fibrils was first required to develop multivalent ligands and the desired SiMoA methodology. The ligand BF-79 (Fig. 1b) was found to specifically target α-synuclein aggregates using unbiased screening approaches done by the Michael J. Fox Foundation's Alpha-synuclein Imaging Consortium. BF-79 specifically targets α-synuclein aggregates in Lewy body disease brain sections and had a measured dissociation constant (*K*_d_) of 4.77 nM against α-synuclein fibrils formed *in vitro*. Similar molecules reported in the literature also exhibit nanomolar binding to α-synuclein fibrils formed *in vitro* (*K*_d_ = 2.0–6.5 nM).^12,20–22^ Computational docking and photoaffinity labelling suggested that this ligand binds to a site encompassing the residues G86-F94-K96 on *in vitro* α-synuclein fibrils,^22^ although the binding sites present on *in vivo* fibrils are likely different. A structurally similar ligand was shown to label α-synuclein pathology in both human PD cortical tissue sections and A30P transgenic mice brain sections.^23^ These promising binding properties, combined with the ease of synthesis and derivatisation of this class of molecules, led us to select BF-79 to use in this study. Meanwhile, BTA-2 (Fig. 1a), a lipophilic and neutral derivative of Thioflavin-T, is a well-characterized compound that binds to amyloid structures with high affinity.^8,24^BTA-2 was therefore used as a benchmark for the new ligand BF-79 we tested in this study.

### Rational design of multivalent small molecules specific for α-synuclein fibrils

We then sought to design multivalent small molecules based on the selected BF-79 headgroup. The structures of the small-molecule ligands for capturing α-synuclein fibrils consist of either one (Fig. 1c) or two (Fig. 1d) head groups connected with PEG oligomers and attached to (a) pendant primary amine functional group(s) (see Schemes S1–S4† for full synthetic schemes).

To prepare the monovalent BF-79 ligand, the head group was first functionalized with a PEG_4_ chain. The terminal hydroxyl group was converted to an amino group *via* conversion to the azide, followed by reduction to afford the primary amine. The amino-derivatized BF-79 was then reacted with cyanuric chloride to afford a mono-substituted dichlorotriazine. Subsequent treatment with ethylene diamine under microwave irradiation afforded the monovalent small molecule BF-79-1. Cyanuric chloride was selected to assemble the core of BF-79 ligands due to the ability to precisely control the reactivity of this substrate. Each additional substitution of a chlorine atom on cyanuric chloride with an amine reduces the reactivity of the substrate to nucleophilic aromatic substitution. Only one chlorine is substituted at low temperatures, whereas the second substitution occurs at room temperature, and elevated temperatures are required for the final substitution. The degree of substitution can therefore be readily controlled using temperature, allowing for multivalent ligands to be selectively and iteratively assembled from a simple pool of amino-derivatized head groups.

Using this strategy, the divalent small molecule BF-79-2 was prepared by coupling two equivalents of amino-derivatized BF-79 with a single equivalent of cyanuric chloride. Meanwhile, BTA-2-2 was prepared using a copper-catalyzed azide-alkyne cycloaddition reaction between the azide-derivatized BTA-2 head group and dipropargylamine, followed by an amide coupling with *N*-Boc glycine and acidic deprotection. The divalent small molecules with two head groups mimic the Y-shaped structure of antibodies which contain two antigen-binding regions on a crystallizable fragment. It is noteworthy that the size of the divalent small molecules (∼1 kDa) is significantly smaller than that of an immunoglobulin G (IgG) antibody (∼150 kDa). This dimeric design can give rise to cooperative binding as when one head group binds to one binding site, the second head group is in closer proximity to other nearby binding sites. This increase in effective molarity leads to an increased binding affinity. To achieve optimal cooperative binding, the linker connecting the two head groups should possess sufficient length to bridge the two binding sites. We therefore chose a tetraethyleneglycol linker for these divalent small molecules based on our previous work.^14^

### Application of small molecules on the SiMoA platform

**Fig. 2:**
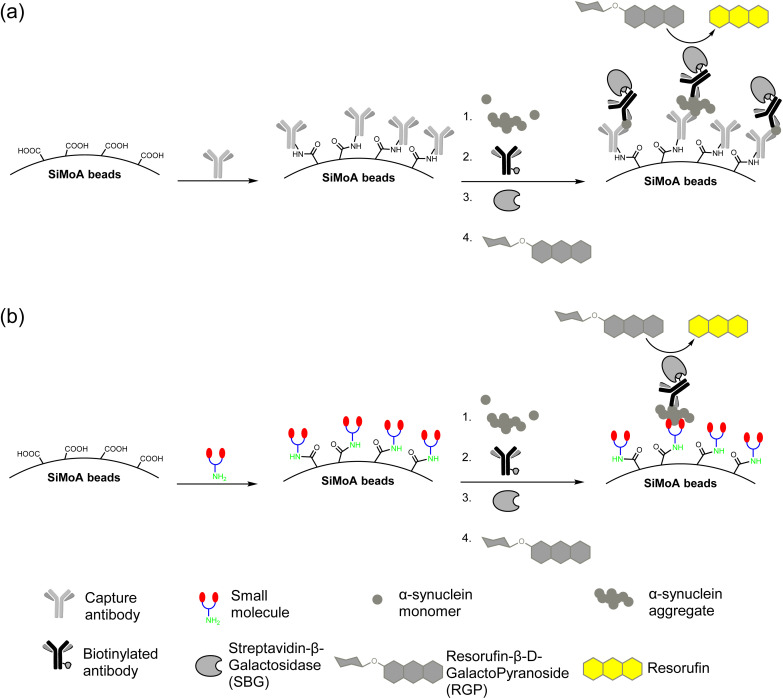
(a) Traditional SiMoA assay using antibodies as the capturing agent. (b) Next-generation SiMoA assay using small molecules as the capturing agent to selectively detect protein aggregates.

The carboxylic acid functionalized SiMoA beads allow amide coupling to lysine residues and the N-terminus of antibodies. Typical antibody-functionalized SiMoA beads thus have multiple paratopes per bead leading to cooperative binding and enhanced binding affinity. These antibody-functionalized SiMoA beads can subsequently capture the corresponding epitopes present in the sample, followed by the formation of the enzyme-labelled immunocomplexes to be detected on the SiMoA platform (Fig. 2a). Instead of using antibodies as the capture agent, we have used the aforementioned small molecules in this study, with the amine moiety allowing for covalent attachment to the carboxylated SiMoA beads. Upon interaction between the small molecule-functionalized SiMoA beads and the corresponding binding sites on the α-synuclein fibrils in the sample, the complex formed is similar to that with antibodies, which can be applied to the SiMoA platform (Fig. 2b). Since an antibody has multiple lysine residues and N-termini whilst the small molecules prepared have either one or two primary amine functional groups, the number of small molecules were used at 100-fold molar equivalent compared to the antibody to allow complete functionalization of the SiMoA beads for enhanced cooperative binding.

### Dimerized small molecules as the capturing agent can increase binding affinity

**Fig. 3:**
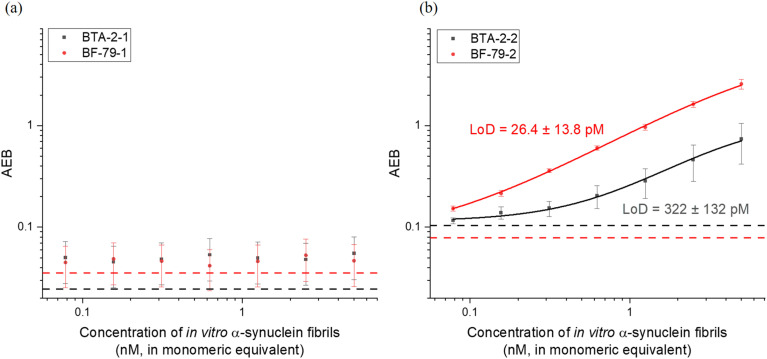
Measured average enzyme per bead (AEB) values of α-synuclein fibrils captured by (a) monovalent and (b) divalent small molecules conjugated on SiMoA beads and detected by anti-α-synuclein antibody, sc-12767. All graphs were fitted with a four-parameter logistic (4PL) curve. LoD represents the limit of detection, *i.e.* the concentration of α-synuclein fibrils (in monomeric equivalent) at 1.3 times background AEB level. The dotted lines represent the background AEB in the absence of α-synuclein fibrils for each assay. (a) *n* = 2 technical replicates. All data are expressed as mean ± mean deviation. (b) *n* = 4 technical replicates. All data are expressed as mean ± standard error of the mean.

We then tested the binding of the SiMoA-bead conjugated with monovalent and divalent small molecules to α-synuclein aggregates. No significant signals were observed using monovalent small molecules as capturing agents (Fig. 3a). However, when using divalent small molecules as capturing agents, the signal, average enzyme per bead (AEB) readout, increased with the concentration of α-synuclein fibrils (Fig. 3). Dimerization of the head groups therefore increased binding affinity. The monovalent head groups alone exhibited nanomolar binding affinity, whilst the divalent small molecules demonstrated a higher binding affinity in the picomolar range. Of the divalent molecules, BF-79-2 has a detection limit approximately 15 times lower than BTA-2-2, indicating a significantly higher binding affinity of BF-79-2 for α-synuclein fibrils. Therefore, BF-79-2 was selected as the best candidate for further studies.

### Using BF-79-2 as the capturing agent and the sc211 antibody as the detector on the SiMoA platform ensures selective and specific detection of α-synuclein aggregates

To evaluate the selectivity of BF-79-2 on the SiMoA platform, we tested BF-79-2 with α-synuclein fibrils and other amyloid-bearing structure, including amyloid-beta (Aβ) and tau fibrils, on the SiMoA platform. To examine the selectivity of BF-79-2, three different biotinylated detection antibodies were used: sc-12767 (or named as sc211, specific to α-synuclein), 6E10 (specific to Aβ), HT7 (specific to tau).

**Fig. 4:**
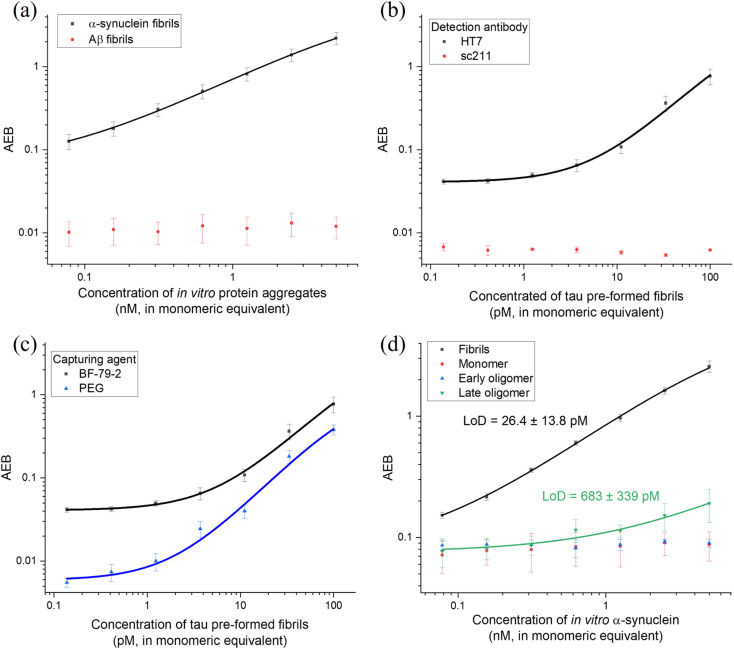
Validation of BF-79-2 for the detection of α-synuclein aggregates. (a) BF-79-2 was tested with α-synuclein fibrils and Aβ fibrils, with sc211 and 6E10 detection antibodies, respectively, demonstrating the ability of BF-79-2 to bind to α-synuclein fibrils but not Aβ fibrils. (b) BF-79-2 was tested with tau P301S mutant protein pre-formed fibrils using HT7 and sc211 detection antibodies, demonstrating its ability to bind to tau fibrils when detected with HT7 but not with sc211. (c) The tau fibrils were incubated with BF-79-2- or PEG-conjugated SiMoA beads. Detection using the HT7 antibody revealed increased signals in both conditions, suggesting that a portion of tau fibril binding to BF-79-2 may result from non-specific interactions. (d) BF-79-2 was tested with different sizes of α-synuclein aggregates, indicating the ability of the small molecule to bind to α-synuclein late oligomers and fibrils but not to monomers or early oligomers. All graphs were fitted with a four-parameter logistic (4PL) curve. LoD represents the limit of detection, *i.e.* the concentration of α-synuclein fibrils (in monomeric equivalent) at 1.3 times background AEB level. It is important to note that the LoD is based on the monomeric equivalent of α-synuclein and would correspond to a lower LoD based on concentrations of α-synuclein aggregates, *i.e.*, higher sensitivity, when considering that each aggregate is composed of a number of monomers. (b and c) *n* = 2 technical replicates. All data are expressed as mean ± mean deviation. (a and d) *n* = 3–4 technical replicates. All data are expressed as mean ± standard error of the mean.

When BF-79-2 was incubated with Aβ fibrils, negligible signal was detected using 6E10 detection antibody, whilst significant signal was demonstrated for the sc211 detection antibody when BF-79-2 was incubated with α-synuclein fibrils (Fig. 4a). This result demonstrates that BF-79-2 is highly selective for binding to α-synuclein aggregates rather than Aβ fibrils. On the other hand, when BF-79-2 was incubated with tau fibrils, an increased signal was observed with the HT7 detection antibody but not the sc211 detection antibody (Fig. 4b). This result indicates that BF-79-2 can bind to tau fibrils to some extent; however, these bound tau fibrils were not detected by the sc211 detection antibody.

Next, we evaluated the specificity of BF-79-2 on the SiMoA platform with different sizes of α-synuclein proteins generated through different aggregation timespans. In addition to the fibrils, α-synuclein monomers, early oligomers and late oligomers were tested with BF-79-2. The biotinylated sc211 detection antibody used is a sequence-specific antibody for α-synuclein and thus does not introduce any bias to the size specificity evaluation of BF-79-2. BF-79-2 exhibited negligible signal when interacting with α-synuclein monomers and early oligomers, but showed significant signal upon binding to α-synuclein late oligomers and fibrils (Fig. 4d). Compared to late oligomers, BF-79-2 exhibited a lower limit of detection (LoD) for fibrils, indicating a higher specificity towards large α-synuclein aggregates with more amyloid structures. These results indicate that this assay is highly specific to α-synuclein aggregate detection, as well as suggesting that BF-79-2 is biased towards binding larger aggregates.

### Assay optimization of BF-79-2 on the SiMoA platform

**Fig. 5:**
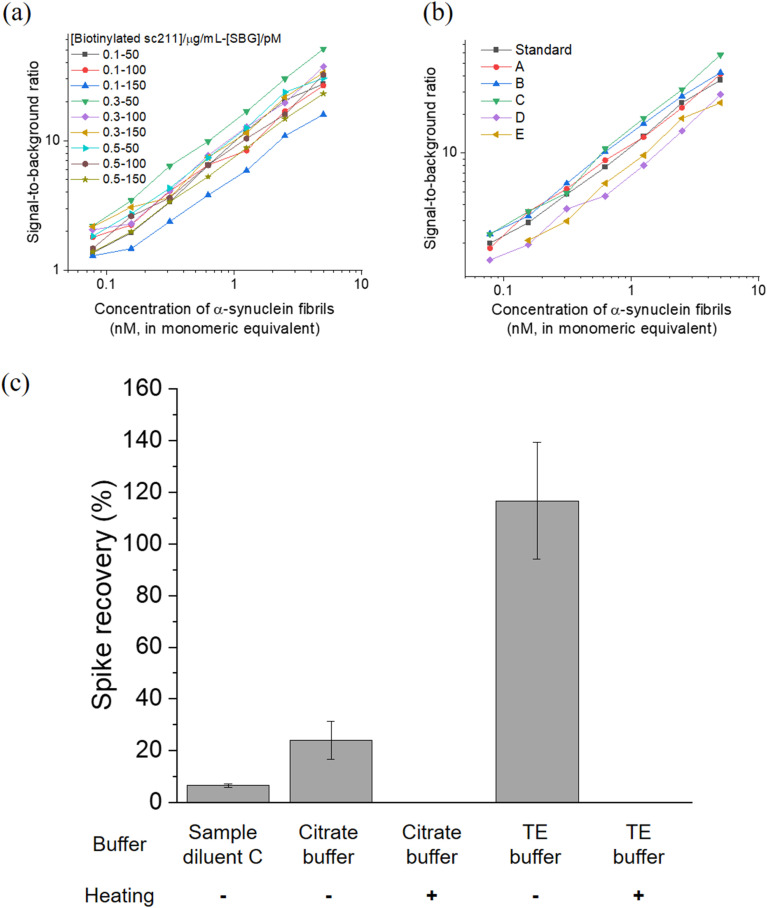
(a and b) Optimization of the BF-79-2 assay for *in vitro* α-synuclein aggregates under (a) combinations of different concentrations of biotinylated sc211 detector antibody and SBG and (b) commercially available sample diluents. (c) Recovery rate of the BF-79-2 assay for serum samples spiked with *in vitro* α-synuclein aggregates under different buffer and heating conditions. The symbol ‘−’ indicates the absence of heating while ‘+’ denotes the presence of heating at 95 °C at 800 rpm for 20 minutes. Spike recovery (%) was calculated as (observed concentration − endogenous concentration)/spiked concentration × 100%. All concentrations were back-calculated using a calibration curve fitted with four-parameter logistic (4PL) based on the average enzyme per bead (AEB) against different concentrations of *in vitro* α-synuclein aggregates in the absence of serum. Endogenous concentration refers to the concentration of α-synuclein aggregates naturally present in human serum samples, while the spiked concentration refers to the concentration of *in vitro* α-synuclein aggregates introduced, which in this case was 5 μM (in monomeric equivalent). *n* = 4 technical replicates. All data are expressed as mean ± standard deviation.

To enhance the performance of BF-79-2 on the SiMoA platform for the detection of α-synuclein aggregates, we first explored various combinations of concentrations of biotinylated detection antibody and streptavidin-β-galactosidase (SBG). The performance was evaluated based on the signal-to-background ratio at various concentrations of α-synuclein fibrils (Fig. 5a). Next, we investigated the performance of our assay by evaluating six commercially available sample diluents (Fig. 5b and Table S1†). The optimal conditions for *in vitro* aggregates were found to be 0.3 μg mL^−1^ detection antibody (biotinylated sc211), 50 pM SBG, and Diluent C (a phosphate buffer with low concentration of protein stabilizers, a heterophilic blocker and a surfactant). The LoD for BF-79-2 against *in vitro* α-synuclein fibrils under these conditions was measured to be 24 ± 8 pM (in monomeric equivalents). It is important to note that the LoD based on the monomeric equivalents of α-synuclein would correspond to a lower LoD based on the concentrations of α-synuclein aggregates, meaning higher sensitivity, when considering that each aggregate is composed of a number of monomers.

The optimized assay was then applied to measure α-synuclein aggregates in human blood-derived serum samples. Serum is a complex matrix that contains many other components, such as proteins, phospholipids, and salt, and as such we further optimized the assay to minimize the matrix effect in serum. Diluent C was selected as a benchmark, since it was selected as the most suitable option for measurement of *in vitro* α-synuclein fibrils with BF-79-2 capture and sc211 antibody detection (Fig. 5b) and was also used for α-synuclein aggregate quantity measurements in our previous antibody-based SiMoA work.^25^ Unlike antibodies, however, small molecules are more resilient to pH and temperature changes, providing more diverse options for suitable diluents. Citrate buffer (pH 6) and Tris–EDTA (TE) buffer (pH 9), which are commonly used in antigen retrieval for immunohistochemistry, were trialed in an attempt to minimize matrix effects. Furthermore, heating was also tested as another potential method to reduce matrix effects. Serum spiked with *in vitro* α-synuclein fibrils was used to assess the matrix effect in the three diluents (citrate buffer, TE buffer and Diluent C), with and without heating. The TE buffer showed almost full recovery (117% ± 23%) whereas the other buffers showed incomplete recovery. Meanwhile, an increase in temperature resulted in loss of signal suggesting that α-synuclein aggregates may denature during heating and lose their ability to bind to BF-79-2 (Fig. 5c). It was thus determined that TE buffer without heating provided the optimal conditions for detecting α-synuclein aggregates using BF-79-2 as the capturing agent on the SiMoA platform.

### BF-79-2 can detect human-derived α-synuclein aggregates in serum

**Fig. 6:**
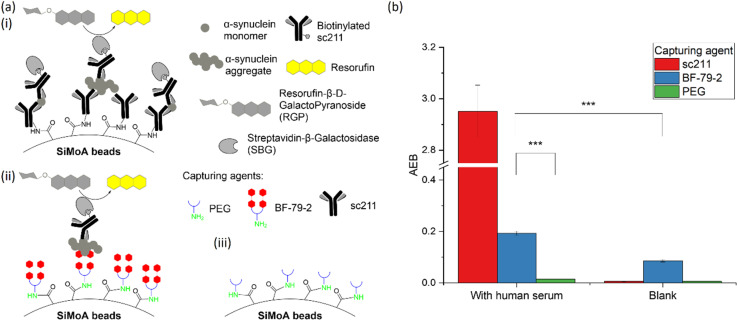
(a) Schematic diagrams showing SiMoA beads conjugated to (i) sc211, (ii) BF-79-2 and (iii) polyethylene glycol (PEG) to interact with α-synuclein present in the human serum samples and then detected by detector antibody, biotinylated sc211, which binds to SBG and subsequently triggers fluorescence through cleaving RGP. (b) BF-79-2 can specifically detect human-derived α-synuclein aggregates from serum. The serum samples were diluted four-fold in TE buffer and the blank is TE buffer only. *n* = 3–4 technical replicates. All data are expressed as mean ± standard error of the mean. Student's two-tail *t*-tests were performed to determine statistical significance. ***p < 0.001.

There is increasing interest in the potential for measurements of α-synuclein in the blood as an accessible method for early PD diagnosis and monitoring. However, there are key differences between *in vitro* α-synuclein aggregates and those in human biofluids that must be considered in assay development. α-Synuclein aggregates in biological systems form at different timescales to *in vitro* α-synuclein aggregates, with the possibility for aggregate breakdown through targeted removal pathways, such as proteostasis, and aggregate clearance mechanisms from the brain into biofluids. Also, α-synuclein aggregates in human biofluids can form different morphologies through post-translational modifications, including phosphorylation and truncation.^2,26^ Consequently, it was important to examine the suitability of BF-79-2 for capturing human-derived α-synuclein aggregates in serum samples. The serum sample, H6914, was commercially sourced from Merck. It is worth noting that, although the patient details for this serum sample are unknown, we have previously demonstrated that control serum contains α-synuclein aggregates, with only a slight increase in aggregate numbers when patients develop disease.^18,36^ Therefore, control serum with detectable α-synuclein aggregates can be used for initial experiments using our new assay. For positive and negative controls for α-synuclein aggregate capture, we evaluated SiMoA beads conjugated to sc211 (Fig. 6a(i)) and polyethylene glycol (PEG) (Fig. 6a(ii)). Upon the introduction of serum samples, at 25% concentration in TE buffer, both BF-79-2- and sc211-antibody -conjugated SiMoA beads captured α-synuclein aggregates present in the serum samples and yielded significantly higher readouts than the background signal (Fig. 6b). BF-79-2 gave a lower signal than the sc211 antibody-conjugated SiMoA beads, which can be largely explained by the size specificity of BF-79-2 to only large α-synuclein aggregates.

## Discussion

We have developed a next-generation single-molecule detection method on the SiMoA platform for α-synuclein aggregates using chemically synthesized small molecules. BF-79-2, the optimal small-molecule ligand, has two head groups attached to a triazine backbone with a pendant amine moiety available for functionalization, mimicking an IgG antibody despite its much smaller size in two orders of magnitude (∼1 kDa (small molecules) *cf.* ∼150 kDa (IgG)). Amyloid-binding ligands are promising alternatives to antibodies since they can readily be synthesized without laborious and expensive molecular biology techniques. In addition to identifying such small molecules by unbiased screening approaches, recent machine learning methods have shown success in the discovery of new amyloid-binding ligands.^27–29^ These ligands also impart selectivity towards aggregated protein fibrils over individual protein monomers and have the potential to target only specific fibril morphologies.^30,31^

Upon dimerizing the head groups as a small molecule, the resultant multivalency facilitates cooperative binding to the target fibril and thus increases both binding affinity and selectivity. The versatile triazine backbone allows multiple amino-derivatized head groups to be selectively and iteratively assembled, while the amine moiety allows covalent amide coupling to the SiMoA beads. Since the SiMoA beads are coated with a high density of carboxylic acid groups for functionalization, the cooperative binding of the small molecules on each bead may lead to an enhanced binding affinity. Due to their smaller size, a higher density of binding groups can be conjugated to SiMoA beads through multimerization, potentially resulting in a greater affinity. This cooperative binding also leads to a significantly reduced off-rate, allowing successive and more aggressive washing steps in assay preparation that reduce non-specific binding without any significant loss of analytes. Combined with the microfluidic-based digital counting in the SiMoA assay, the limit of detection using small molecules as the capturing agent can reach the picomolar range.

In this study, we presented the novel small molecule ligand BF-79-2, specifically designed for capturing α-synuclein aggregates. We demonstrated that BF-79-2 is specific to α-synuclein aggregates and does not bind to monomers. The LoD of BF-79-2 for *in vitro* α-synuclein fibrils is 24 pM ± 8 pM (in monomeric equivalents), which we note is approximately 360 times higher than that of the sc211 antibody targeting α-synuclein (Fig. S41†). This comparison, however, may not be accurate: sc211 shows no size specificity for α-synuclein aggregates, while BF-79-2 specifically binds to α-synuclein late oligomers and fibrils, which are a subset of α-synuclein aggregates captured by sc211, leading to comparatively low signals. It is important to note that the LoD is calculated based on the monomeric equivalents of α-synuclein, and would correspond to a lower LoD if it were based on the concentrations of α-synuclein aggregates. We therefore estimate that the binding affinity of BF-79-2 and sc211 antibody may be similar for a particular subset of α-synuclein aggregates, both capable of detecting within picomolar aggregate concentrations and potentially below.

Although BF-79-2 does not bind to Aβ fibrils, it can interact with tau fibrils in addition to its target, α-synuclein fibrils. However, almost 50% of the signal increase observed with tau fibrils was also detected when incubated with PEG-coated SiMoA beads lacking BF-79-2 (Fig. 4c). This result indicates that a significant proportion of the observed binding to tau fibrils is due to non-specific interactions rather than specific binding. Notably, this non-specific binding was not detected when using the sc211 detection antibody (Fig. 4b) and when incubating α-synuclein aggregates with PEG-coated SiMoA beads (Fig. 6b). These findings suggest that employing BF-79-2 as the capture agent in combination with the sc211 detection antibody on the SiMoA platform enables selective and specific detection of α-synuclein aggregates while minimizing interference from non-specific tau fibril binding.

The use of small molecules instead of antibodies as capturing agents on the SiMoA platform improves the versatility of the assay, in particular allowing for harsher conditions to be applied for antigen retrieval without the risk of denaturing the antibodies. This is a significant advantage for processing samples with significant matrix effects, *e.g.*, human serum. Here, we reported using TE buffer with a high pH to achieve almost full spike recovery of *in vitro* α-synuclein aggregates added to serum samples with BF-79-2 as the capturing agent and sc211 as the detection antibody. This was not possible with sc211, as the capturing agent because the harsh buffer conditions hindered proper antibody binding. We have also demonstrated that native α-synuclein aggregates in human serum samples can be captured by BF-79-2, and that these captured proteins can then be detected by the sc211 antibody on the SiMoA platform. Due to the adaptability and scalability of the SiMoA platform, our methodology could also be used for screening for new aggregate-binding molecules.

One limitation of our study is that the binding sites of the small molecule ligand BF-79-2 on α-synuclein aggregates are undefined. Most amyloid-binding ligands, such as BF-79-2, appear to bind within the grooves of β-sheet structures in α-synuclein aggregates. Previous molecular dynamics and docking studies have been applied to predict the interactions between various ligands and protein fibrils.^22,32–34^ While the binding site of an analogue of BF-79 has been proposed on α-synuclein fibrils formed *in vitro*,^22^ the morphology and heterogeneity of α-synuclein found in serum samples from PD patients is unknown. Additionally, dimeric ligands may have a binding mode distinct to monomeric ligands, and the binding properties of BF-79-2 could potentially change upon conjugation to SiMoA beads. Elucidating the morphology of α-synuclein fibrils found in serum, alongside carefully designed molecular dynamics simulation with experimental verification, would allow for the binding mode and plausible docking sites of these molecules to be more accurately determined.

Another limitation of this study is the lack of evaluation of diagnostic accuracy of the next-generation SiMoA assay utilizing BF-79-2 using serum samples from PD patients compared to healthy individuals. Given the small difference in α-synuclein aggregate levels between PD patients and controls,^18,36^ achieving high diagnostic accuracy will require large sample sizes to ensure sufficient statistical power and the measurement of additional aggregates, such as Aβ, to improve the diagnostic ratio.^25^ These aspects warrant investigation in our future studies.

In conclusion, we present a next-generation single-molecule detection method to selectively detect α-synuclein aggregates using the small molecule ligand BF-79-2. With aggregate capture on the SiMoA platform mediated by this small-molecule ligand, we lay the foundations for next-generation early diagnostics of PD and other synucleinopathies. We anticipate this technique will significantly enhance the capabilities of the SiMoA platform in targeting α-synuclein aggregates and other protein aggregates, facilitating biomarker discovery and advancing disease diagnostics for neurodegenerative disorders.

## Methods

### Synthesis of BTA-2-2

To S10 (Scheme S2,† 17.9 mg, 14.9 μmol, 1.0 equiv.) in CH_2_Cl_2_ (2.0 mL) was added 1.25 M HCl in MeOH (1 mL). The solution was stirred at rt for 16 h then concentrated under reduced pressure. The residue was dissolved in a minimal volume of methanol, and diethyl ether added dropwise to precipitate the product. The precipitate was recovered by filtration and dried *in vacuo* to afford BTA-2-2 as the hydrochloride salt (16.2 mg, 14.3 μmol, 96%).

^1^H NMR (400 MHz, DMSO-*d*_6_), *δ*: 8.17 (t, *J* = 5.7 Hz, 3H), 8.11 (s, 1H), 7.97 (s, 1H), 7.81 (dd, *J* = 11.4, 8.5 Hz, 6H), 7.63–7.57 (m, 2H), 7.05 (d, *J* = 8.2 Hz, 2H), 6.83 (d, *J* = 8.3 Hz, 4H), 4.57 (d, *J* = 17.4 Hz, 4H), 4.49 (dt, *J* = 9.7, 4.9 Hz, 6H), 4.18–4.11 (m, 6H), 3.79 (t, *J* = 5.0 Hz, 3H), 3.75 (t, *J* = 4.1 Hz, 3H), 3.59–3.48 (m, 16H), 3.00 (s, 12H). ^13^C NMR (101 MHz, DMSO-*d*_6_), *δ*: 165.4, 155.9, 148.1, 135.1, 128.1, 122.3, 115.5, 112.1, 106.6, 105.6, 69.9, 69.7, 69.6, 69.5, 69.5, 68.9, 68.6, 67.7. HRMS (ESI^+^): 1093.4772 *m*/*z*: calculated for C_54_H_69_N_12_O_9_S_2_^+^ = 1093.4752 [M + H]^+^.

### Synthesis of BF-79-1

A solution of S15 (Scheme S3,† 11.4 mg, 0.021 mmol, 1.0 equiv.) in CH_2_Cl_2_ (0.20 mL) was added dropwise to a solution of cyanuric chloride (3.9 mg, 0.021 mmol, 1.0 equiv.) in CH_2_Cl_2_ (0.20 mL) at 0 °C. DIEA (14.6 μL, 0.084 mmol, 4.0 equiv.) was added dropwise. The resultant solution was stirred at 0 °C for 2 h. Upon complete conversion to the mono-substituted triazine by LCMS, ethylene diamine (28 μL, 0.42 mmol, 20.0 equiv.) was added. The reaction was warmed to rt and stirred for 2 h, then heated to 50 °C under microwave irradiation for 50 min. Upon completion by LCMS, the solvent was removed *in vacuo* and the crude product was purified by RPFC (solvent A: H_2_O + 0.1% FA, solvent B: MeCN + 0.1% FA, 0 to 10% B) then normal phase flash chromatography (solvent A: CH_2_Cl_2_, solvent B: 7 N NH_3_ in MeOH, 0 to 100%) to afford BF-79-1 as an off-white solid (9.0 mg, 0.012 mmol, 57%).

^1^H NMR (400 MHz, DMSO-*d*_6_), *δ*: 10.18 (s, 1H), 8.78 (d, *J* = 2.5 Hz, 1H), 8.26 (dt, *J* = 8.7, 2.3 Hz, 1H), 8.13 (dd, *J* = 4.9, 2.0 Hz, 1H), 7.69–7.61 (m, 2H), 7.56 (ddd, *J* = 8.9, 7.0, 2.0 Hz, 1H), 7.01–6.97 (m, 2H), 6.94 (d, *J* = 8.7 Hz, 1H), 6.90 (d, *J* = 8.7 Hz, 1H), 6.66 (dd, *J* = 7.1, 4.9 Hz, 1H), 4.48–4.39 (m, 2H), 3.98 (s, 1H), 3.79–3.74 (m, 2H), 3.63 (t, *J* = 5.1 Hz, 4H), 3.59–3.56 (m, 2H), 3.54–3.50 (m, 6H), 3.20 (dd, *J* = 6.4, 3.9 Hz, 4H), 2.94 (s, 2H), 2.84 (dt, *J* = 11.0, 6.4 Hz, 2H). ^13^C NMR (176 MHz, DMSO-*d*_6_), *δ*: 170.0, 164.8, 163.1, 161.8, 159.0, 147.6, 147.5, 147.3, 138.7, 137.6, 131.2, 124.4, 121.5, 115.9, 115.9, 113.2, 110.2, 107.3, 69.9, 69.8, 69.8, 69.6, 68.7, 65.4, 63.6, 48.6, 44.6, 38.6, 38.3, 36.5, 35.1, 22.6. HRMS (ESI^+^): 768.4013 *m*/*z*: calculated for C_36_H_51_N_13_O_5_Na^+^ = 768.4029 [M + Na]^+^. 384.7062 *m*/*z*: calculated for C_36_H_52_N_13_O_5_Na^2+^ = 384.7051 [M + H + Na]^2+^.

### Synthesis of BF-79-2

A solution of S15 (Scheme S4,† 111 mg, 0.202 mmol, 2.7 equiv.), cyanuric chloride (13.7 mg, 0.074 mmol, 1.0 equiv.), and DIEA (52 μL, 0.30 mmol, 4.0 equiv.) in anhydrous THF (1.0 mL) and DMF (0.2 mL) was stirred at rt for 20 h. Upon complete reaction by LCMS, ethylene diamine (200 μL, 3.0 mmol, 40 equiv.) was added and the reaction was heated at 50 °C under microwave irradiation for 100 min. Upon completion by LCMS, the solvent was removed under a stream of nitrogen and the product was purified by RPFC (solvent A: H_2_O + 0.1% FA, solvent B: MeCN + 0.1% FA, 0 to 50% B). Purified fractions were lyophilised to afford BF-79-2 as a white solid (38.3 mg, 0.031 mmol, 42%).

^1^H NMR (700 MHz, 9 : 1 chloroform-*d*: methanol-*d*_4_), *δ*: 8.60 (s, 2H), 8.11 (d, *J* = 5.0 Hz, 2H), 8.05 (d, *J* = 8.7 Hz, 2H), 7.50 (d, *J* = 8.4 Hz, 4H), 7.48–7.45 (m, 2H), 6.92 (d, *J* = 8.6 Hz, 4H), 6.75 (d, *J* = 8.6 Hz, 2H), 6.67 (d, *J* = 8.6 Hz, 2H), 6.61 (t, *J* = 6.1 Hz, 2H), 4.44 (t, *J* = 4.4 Hz, 4H), 3.80 (t, *J* = 4.7 Hz, 4H), 3.71–3.32 (m, 36H), 3.31 (h, *J* = 1.5 Hz, 6H), 2.82 (s, 2H). ^13^C NMR (176 MHz, 9:1 chloroform-*d*: methanol-*d*_4_), *δ*: 165.4, 164.5, 159.5, 148.3, 147.7, 146.7, 138.4, 137.9, 131.0, 124.5, 122.1, 117.0, 113.8, 111.0, 107.8, 70.6, 70.6, 70.3, 70.0, 69.5, 65.8, 49.7, 49.6, 49.4, 49.3, 49.2, 49.1, 48.8, 45.4, 41.3, 40.3. HRMS (ESI^+^): 1236.6433 *m*/*z*: calculated for C_63_H_82_N_17_O_10_^2+^ = 1236.6431 [M + H]^+^.

### Preparation of *in vitro* α-synuclein aggregates

Wild type α-synuclein was expressed, purified in *E. coli* and stored at −80 °C as described previously.^35^ To remove pre-aggregation seeds, the solution was ultracentrifuged at 91 000 g at 4 °C for 1 hour (Optima TLX Ultracentrifuge, Beckman). The concentration of the supernatant was then determined by A_280_ (*ε*_280_ = 5960 M^−1^ cm^−1^). The supernatant was then diluted to 70 μM in 1×PBS supplemented with 0.01% NaN_3_ (Merck, Cat. No. 71290) and incubated at 37 °C with shaking at 200 rpm for 12 hours, 48 hours, and 96 hours as early oligomers, late oligomers and fibrils respectively. The aggregate was then aliquoted (10 μL), snap-frozen and stored at −80 °C until use.

### Preparation of Aβ42 fibrils

Lyophilized monomeric recombinant Aβ42 peptide (Stratech, Cat. No. A-1170-2-RPE-1.0 mg) was dissolved in PBS (pH = 7.4) at 200 μM on ice. The solution was immediately aliquoted and snap frozen. To prepare recombinant Aβ42 fibrils, an aliquot was thawed and diluted to 4 μM in 1×PBS supplemented with 0.01% NaN_3_ and incubated at 37 °C under quiescent conditions for one week. Thereafter, the aggregate was aliquoted (50 μL), snap-frozen and stored at −80 °C until use.

### Biotinylation of antibody

DBCO-PEG_4_-biotin (Merck, Cat. No. 760749, Lot No. MKCN1219) was dissolved in anhydrous DMSO at 10 mM as stock solution. It was then selectively conjugated on the carbohydrates of the Fc region of the monoclonal mouse anti-α-synuclein antibody (sc211) (Santa Cruz, Cat. No. sc-12767) *via* a SiteClick™ Antibody Azido Modification Kit (Invitrogen, Cat. No. S20026) according to the manufacturer's instructions. In brief, 200 μg of antibody was concentrated and buffer exchanged in the provided antibody preparation buffer by an Amicon spin filter (50 kDa MWCO, Merck, Cat. No. UFC505024). The antibody was then incubated overnight with β-galactosidase at 37 °C, followed by overnight coupling to UDP-GalNAz using β−1,4-galactosyltransferase (GalT) on the next day at 30 °C. The mixture was then purified by an Amicon spin filter (50 kDa MWCO). The concentration of the azido-modified antibody was calculated by A_280_. With the azido-modified antibody, 10 molar equivalents of DBCO-PEG_4_-biotin was introduced for copper-free strain-promoted click reaction. After overnight incubation at 37 °C, excess DBCO-PEG_4_-biotin was removed by an Zeba™ Spin Desalting Column (40 kDa MWCO, ThermoFisher, Cat. No. 87766). The biotinylated antibody was then concentrated by an Amicon spin filter (50 kDa MWCO), and its concentration was determined by A_280_. The labelling efficiency was determined using Pierce™ Fluorescence Biotin Quantification Kit (ThermoFisher, Cat. No. 46610).

### SiMoA bead functionalization

#### Conjugating SiMoA beads to antibodies

The SiMoA beads were conjugated to antibody, sc211 (Santa Cruz, Cat. No. sc12767), according to the manufacturer's instructions. Briefly, 4.2 × 10^8^ SiMoA 488-dyed singleplex beads (Quanterix, Cat. No. 104006, Lot No. 231817) were washed three times with 0.01 M NaOH, followed by three times with H_2_O. The beads were then resuspended in 280 μL of 25 mM cold MES buffer (pH 5). Meanwhile, EDC (ThermoFisher, Cat. No. A35391) and sulfo-NHS (ThermoFisher, Cat. No. A39269) were freshly dissolved in 25 mM cold MES buffer (pH 5) at 10 mg mL^−1^ and 40 mg mL^−1^, respectively. The washed beads were subsequently activated by mixing 4.5 μL of EDC and 15 μL of sulfo-NHS. The mixture was incubated on a HuLa mixer at 4 °C for 30 minutes. Meanwhile, ∼100 μg of antibody was buffer-exchanged to 25 mM cold MES buffer (pH 5) using an Amicon filter (50 kDa MWCO). After 30-min incubation, the activated beads were washed once with 25 mM cold MES buffer. Next, 45 μg of buffer-exchanged antibody was introduced to the activated beads. The mixture was incubated on a HuLa mixer at room temperature for 30 minutes. The conjugated beads were then washed twice with the provided bead wash buffer (in the Homebrew 2.0 Development Kit, Quanterix, Cat. No. 101354) and blocked by the provided bead blocking buffer (in the Homebrew 2.0 Development Kit) on a HuLa mixer at room temperature for 40 min. Finally, the blocked beads were washed once with the bead wash buffer and then once with the provided bead diluent (in the Homebrew 2.0 Development Kit). The beads were then resuspended in 300 μL of bead diluent and stored at 4 °C until use. The coupling efficiency, calculated using the A_280_ of the supernatants prior to the 40-min blocking step, suggested that >99% of the antibodies were successfully conjugated to the beads.

#### Conjugating small molecules to SiMoA beads

The SiMoA 488-dyed singleplex beads (4.2 × 10^8^ beads, Quanterix, Cat. No. 104006, Lot No. 231817) were washed three times with 0.01 M NaOH, followed by three times with H_2_O. The beads were then resuspended in 280 μL of 25 mM cold MES buffer (pH 5). Meanwhile, EDC (ThermoFisher, Cat. No. A35391) and sulfo-NHS (ThermoFisher, Cat. No. A39269) were freshly dissolved in 25 mM cold MES buffer (pH 5) at 10 mg mL^−1^ and 40 mg mL^−1^, respectively. The washed beads were subsequently activated by mixing 4.5 μL of EDC and 15 μL of sulfo-NHS. The mixture was incubated on a HuLa mixer at 4 °C for 30 minutes. Meanwhile, the small molecules, BTA-2-1, BTA-2-2, BF-79-1, BF-79-2, and methoxy-PEG_4_-NH_2_ (Merck, Cat. No. QBD10175-100 MG) were dissolved in DMSO to afford a 10 mM solution respectively. After 30-min incubation, the activated beads were washed once with 25 mM cold MES buffer. Next, 4 μL of each small molecule solution was introduced to the activated beads. The mixture was incubated on a HuLa mixer at room temperature for 30 minutes. The conjugated beads were then washed twice with the provided bead wash buffer (in the Homebrew 2.0 Development Kit, Quanterix, Cat. No. 101354) and blocked by the provided bead blocking buffer (in the Homebrew 2.0 Development Kit) on a HuLa mixer at room temperature for 40 min. Finally, the blocked beads were washed once with the bead wash buffer and then once with the provided bead diluent (in the Homebrew 2.0 Development Kit). The beads were then resuspended in 300 μL of bead diluent and stored at 4 °C until use.

### Sample preparation for SiMoA assays

#### *In vitro* samples

The *in vitro* α-synuclein fibrils were diluted in 156.25 pM, 39 pM, 9.77 pM, 2.44 pM, 0.61 pM, and 0.15 pM using Sample Diluent C (Quanterix, Cat. No. 101474) for assays using sc211as the capturing agent. Meanwhile, the *in vitro* α-synuclein aggregates were diluted in 5 nM, 2.5 nM, 1.25 nM, 0.625 nM, 0.3125 nM, 156.25 pM, and 78.13 pM using Homebrew Detector/Sample Diluent (Quanterix, Cat. No. 101359), Sample Diluent A, B, C, D, or E (Quanterix, Cat. No. 101477) for assays using small molecules as the capturing agent. The optimized buffer condition for assay using BF-79-2 to capture *in vitro* α-synuclein fibrils is Sample Diluent C.

The *in vitro* Aβ42 fibrils were diluted in 5 nM, 2.5 nM, 1.25 nM, 0.625 nM, 0.3125 nM, 156.25 pM, and 78.13 pM using Homebrew Detector/Sample Diluent. Furthermore, the tau P301S mutant pre-formed fibrils (Abcam, Cat. No. ab246003) were diluted in 100 pM, 33.3 pM, 11.1 pM, 3.70 pM, 1.23 pM, 0.412 pM, and 0.137 pM using Homebrew Detector/Sample Diluent.

#### Serum samples spiked with *in vitro* α-synuclein aggregates

*In vitro* α-synuclein fibrils were spiked into normal human serum (Thermo Fisher, Cat. No. 31876) to investigate the recovery rate of α-synuclein aggregates in the context of matrix effects. The diluted serum samples, *i.e.* 12.5 μL of normal human serum in 87.5 μL of Sample Diluent C (Quanterix, Cat. No. 101474), citrate buffer (20 mM, pH 6) or TE buffer (20 mM Tris and 2 mM EDTA, pH 9), were spiked with *in vitro* α-synuclein fibrils resulting in the final concentrations of *in vitro* α-synuclein fibrils in the human serum as 5 nM (in monomeric equivalent).

#### Serum samples without the addition of *in vitro* α-synuclein aggregates

Human serum (12.5 μL or 25 μL, Merck, Cat. No. H6914) was introduced into 87.5 μL or 75 μL of Sample Diluent C (Quanterix, Cat. No. 101474) for assays using sc211 or small molecules as the capturing agent, or into 87.5 μL or 75 μL of TE buffer for assays using small molecules as the capturing agent, resulting in a final prepared sample volume of 100 μL.

### SiMoA assay protocols

#### Assay using antibody as the capturing agent

SiMoA beads conjugated to sc211 were first washed three times with the provided bead diluent in the Homebrew 2.0 Development Kit (Quanterix, Cat. No. 101354). Meanwhile, 100 μL of prepared samples were loaded to each well on a conical bottom microplate (Quanterix). Then, 25 μL of the washed beads were introduced to each well to give the final-bead concentration as 2 × 10^7^ beads per mL. The samples and the beads were incubated on the plate shaker at 30 °C at 800 rpm for one hour. The plate was then washed by the SiMoA washer with the provided buffers. Afterwards, the biotinylated detector antibodies, *i.e.* biotinylated sc211, were then diluted to 0.3 μg mL^−1^ with the provided Homebrew sample/detector diluent and 100 μL of the diluted detectors were introduced to each well. The mixture was then incubated on the plate shaker at 30 °C with 800 rpm for 10 min. Similarly, the plate was then washed by the SiMoA washer. Meanwhile, the provided SBG concentrate was diluted to 50 pM with SBG diluent. To each well, 100 μL of the diluted SBG was introduced and the mixture was incubated on the plate shaker at 30 °C at 800 rpm for 10 min. Finally, the plate was washed by the SiMoA washer and loaded onto the SR-X™ Biomarker Detection System (Quanterix) together with the SiMoA discs, tips, and an equilibrated, well-shaken and opened RGP bottle.

#### Assay using small molecules as the capturing agent

SiMoA beads conjugated to small molecules were first washed three times with the provided bead diluent in the Homebrew 2.0 Development Kit (Quanterix, Cat. No. 101354). Meanwhile, 100 μL of prepared samples were loaded to each well on a conical bottom microplate (Quanterix). Then, 25 μL of the washed beads were introduced to each well to give the final-bead concentration as 2 × 10^7^ beads per mL. The samples and the beads were incubated on the plate shaker at 30 °C at 800 rpm for one hour (or at 95 °C at 800 rpm for 20 min to investigate if heating the beads and the human serum spiked with *in vitro* α-synuclein fibrils mixture can minimize the matrix effect). The plate was then washed by the SiMoA washer with the provided buffers. Afterwards, the biotinylated sc211 detector antibodies were diluted to 0.1, 0.3, and 0.5 μg mL^−1^ with the provided Homebrew sample/detector diluent. Alternatively, biotinylated 6E10 (BioLegend, Cat. No. 803007) or HT7 detector antibodies (Invitrogen, Cat. No. MN1000B) were diluted to 0.3 μg mL^−1^ with the provided Homebrew sample/detector diluent. Next, 100 μL of the diluted detectors were introduced to each well. The mixture was then incubated on the plate shaker at 30 °C with 800 rpm for 10 min. Similarly, the plate was then washed by the SiMoA washer. Meanwhile, the provided SBG concentrate was diluted to 50, 100, and 150 pM with SBG diluent for assays using sc211 detection antibodies or 150 pM with SBG diluent for assays using 6E10 and HT7 detection antibodies. To each well, 100 μL of the diluted SBG was introduced and the mixture was incubated on the plate shaker at 30 °C at 800 rpm for 10 min. Finally, the plate was washed by the SiMoA washer and loaded onto the SR-X™ Biomarker Detection System (Quanterix) together with the SiMoA discs, tips, and an equilibrated, well-shaken and opened RGP bottle. The optimized concentrations for the detector and SBG, using BF-79-2 as capture agent and biotinylated sc211 as detector, are 0.3 μg mL^−1^ and 50 pM, respectively.

### Data analysis

The readout of SiMoA assay is expressed as the average enzyme per bead (AEB). When the fraction of wells containing beads with enzyme activity (*f*_ON_) is below 0.7 (*i.e.* indicating that less than 70% of the beads are active), AEB is calculated in digital mode using the principles of Poisson distribution. In this case, AEB is calculated *via*:AEB_digital_ = −ln[1 − *f*_ON_].

For wells with *f*_ON_ > 0.7, the change in AEB with concentration is diminished because it becomes increasingly probable for the beads to have multiple bound enzymes. In this case, AEB is usually switched to analog mode, and calculated using:
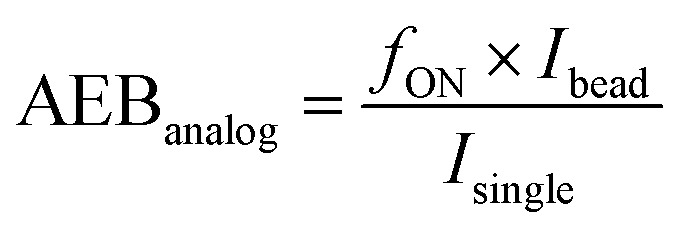


where
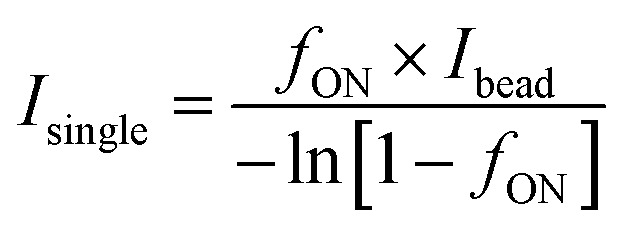
calculated from low concentration arrays with *f*_ON_ < 0.1. It should be noted however that because we are detecting aggregates, one single detection can give rise to multiple enzymes and thus analogue mode is not appropriate for aggregate detection. We thus simply avoid having cases where *f*_ON_ > 0.7 by diluting samples appropriately, and always using the digital mode calculation. After obtaining the AEB values at each calibration level, a four-parameter logistic (4PL) curve was fitted with the concentration of standard samples on the *x*-axis and AEB on the *y*-axis with blanks excluded. The data points are weighted by 1/*y*^2^ during the fitting process.
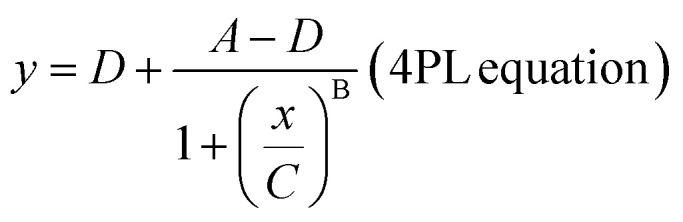


The limit of detection (LoD) for each assay was calculated using the parameter A, multiplied by a factor of 1.3, and then fitted back to the curve to determine the LoD in units of concentration. In this study, this concentration represents the monomeric equivalent concentration of the aggregates.

## Author contributions

J. Y. L. L. designed all experiments and developed the assays with input from all authors, with substantial contributions from M. R. C., E. A. E., and Z. X. J. Y. L. L., M. R. C., E. A. E., Z. X., and Y. W. conducted the SiMoA experiments and validated the experimental findings. T. C. and H. A. synthesized and characterized all small molecule capturing agents. J. Y. L. L., M. R. C., Z. X. and Y. W. functionalized the SiMoA beads with antibodies and small molecule capturing agents with input from T. C. and H. A. J. Y. L. L., M. R. C. and Z. X. analyzed the data, and all authors contributed to the interpretation of the study results. The initial draft of the paper was primarily written by J. Y. L. L. with valuable contributions from T. C., M. R. C., E. A. E. and Y. W., and all other authors provided critiques, feedback and editing to finalize the manuscript. C. A. H. and D. K. conceived and supervised the study. All authors read and approved the manuscript.

## Conflicts of interest

All authors declare that they have no conflicts of interest.

## Supplementary Material

SC-OLF-D4SC07649D-s001

## Data Availability

All data will be made available upon reasonable request following publication.
