# Supplementary material for: Detecting alpha-synuclein aggregates with small molecules on single-molecule array
Source: Chem Sci. 2025 Jun 16;16(29):13435–48. doi: 10.1039/d4sc07649d (PMC12199196; doi:10.1039/d4sc07649d)
Supplement: SC-016-D4SC07649D-s001 [file SC-016-D4SC07649D-s001.pdf]

Supplementary Information (SI) for Chemical Science.
This journal is © The Royal Society of Chemistry 2025

Supplementary Information

Detecting alpha-synuclein aggregates with small molecules on Single-Molecule Array

Jeff Y.L. Lam[1,2,4], Timothy S. Chisholm[1], Hadia Almahli[1], Elizabeth A. English[1,2], Zengjie Xia[1,2], Yunzhao Wu[1,2], Matthew R. Cheetham[1,2,3,*✉], Christopher A. Hunter[1,*✉], David Klenerman[1,2,*✉]

1 Yusuf Hamied Department of Chemistry, University of Cambridge, Cambridge, CB2 1EW, UK.
2 UK Dementia Research Institute at University of Cambridge, Cambridge, CB2 0AH, UK.
3 UK Dementia Research Institute at UCL, London, W1T 7NF, UK.
4 Division of Life Science and State Key Laboratory of Molecular Neuroscience, The Hong Kong University of Science and Technology, Hong Kong
* Co-corresponding authors

Contents

Materials and Instrumentation 2
Synthetic Schemes 3
Synthesis of BTA-2-1 5
Synthesis of BTA-2-2 8
Synthesis of BF-79-1 14
Synthesis of BF-79-2 20
NMR Spectra 21
Figure S41 and Table S1 41

Materials and Instrumentation

All solvents and chemicals were obtained from commercial sources and used without further purification unless otherwise stated. Thin-layer chromatography (TLC) analyses were performed on Merck TLC Silica gel 60 F254 glass plates (0.2 mm) or Merck TLC Aluminium Oxide 60 F254 basic glass plates (0.2 mm). Liquid chromatography-mass spectroscopy (LCMS) analyses of samples were performed using a Waters Acquity H-class UPLC coupled with a single quadrupole Waters SQD2. An Acquity UPLC CSH C18 Column, 130Å, 1.7 µm, 2.1 mm x 50 mm was used as the UPLC column.

Purification of compounds by silica column chromatography were performed using an automated system (Combiflash® Rf+ or Combiflash® Rf+ Lumen) with prepackaged silica cartridges (25 µm or 50 µm PuriFlash® columns) unless otherwise specified. Purification of compounds by reverse phase flash column chromatography (RPFC) were performed using an automated system (Combiflash® Rf+ or Combiflash® Rf+ Lumen) with prepackaged C18 cartridges (Interchim *puriFlash*® C18-HP flash column, 15 µm pore size) unless otherwise specified.

^{1}H and ^{13}C Nuclear Magnetic Resonance (NMR) spectra were recorded using a Bruker 700 MHz Avance II+ TXO Cryoprobe, a Bruker 600 MHz Avance 600 BBI spectrometer, a 500 MHZ Avance III Smart Probe spectrometer, or a 400 MHz Avance III HD Smart Probe spectrometer at 290.0 ± 0.1 K. Residual solvent peaks were used as an internal standard for calibration. All chemical shifts are quoted in ppm on the δ scale and the coupling constants are expressed in Hz. Signal splitting patterns are described as a singlet (s), broad singlet (br s), doublet (d), triplet (t), quartet (q), or multiplet (m). Fourier Transform Infrared Spectroscopy (FT-IR) spectra were collected with an ALPHA FT-IR Spectrometer from Bruker. Melting points (MP) were recorded with a Mettler Toledo MP90 melting point apparatus.

Synthetic Schemes

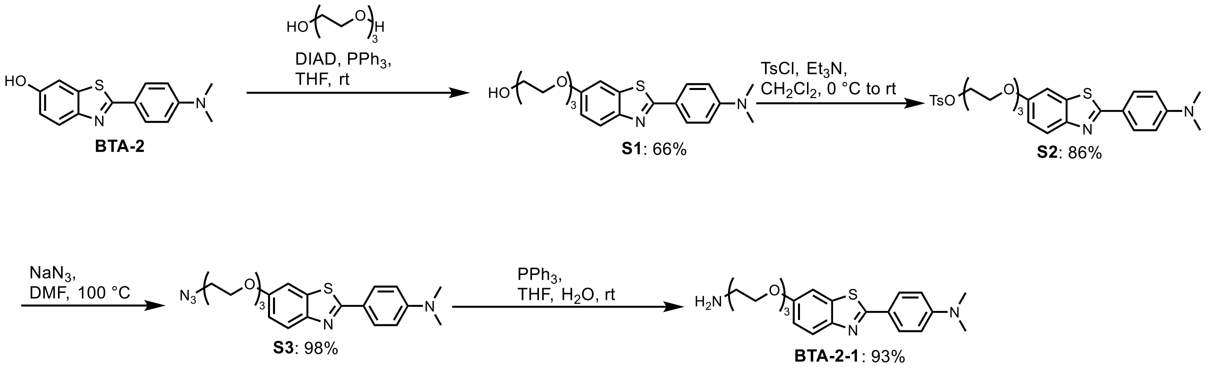

Scheme S1. Synthetic scheme for **BTA-2-1**.

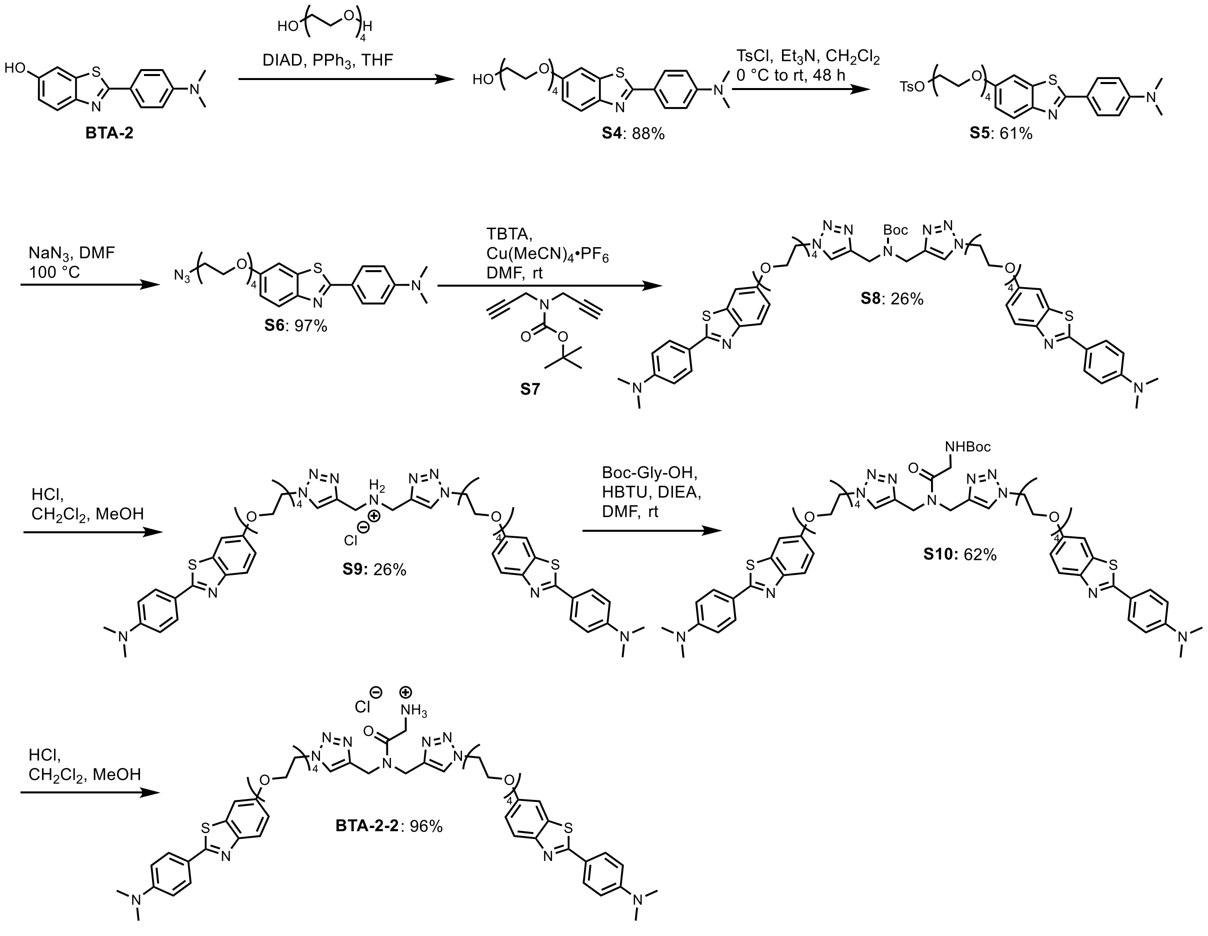

Scheme S2. Synthetic scheme for **BTA-2-2**.

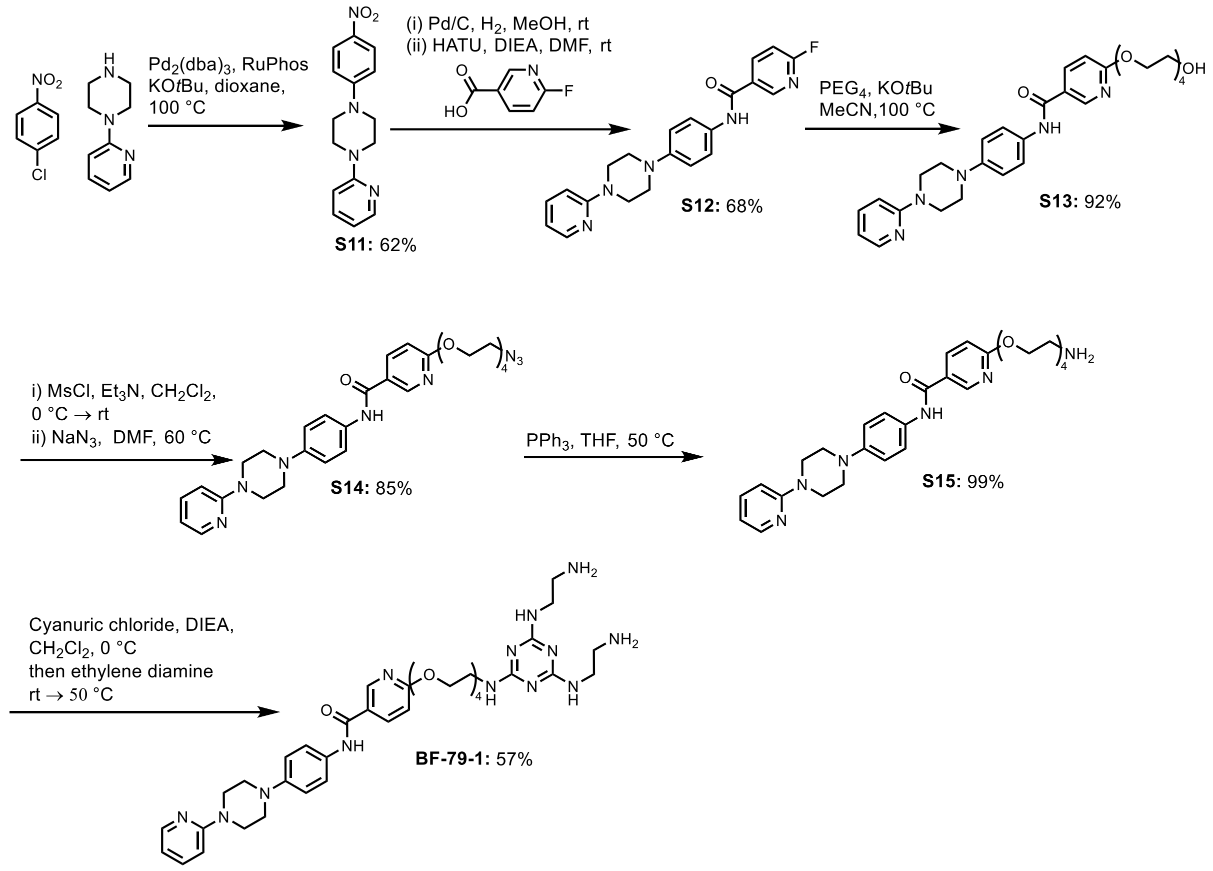

Scheme S3. Synthetic scheme for **BF-79-1**.

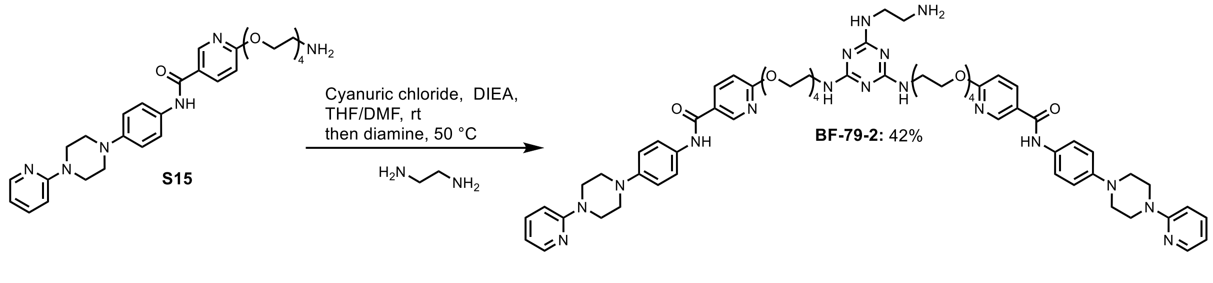

Scheme S4. Synthetic scheme for **BF-79-2**.

Synthesis of BTA-2-1

The synthetic route used to access **BTA-2-1** is summarized in Scheme S1.

*2-(4-(dimethylamino)phenyl)benzo[d]thiazol-6-ol (**BTA-2**)*

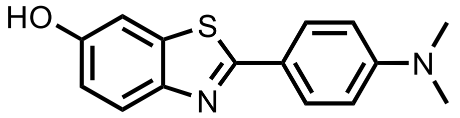

BTA-2 was prepared as previously described.[30]

2-(2-(2-((2-(4-(dimethylamino)phenyl)benzo[d]thiazol-6-yl)oxy)ethoxy)ethoxy)ethan-1-ol (**S1)**

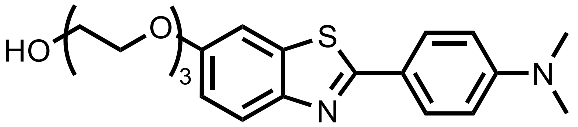

To a suspension of BTA-2 (503 mg, 1.86 mmol) and triphenylphosphine (1.21 g, 4.61 mmol, 2.5 equiv.) in anhydrous THF (100 mL) was added triethylene glycol (630 µL, 4.61 mmol, 2.5 equiv.), followed by the introduction of DIAD (911 µL, 4.63 mmol, 2.5 equiv.). The reaction mixture was stirred at room temperature for 23 h, then the solvent was evaporated under reduced pressure and the residue purified by flash chromatography (EtOAc:MeOH, 10:0 to 9:1) to afford **S1** as a white solid (495 mg, 1.23 mmol, 66%).

^{1}H NMR (600 MHz, chloroform-*d*), δ: 7.86 (d, J = 8.6 Hz, 2H), 7.83 (d, J = 8.9 Hz, 1H), 7.29 (d, J = 2.5 Hz, 1H), 7.03 (dd, J = 8.9, 2.5 Hz, 1H), 6.68 (d, J = 8.6 Hz, 2H), 4.13 (t, J = 4.8 Hz, 2H), 3.83 (t, J = 4.7 Hz, 2H), 3.73 – 3.68 (m, 4H), 3.67 – 3.64 (m, 2H), 3.58 (t, J = 4.6 Hz, 2H), 2.98 (s, 6H). **^{13}C NMR (151 MHz, chloroform-*d*), δ:** 166.6, 156.2, 151.9, 149.1, 135.7, 132.1, 132.0, 132.0, 128.6, 128.5, 128.5, 122.7, 121.5, 115.4, 111.7, 105.4, 72.6, 70.8, 70.4, 69.7, 68.0, 61.7, 40.1, 22.1, 22.0. **HRMS (ESI+)**: 403.1703 m/z: Calculated for $C_{21}H_{27}N_2O_2S^+$ = 403.1686 $[M+H]^+$. 425.1560 m/z: Calculated for $C_{21}H_{26}N_2O_2SNa^+$ = 425.1505 $[M+Na]^+$. **IR (ATR, cm^{-1})**: 2922, 2884, 1606, 1559, 1492, 1449, 1365, 1349, 1285, 1261,1223, 1188, 1138, 1126, 1101, 1068, 1043, 955, 942, 819. **MP**: 188.3-189.3 °C.

2-(2-(2-((2-(4-(dimethylamino)phenyl)benzo[d]thiazol-6-yl)oxy)ethoxy)ethoxy)ethyl 4-methylbenzenesulfonate (**S2**)

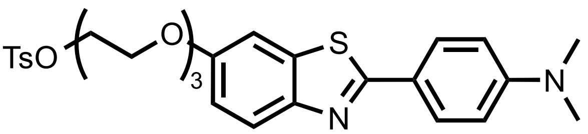

To a solution of **S1** (466 mg, 1.16 mmol, 1.0 equiv.) and triethylamine (485 µL, 3.48 mmol, 3.0 equiv.) in anhydrous dichloromethane (50 mL) at 0 °C was added a solution of tosyl chloride (664 mg, 3.48 mmol, 3.0 equiv.) in anhydrous dichloromethane (50 mL) dropwise. The resultant solution was stirred at 0 °C for 30 min then allowed to warm to room temperature, and stirred for a further 48 h. The solvent was evaporated *in vacuo* and the residue purified by flash chromatography (EtOAc:petroleum ether, 10:0 to 7:3) to afford **S2** as a yellow solid (556 mg, 1.00 mmol, 86%).

^{1}H NMR (600 MHz, chloroform-*d*), δ: 7.82 (d, *J* = 8.9 Hz, 2H), 7.78 (d, *J* = 9.0 Hz, 1H), 7.71 (d, *J* = 8.2 Hz, 2H), 7.25 – 7.21 (m, 3H), 6.97 (dd, *J* = 8.8, 2.5 Hz, 1H), 6.64 (d, *J* = 8.6 Hz, 2H), 4.10 – 4.05 (m, 4H), 3.78 – 3.74 (m, 2H), 3.60 (dt, *J* = 17.0, 5.0 Hz, 4H), 3.55 – 3.52 (m, 2H), 2.94 (d, *J* = 4.1 Hz, 6H), 2.31 (s, 3H). **^{13}C NMR (151 MHz, chloroform-*d*), δ:** 166.5, 156.2, 151.9, 149.0, 144.8, 135.7, 132.9, 129.8, 129.8, 128.4, 127.9, 122.6, 121.4, 115.4, 111.7, 105.3, 70.7, 70.7, 69.7, 69.3, 68.7, 68.7, 68.0, 40.1, 40.1, 21.6. **HRMS (ESI+)**: 557.1804 m/z: Calculated for $C_{28}H_{33}N_2O_6S_2^+$ = 557.1775 $[M+H]^+$.

4-(6-(2-(2-(2-azidoethoxy)ethoxy)ethoxy)benzo[d]thiazol-2-yl)-N,N-dimethylaniline (**S3**)

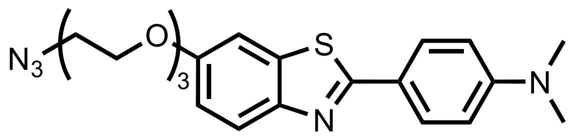

To a solution of **S2** (541 mg, 0.97 mmol, 1.0 equiv.) in anhydrous *N,N*-dimethylformamide (20 mL) was added sodium azide (252 mg, 3.88 mmmol, 4.0 equiv.) and the resultant mixture was stirred at 100 °C for 16 h. The solvent was evaporated under reduced pressure and the residue redissolved in dichloromethane (100 mL) then washed with water (2×100 mL), brine (100 mL), and dried over sodium sulfate. The solid was removed by filtration and the solvent removed *in vacuo* to afford **S3** as a yellow solid (409 mg, 0.96 mmol, 98%).

^{1}H NMR (600 MHz, chloroform-*d*), δ: 7.88 (dt, *J* = 8.7, 1.9 Hz, 2H), 7.84 (d, *J* = 8.9 Hz, 1H), 7.32 (d, *J* = 2.5 Hz, 1H), 7.04 (dd, *J* = 8.9, 2.5 Hz, 1H), 6.71 (dt, *J* = 8.9, 2.2 Hz, 2H), 4.17 (t,

J = 4.8 Hz, 2H), 3.88 (dd, J = 5.6, 3.9 Hz, 2H), 3.75 – 3.72 (m, 2H), 3.69 – 3.66 (m, 4H), 3.37 (t, J = 5.1 Hz, 2H), 3.02 (s, 6H). **^{13}C NMR (151 MHz, chloroform-*d*), δ:** 166.6, 156.3, 152.0, 149.2, 135.8, 128.6, 122.8, 121.6, 115.5, 111.8, 105.5, 71.0, 70.8, 70.2, 69.9, 68.2, 50.8, 40.2, 22.1. **HRMS (ESI+)**: 428.1739 m/z: Calculated for $C_{21}H_{26}N_5O_3S^+$ = 428.1751 $[M+H]^+$.

4-(6-(2-(2-(2-aminoethoxy)ethoxy)ethoxy)benzo[d]thiazol-2-yl)-N,N-dimethylaniline (**BTA-2-1**)

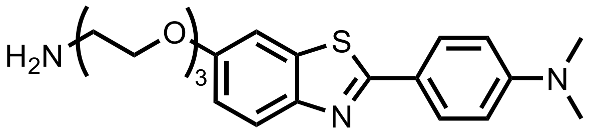

A solution of **S3** (106.9 mg, 0.250 mmol, 1.0 equiv.) and triphenylphosphine (163.9 mg, 0.625 mmol, 2.5 equiv.) were dissolved in THF (1.0 mL) and stirred at room temperature for 2 h. Water (0.1 mL) was then added and the reaction stirred at 60 °C for 17 h. Upon completion by LCMS the solvent was removed *in vacuo* and the residue was purified by RPFC on an Interchim puriFlash® C18-HP flash column (15 µm pore size, 25 g) (solvent A: H_2O, solvent B: MeCN, 5 to 100% B). Purified fractions were lyophilized to afford **BTA-2-1** as a white solid (93.8 mg, 0.233 mmol, 93%.

^{1}H NMR (400 MHz, chloroform-*d*), δ: 7.87 (dd, J = 8.8, 2.0 Hz, 2H), 7.83 (d, J = 8.9 Hz, 1H), 7.30 (d, J = 2.5 Hz, 1H), 7.03 (dd, J = 8.9, 2.5 Hz, 1H), 6.70 (dt, J = 8.8, 1.8 Hz, 2H), 4.16 (t, J = 4.8 Hz, 2H), 3.90 – 3.83 (m, 2H), 3.71 (dd, J = 5.8, 3.5 Hz, 2H), 3.63 (dd, J = 5.9, 3.5 Hz, 2H), 3.49 (t, J = 5.2 Hz, 2H), 3.00 (s, 6H), 2.84 (s, 2H). **^{13}C NMR (151 MHz, chloroform-*d*), δ:** 166.6, 156.3, 151.9, 149.1, 135.8, 128.5, 122.7, 121.6, 115.5, 111.8, 105.5, 73.3, 70.9, 70.4, 69.8, 68.2, 40.2. **HRMS (ESI+)**: 201.5971 m/z: Calculated for $C_{21}H_{29}N_3O_3S^+$ = 201.5959 $[M+2H]^{2+}$. 402.1840 m/z: Calculated for $C_{21}H_{28}N_3O_3S^+$ = 402.1846 $[M+H]^+$. 424.1654 m/z: Calculated for $C_{21}H_{27}N_3O_3SNa^+$ = 424.1665 $[M+Na]^+$. **IR (ATR, cm^{-1})**: 2872, 1605, 1559, 1530, 1489, 1447, 1359, 1319, 1286, 1260, 1222, 1188, 1168, 1121, 118, 1064, 1035, 1006, 967, 941, 910, 839, 817.

Synthesis of BTA-2-2

The synthetic route used to access **BTA-2-2** is summarized in Scheme S2.

2-(2-(2-(2-((2-(4-(dimethylamino)phenyl)benzo[d]thiazol-6-yl)oxy)ethoxy)ethoxy)ethoxy)ethan-1-ol (**S4**)

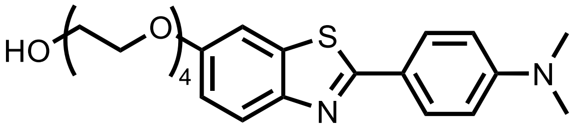

To a suspension of BTA-2 (503 mg, 1.86 mmol) and triphenylphosphine (1.21 g, 4.61 mmol, 2.5 equiv.) in anhydrous THF (100 mL) was added tetraethylene glycol (799 µL, 4.63 mmol, 2.5 equiv.) then DIAD (911 µL, 4.63 mmol, 2.5 equiv.). The reaction mixture was stirred at room temperature for 23 h, then the solvent evaporated under reduced pressure and the residue purified by flash chromatography (EtOAc:MeOH, 10:0 to 9:1) to afford **S4** as a white solid (728 mg, 1.63 mmol, 88%).

^{1}H NMR (600 MHz, chloroform-*d*), δ: 7.86 (d, J = 8.5 Hz, 2H), 7.83 (d, J = 8.8 Hz, 1H), 7.29 (s, 1H), 7.03 (d, J = 8.9 Hz, 1H), 6.68 (d, J = 8.5 Hz, 2H), 4.14 (t, J = 4.7 Hz, 2H), 3.83 (t, J = 4.7 Hz, 2H), 3.70 (d, J = 5.4 Hz, 4H), 3.65 (d, J = 4.4 Hz, 2H), 3.63 (s, 4H), 3.58 – 3.56 (m, 2H), 2.98 (s, 6H). **^{13}C NMR (151 MHz, chloroform-*d*), δ**: 166.5, 156.2, 151.8, 148.9, 135.6, 128.4, 122.6, 121.4, 115.4, 111.6, 105.3, 72.5, 70.7, 70.6, 70.5, 70.3, 69.7, 68.0, 61.6, 40.1. **HRMS (ESI+)**: 447.1951 m/z: Calculated for $C_{23}H_{31}N_2O_5S^+$ = 447.1954 $[M+H]^+$.

2-(2-(2-(2-((2-(4-(dimethylamino)phenyl)benzo[d]thiazol-6-yl)oxy)ethoxy)ethoxy)ethoxy)ethyl 4-methylbenzenesulfonate (**S5**)

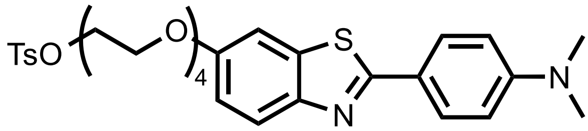

To a solution of **S4** (689 mg, 1.54 mmol, 1.0 equiv.) and triethylamine (644 µL, 4.62 mmol, 3.0 equiv.) in anhydrous dichloromethane (60 mL) at 0 °C was added a solution of tosyl chloride (881 mg, 4.62 mmol, 3.0 equiv.) in anhydrous dichloromethane (60 mL) dropwise. The resultant solution was stirred at 0 °C for 30 min then allowed to warm to room temperature, and stirred for a further 48 h. The solvent was evaporated *in vacuo* and the residue purified by flash chromatography (EtOAc:petroleum ether, 10:0 to 7:3) to afford **S5** as a yellow solid (563 mg, 0.937 mmol, 61%).

^{1}H NMR (600 MHz, chloroform-*d*), δ: 7.87 – 7.84 (m, 2H), 7.81 (d, *J* = 8.8 Hz, 1H), 7.76 – 7.73 (m, 2H), 7.29 – 7.25 (m, 3H), 7.01 (dd, *J* = 8.9, 2.6 Hz, 1H), 6.69 – 6.65 (m, 2H), 4.14 – 4.10 (m, 4H), 3.84 – 3.81 (m, 2H), 3.69 – 3.66 (m, 2H), 3.64 – 3.60 (m, 4H), 3.56 – 3.53 (m, 4H), 2.97 (s, 6H), 2.36 (s, 3H). **^{13}C NMR (151 MHz, DMSO-*d$_6$*), δ**: 166.4, 156.2, 151.8, 149.0, 144.7, 135.6, 132.9, 129.7, 128.4, 127.8, 122.6, 121.4, 115.4, 111.6, 105.3, 70.7, 70.6, 70.6, 70.4, 69.6, 69.2, 68.5, 68.0, 40.0, 22.0, 21.5. **HRMS (ESI+)**: 601.2045 m/z: Calculated for $C_{30}H_{37}N_2O_7S_2+$ = 601.2042 $[M+H]^+$

4-(6-(2-(2-(2-(2-azidoethoxy)ethoxy)ethoxy)ethoxy)benzo[d]thiazol-2-yl)-N,N-dimethylaniline (**S6**)

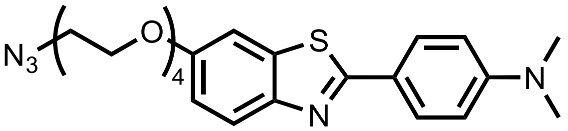

To a solution of **S5** (550 mg, 0.92 mmol, 1.0 equiv.) in anhydrous *N*,*N*-dimethylformamide (35 mL) was added sodium azide (239 mg, 3.68 mmmol, 4.0 equiv.) and the resultant mixture stirred at 100 °C for 16 h. The solvent was evaporated under reduced pressure and the residue redissolved in dichloromethane (100 mL) then washed with water (2×100 mL), brine (100 mL), and dried over sodium sulfate. The solid was removed by filtration and the solvent removed *in vacuo* to afford **S6** as a yellow solid (447 mg, 0.95 mmol, 97%).

^{1}H NMR (600 MHz, chloroform-*d*), δ: 7.90 – 7.86 (m, 2H), 7.84 (d, *J* = 8.9 Hz, 1H), 7.32 (d, *J* = 2.5 Hz, 1H), 7.04 (dd, *J* = 8.9, 2.6 Hz, 1H), 6.74 – 6.68 (m, 2H), 4.17 (dd, *J* = 5.5, 4.2 Hz, 2H), 3.88 – 3.86 (m, 2H), 3.74 – 3.72 (m, 2H), 3.69 – 3.64 (m, 8H), 3.35 (t, *J* = 5.2 Hz, 2H), 3.02 (s, 6H). **^{13}C NMR (151 MHz, chloroform-*d*), δ**: 166.6, 156.3, 152.0, 149.1, 135.8, 128.6, 122.8, 121.6, 115.5, 111.8, 105.5, 70.9, 70.8, 70.8, 70.8, 70.1, 69.8, 68.2, 50.7, 40.2. **HRMS (ESI+):** 472.2027 m/z: Calculated for $C_{23}H_{31}N_2O_5S^+$ = 472.2013 $[M+H]^+$.

Tert-butyl di(prop-2-yn-1-yl)carbamate (**S7**)

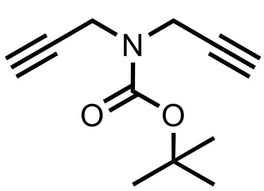

To dipropargylamine (0.83 mL, 8.00 mmol) and triethylamine (1.34 mL, 9.61 mmol) in dichloromethane (8 mL) was added di-*tert*-butyl decarbonate (2.02 mL, 8.79 mmol). The reaction solution was stirred for 70 h and the solvent removed under reduced pressure. The residue was partitioned between ethyl acetate (50 mL) and 0.5 M HCl (50 mL) and the organic layer washed with water (5 x 50 mL) and brine (50 mL). The solution was dried over sodium sulfate, filtered, and concentrated under reduced pressure to afford **S7** as an orange liquid (1.54 g, 7.97 mmol, 100%).

^{1}H NMR (600 MHz, chloroform-*d*), δ: 4.17 (s, 4H), 2.23 (t, J = 2.5 Hz, 2H), 1.48 (s, 9H). **^{13}C NMR (151 MHz, chloroform-*d*), δ:** 154.3, 85.3, 81.2, 78.9, 28.4, 27.5. **HRMS (ESI+)**: 194.1185 m/z: Calculated for $C_{11}H_{16}NO_2^+$ = 194.1181 [M+H]$^+$.

Tert-butyl bis((1-(2-(2-(2-(2-((2-(4-(dimethylamino)phenyl)benzo[d]thiazol-6-yl)oxy)ethoxy)ethoxy)ethoxy)ethyl)-1H-1,2,3-triazol-4-yl)methyl)carbamate (**S8**)

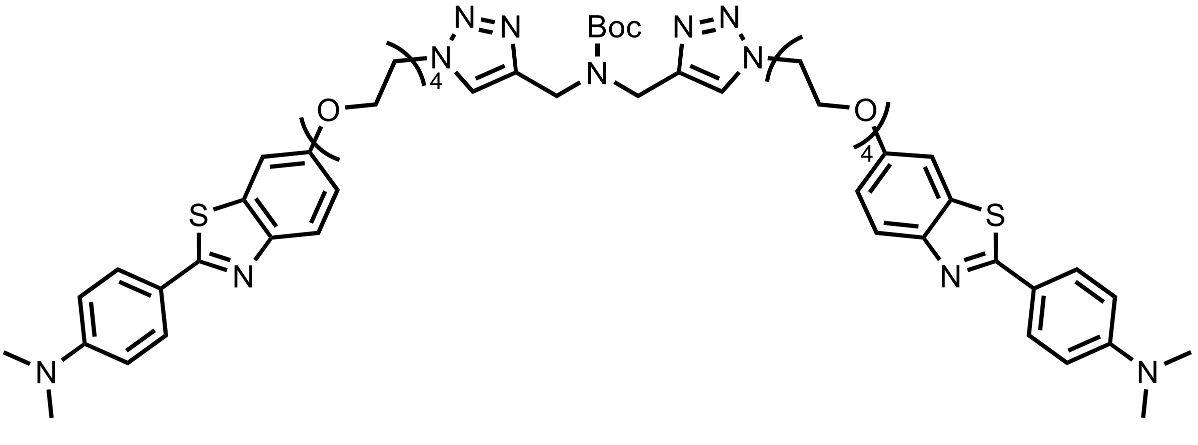

To **S6** (447 mg, 0.95 mmol, 1.00 equiv.) and **S7** (82 µL, 0.43 mmol, 0.45 equiv.) in DMF (2.5 ml) was added TBTA (50.4 mg, 0.095 mmmol, 0.10 equiv.) and tetrakis(acetonitrile)copper(I) hexafluorophosphate (35.4 mg, 0.095 mmol, 0.10 equiv.) in DMF (2.5 mL) and the reaction mixture stirred at rt for 42 h. The solvent was removed under reduced pressure and the residue redissolved in CH_2Cl_2 (100 mL). This solution was washed with an aqueous solution of 0.01 M EDTA, then 1 vol.% NH_4OH (2 x 50 mL), then brine (50 mL). The organic layer was dried over sodium sulfate, filtered, concentrated under reduced pressure, then purified by flash

chromatography (EtOAc:MeOH, 10:0 to 9:1) to afford **S8** as a yellow oil (125 mg, 0.11 mmol, 26%).

^{1}H NMR (600 MHz, chloroform-*d*), δ: 7.90 – 7.86 (m, 4H), 7.83 (d, *J* = 8.9 Hz, 2H), 7.31 (d, *J* = 2.5 Hz, 2H), 7.03 (dd, *J* = 8.9, 2.5 Hz, 2H), 6.73 – 6.69 (m, 4H), 4.55 (s, 4H), 4.48 (t, *J* = 5.1 Hz, 4H), 4.16 (dd, *J* = 5.7, 3.9 Hz, 4H), 3.86 (dd, *J* = 5.6, 3.9 Hz, 4H), 3.83 (t, *J* = 5.1 Hz, 4H), 3.72 – 3.70 (m, 4H), 3.65 – 3.62 (m, 4H), 3.61 – 3.57 (m, 8H), 3.02 (s, 12H), 1.45 (s, 9H). **^{13}C NMR (151 MHz, chloroform-*d*), δ***:* 166.6, 156.3, 155.2, 151.9, 149.1, 135.7, 128.5, 122.7, 121.6, 115.5, 111.7, 105.4, 80.3, 70.9, 70.7, 70.6, 70.6, 69.8, 69.5, 68.1, 50.2, 40.2, 28.5. **HRMS (ESI+)**: 1136.5077 m/z: Calculated for $C_{57}H_{74}N_{11}O_{10}S_2^+$ = 1136.5062 [M+H]$^+$.

4-(6-(2-(2-(2-(2-(4-((((1-(2-(2-(2-(2-((2-(4-(dimethylamino)phenyl)benzo[d]thiazol-6-yl)oxy)ethoxy)ethoxy)ethoxy)ethyl)-1H-1,2,3-triazol-4-yl)methyl)amino)methyl)-1H-1,2,3-triazol-1-yl)ethoxy)ethoxy)ethoxy)ethoxy)benzo[d]thiazol-2-yl)-N,N-dimethylaniline (**S9**)

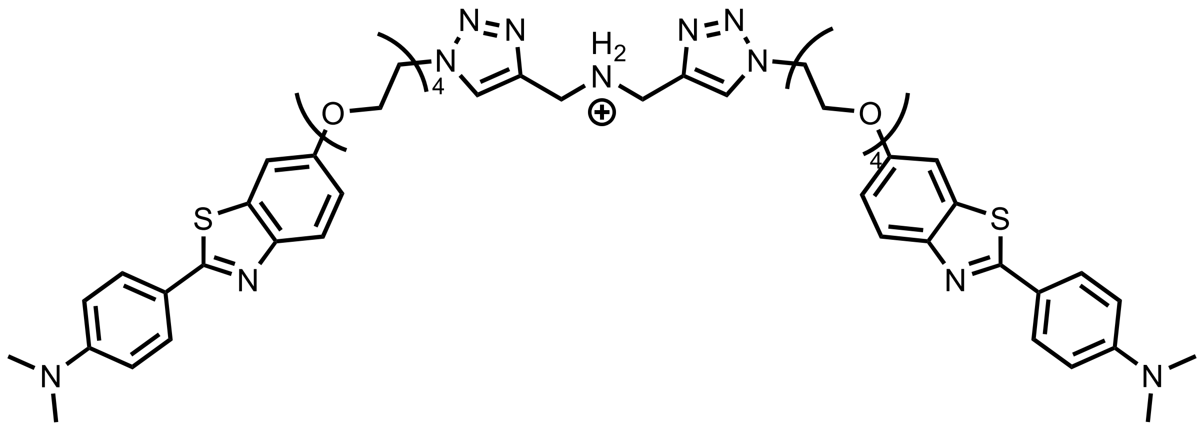

To **S8** (100.7 mg, 89 µmol, 1.0 equiv.) in CH_2Cl_2 (1.4 mL) was added 1.25 M HCl in MeOH (0.30 mL). The solution was stirred at rt for 16 h then concentrated under reduced pressure. The residue was dissolved in a minimal volume of methanol, and diethyl ether added dropwise to precipitate the product. The precipitate was recovered by filtration and dried *in vacuo* to afford **S9** as the hydrochloride salt (94.8 mg, 88 µmol, 99%).

^{1}H NMR (400 MHz, DMSO-*d$_6$*), δ: 9.77 (s, 2H), 8.22 (s, 2H), 7.81 (t, *J* = 9.1 Hz, 6H), 7.61 (d, *J* = 2.4 Hz, 2H), 7.05 (dd, *J* = 8.8, 2.4 Hz, 2H), 6.82 (d, *J* = 8.4 Hz, 4H), 4.56 (t, *J* = 4.9 Hz, 4H), 4.27 (t, *J* = 5.3 Hz, 4H), 4.14 (dd, *J* = 5.7, 3.2 Hz, 4H), 3.81 (t, *J* = 4.9 Hz, 4H), 3.75 (dd, *J* = 5.8, 3.2 Hz, 4H), 3.59 – 3.55 (m, 4H), 3.54 – 3.49 (m, 12H), 3.01 (s, 12H). **^{13}C NMR (101 MHz, DMSO-*d$_6$*), δ:** 165.4, 155.9, 151.7, 148.2, 138.0, 135.1, 128.1, 125.9, 122.3, 115.5, 112.0, 105.6, 69.9, 69.7, 69.6, 69.5, 68.9, 68.7, 67.7, 49.6, 40.6. **HRMS (ESI+)**: 1036.4529 m/z: Calculated for $C_{52}H_{66}N_{11}O_8S_2^+$ = 1036.4537 [M+H]$^+$.

Tert-butyl (2-(bis((1-(2-(2-(2-(2-((2-(4-(dimethylamino)phenyl)benzo[d]thiazol-6-yl)oxy)ethoxy)ethoxy)ethoxy)ethyl)-1H-1,2,3-triazol-4-yl)methyl)amino)-2-oxoethyl)carbamate (**S10**)

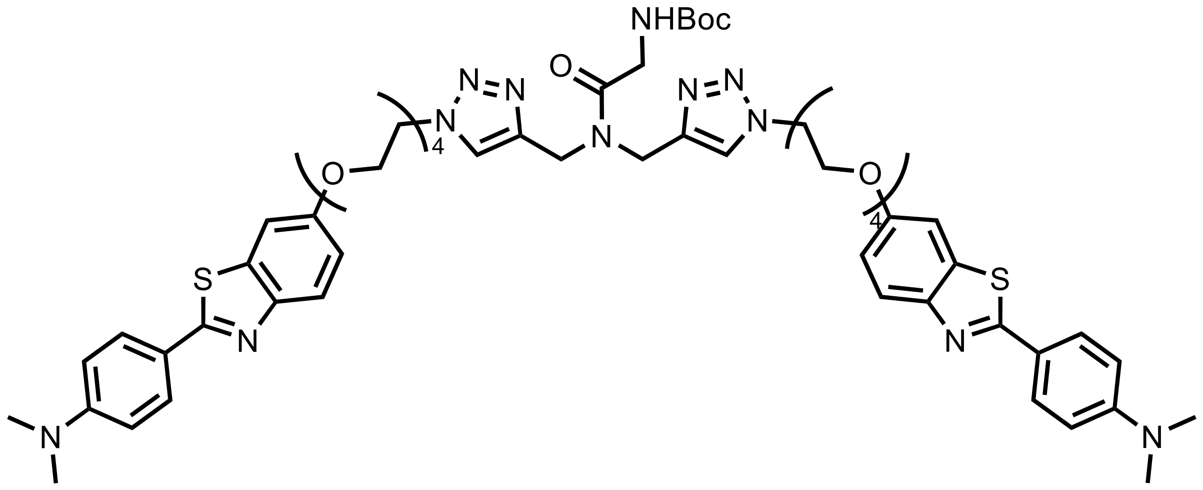

A solution of Boc-glycine (32.7 mg, 0.19 mmol, 10.0 equiv.), HBTU (91.9 mg, 0.24 mmol, 13 equiv.), and DIEA (55 µL, 0.37 mmol, 20 equiv.) in DMF (0.3 mL) was stirred at rt for 15 min. To this solution was added **S9** (20 mg, 18,6 µmol, 1.0 equiv.) in DMF (0.6 mL) dropwise. The resultant mixture was stirred at rt for 10 min. The solvent was then removed under a stream of nitrogen, and the residue purified by RPFC on an Interchim *puriFlash*® C18-HP flash column (15 µm pore size, 25 g) (solvent A: H_2O, solvent B: MeCN, 5 to 100% B). Purified fractions were lyophilised to afford **S10** as a yellow oil (13.9 mg, 11.6 µmol, 62%).

^{1}H NMR (400 MHz, chloroform-*d*), δ: 7.91 – 7.87 (m, 4H), 7.83 (d, *J* = 8.9 Hz, 2H), 7.72 (d, *J* = 17.6 Hz, 2H), 7.32 (d, *J* = 2.5 Hz, 2H), 7.03 (ddd, *J* = 8.9, 2.6, 1.0 Hz, 2H), 6.77 – 6.68 (m, 4H), 4.64 (s, 2H), 4.56 (s, 2H), 4.51 – 4.43 (m, 4H), 4.19 (dt, *J* = 9.6, 4.8 Hz, 6H), 3.87 (dd, *J* = 5.6, 4.0 Hz, 4H), 3.82 (dt, *J* = 5.1, 2.6 Hz, 4H), 3.72 (dd, *J* = 5.9, 3.4 Hz, 4H), 3.68 – 3.57 (m, 12H), 3.04 (s, 12H), 1.43 (s, 9H). **^{13}C NMR (101 MHz, chloroform-*d*), δ**: 166.7, 156.4, 152.1, 149.2, 143.5, 142.6, 135.9, 128.6, 124.4, 123.7, 122.8, 121.7, 115.6, 111.9, 105.6, 71.0, 70.8, 70.7, 70.7, 70.7, 69.9, 69.5, 68.3, 50.4, 50.3, 42.7, 41.7, 40.6, 40.3, 29.8, 28.5. **HRMS (ESI+):** 597.2693 m/z: Calculated for $C_{59}H_{76}N_{12}O_{11}S_2^{2+}$ = 597.2672 $[M+2H]^{2+}$, 608.2608 m/z: Calculated for $C_{59}H_{75}N_{12}O_{11}S_2Na^{2+}$ = 608.2581 $[M+H+Na]^{2+}$, 1193.5267 m/z: Calculated for $C_{59}H_{75}N_{12}O_{11}S_2^{2+}$ = 1193.5271 $[M+H]^{+}$, 1215.5068 m/z: Calculated for $C_{59}H_{74}N_{12}O_{11}S_2Na^{2+}$ = 1215.509 $[M+Na]^{+}$.

2-(bis((1-(2-(2-(2-(2-((2-(4-(dimethylamino)phenyl)benzo[d]thiazol-6-yl)oxy)ethoxy)ethoxy)ethoxy)ethyl)-1H-1,2,3-triazol-4-yl)methyl)amino)-2-oxoethan-1-aminium hydrochloride (**BTA-2-2**)

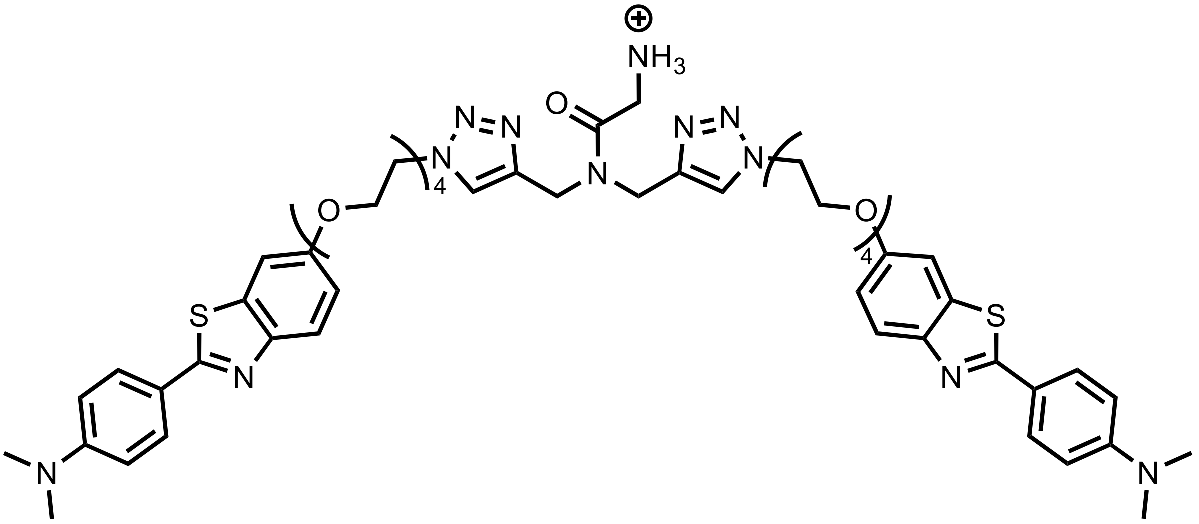

To **S10** (17.9 mg, 14.9 µmol, 1.0 equiv.) in CH_2Cl_2 (2.0 mL) was added 1.25 M HCl in MeOH (1. mL). The solution was stirred at rt for 16 h then concentrated under reduced pressure. The residue was dissolved in a minimal volume of methanol, and diethyl ether added dropwise to precipitate the product. The precipitate was recovered by filtration and dried *in vacuo* to afford **BTA-2-2** as the hydrochloride salt (16.2 mg, 14.3 µmol, 96%).

^{1}H NMR (400 MHz, DMSO-*d*$_6$), δ: 8.17 (t, *J* = 5.7 Hz, 3H), 8.11 (s, 1H), 7.97 (s, 1H), 7.81 (dd, *J* = 11.4, 8.5 Hz, 6H), 7.63 – 7.57 (m, 2H), 7.05 (d, *J* = 8.2 Hz, 2H), 6.83 (d, *J* = 8.3 Hz, 4H), 4.57 (d, *J* = 17.4 Hz, 4H), 4.49 (dt, *J* = 9.7, 4.9 Hz, 6H), 4.18 – 4.11 (m, 6H), 3.79 (t, *J* = 5.0 Hz, 3H), 3.75 (t, *J* = 4.1 Hz, 3H), 3.59 – 3.48 (m, 16H), 3.00 (s, 12H). **^{13}C NMR (101 MHz, DMSO-*d*$_6$), δ**: 165.4, 155.9, 148.1, 135.1, 128.1, 122.3, 115.5, 112.1, 106.6, 105.6, 69.9, 69.7, 69.6, 69.5, 69.5, 68.9, 68.6, 67.7. **HRMS (ESI+)**: 1093.4772 m/z: Calculated for $C_{54}H_{69}N_{12}O_9S_2^+$ = 1093.4752 [M+H]$^+$.

Synthesis of BF-79-1

The synthetic route used to access **BF-79-1** is summarized in Scheme S3.

1-(4-nitrophenyl)-4-(pyridin-2-yl)piperazine (**S11**)

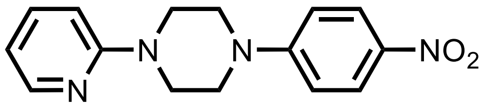

S11 was synthesized based on a previously reported procedure.[21] To 1-chloro-4-nitrobenzene (500 mg, 3.17 mmol, 1.0 equiv.), potassium tertbutoxide (711 mg, 6.34 mmol, 2 equiv.), tris(dibenzylideneacetone)dipalladium (72 mg, 0.079 mmol, 0.02 equiv.), and RuPhos (74 mg, 0.05 equiv., 0.158 mmol) was added 1-(pyridin-2-yl)piperazine (1.03 g, 6.34 mmol, 2.0 equiv.) then 1,4-dioxane (10 mL) under an inert atmosphere. The reaction mixture was heated to 100 °C and stirred vigorously for 30 min. Upon completion by LCMS, the reaction mixture was cooled to rt and filtered through celite then the solvent removed in vacuo. The crude product was then purified using silica column chromatography (CH_2Cl_2 to CH_2Cl_2:MeOH 95:5) to afford **S11** as a brown solid (556 mg, 1.95 mmol, 62%).

^{1}H NMR (400 MHz, chloroform-*d*), δ: 8.22 (ddd, *J* = 5.0, 2.0, 1.0 Hz, 1H), 8.19 – 8.10 (m, 2H), 7.54 (ddd, *J* = 8.9, 7.2, 2.0 Hz, 1H), 6.85 (dt, *J* = 9.4, 3.5 Hz, 2H), 6.71 – 6.67 (m, 2H), 3.80 – 3.73 (m, 4H), 3.63 – 3.56 (m, 4H). **^{13}C NMR (101 MHz, chloroform-*d*), δ:**158.7, 154.7, 147.9, 138.7, 138.0, 126.2, 114.0, 112.6, 107.3, 46.7, 44.7. **HRMS (ESI+):** 285.1356 m/z: Calculated for $C_{15}H_{17}N_4O_2^+$ = 285.1352 $[M+H]^+$. **IR (ATR, cm^{-1}):** 2850, 1591, 1478, 1437, 1388, 1317, 1234, 1158, 1116, 1090, 1037, 979, 951, 906, 825. **Decomposition point:** decolorization at 131 °C, melted at 172-184 °C. Characterization data is in agreement with that reported by Ferrie et al.[2]

6-fluoro-N-(4-(4-(pyridin-2-yl)piperazin-1-yl)phenyl)nicotinamide (**S12**)

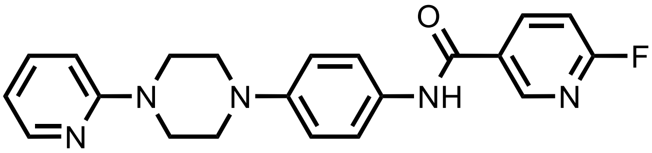

S12 was synthesized based on a previously reported procedure.[21] To an orange suspension of **S1** (509.0 mg, 2.00 mmol, 1.0 equiv.) in methanol (6.0 mL) was added Pd/C (10 wt.%, 20 mg, 0.2 mmol, 0.1 equiv.) under an inert atmosphere at rt. The suspension was stirred under a H_2 atmosphere at rt for 16 h. The magenta reaction mixture was then filtered through celite which was washed with methanol (2 x 50 mL). The orange filtrate was then evaporated in vacuo and the reduced intermediate was redissolved in dry DMF (4 mL, 2 mL/mmol).

To a solution of 6-fluoronicotinic acid (366.5 mg, 2.6 mmol, 1.3 equiv.) and HATU (990.0 mg, 2.6 mmol, 1.3 equiv.) in dry DMF (8 mL, 4 mL/mmol) at 0 °C was added DIEA (1.05 mL, 6 mmol, 3 equiv.). The reaction mixture was stirred for 5 min, and the solution of reduced intermediate was added dropwise. The resultant orange solution was stirred for 20 min as a pale beige precipitate formed, and was then warmed to rt and stirred for a further 6 h. The reaction mixture was then diluted with water (100 mL) and extracted with EtOAc (3 x 100 mL). The combined organic extracts were washed with brine (100 mL), dried over anhydrous sodium sulfate, filtered, and then concentrated *in vacuo*. The crude product was then purified using silica column chromatography (PE to EtOAc:PE 4:1) to afford **S12** as a white solid (515.9 mg, 1.38 mmol, 71% over two steps).

^{1}H NMR (400 MHz, DMSO-*d$_6$*), δ: 10.27 (s, 1H), 8.80 (d, *J* = 2.5 Hz, 1H), 8.48 (td, *J* = 8.2, 2.6 Hz, 1H), 8.14 (dd, *J* = 4.9, 2.0 Hz, 1H), 7.62 (dt, *J* = 9.1, 2.2 Hz, 2H), 7.56 (ddd, *J* = 8.9, 7.0, 2.1 Hz, 1H), 7.35 (dd, *J* = 8.6, 2.7 Hz, 1H), 7.02 (dt, *J* = 9.0, 2.1 Hz, 2H), 6.90 (d, *J* = 8.6 Hz, 1H), 6.67 (dd, *J* = 7.1, 4.9 Hz, 1H), 3.63 (dd, *J* = 6.6, 3.8 Hz, 4H), 3.22 (dd, *J* = 6.3, 4.0 Hz, 4H). **^{13}C NMR (101 MHz, DMSO-*d$_6$*), δ:** 165.4, 163.0, 162.2, 159.0, 147.7, 147.7, 147.6, 147.5, 141.8, 141.7, 137.6, 130.8, 129.5, 129.4, 121.5, 115.9, 113.2, 109.6, 109.2, 107.3, 48.5, 44.6. **HRMS (ESI+):** 378.1740 m/z: Calculated for $C_{21}H_{21}FN_5O^+$ = 378.173 $[M+H]^+$. **IR (ATR, cm^{-1}):** 3263, 2838, 1639, 1599, 1535, 1484, 1439, 1311, 1231, 1161, 953, 811, 776, 733.

6-(2-(2-(2-(2-hydroxyethoxy)ethoxy)ethoxy)ethoxy)-N-(4-(4-(pyridin-2-yl)piperazin-1-yl)phenyl)nicotinamide (**S13**)

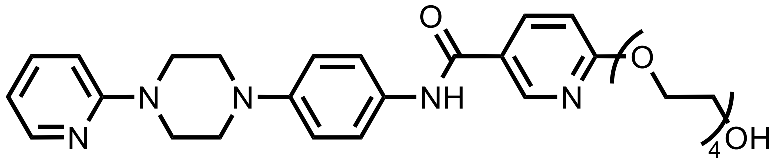

A solution of **S12** (505.7 mg, 1.34 mmol, 1.0 equiv.), tetraethylene glycol (2.32 mL, 13.4 mmol, 10.0 equiv.), and potassium *tert*-butoxide (1.504 g, 13.4 mmol, 10.0 equiv.) was dissolved in acetonitrile (15 mL). The resultant orange solution was heated at 100 °C in a microwave for 30 min. The solvent was evaporated under reduced pressure, and the residue was redissolved in CH_2Cl_2. This solution was washed with water (3 x 50 mL), brine (50 mL), dried over sodium sulfate, filtered, and then concentrated *in vacuo*. The crude residue was purified by silica column chromatography (PE to EtOAc:PE 4:1) to afford **S13** as a yellow solid (680.6 mg, 1.23 mmol, 92%).

^{1}H NMR (400 MHz, DMSO-*d$_6$*), δ: 10.09 (s, 1H), 8.75 (d, *J* = 2.5 Hz, 1H), 8.23 (dd, *J* = 8.7, 2.6 Hz, 1H), 8.11 (dd, *J* = 5.6, 2.0 Hz, 1H), 7.71 (s, 1H), 7.63 (dt, *J* = 8.9, 2.3 Hz, 2H), 7.08 (s, 1H), 7.02 (d, *J* = 8.7 Hz, 2H), 6.95 (dd, *J* = 8.8, 1.7 Hz, 1H), 6.77 (d, *J* = 6.6 Hz, 1H), 4.49 – 4.42 (m, 2H), 4.13 – 4.05 (m, 1H), 3.79 – 3.75 (m, 2H), 3.70 (d, *J* = 5.8 Hz, 4H), 3.59 (dd, *J* = 5.6, 3.1 Hz, 2H), 3.56 – 3.46 (m, 8H), 3.41 (dd, *J* = 5.5, 4.3 Hz, 2H), 3.25 (t, *J* = 5.2 Hz, 4H). **^{13}C NMR (101 MHz, DMSO-*d$_6$*), δ:** 170.3, 164.8, 163.1, 147.4, 147.2, 138.6, 131.2, 124.4, 121.5, 115.9, 113.2, 110.3, 110.2, 72.3, 69.9, 69.9, 69.8, 69.8, 69.7, 69.7, 68.6, 68.2, 65.4, 63.2, 60.2, 48.5, 44.7, 40.1, 39.9, 39.7, 39.5, 39.3, 39.1, 38.9, 20.7. **HRMS (ESI+):** 552.2823 m/z: Calculated for $C_{29}H_{38}N_5O_6^+$ = 552.2822 $[M+H]^+$. **IR (ATR, cm^{-1}):** 3294, 2920, 2358, 1737, 1637, 1603, 1530, 1487, 1438, 1348, 1291, 1232, 1122, 1051.

6-(2-(2-(2-(2-aminoethoxy)ethoxy)ethoxy)ethoxy)-N-(4-(4-(pyridin-2-yl)piperazin-1-yl)phenyl)nicotinamide (**S15**)

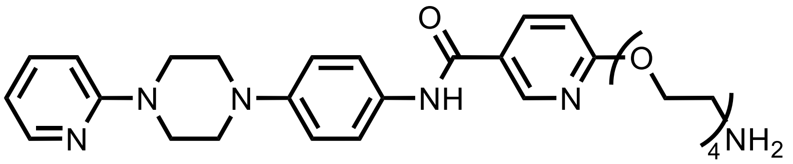

To a solution of **S13** (664.7 mg, 1.21 mmol, 1.0 equiv.) in anhydrous CH_2Cl_2 (8 mL) on ice was added triethylamine (102 µL, 1.33 mmol, 1.1 equiv.) then methanesulfonyl chloride (252 µL, 1.81 mmol, 1.50 equiv.). The resultant orange solution was stirred at 0 °C for 1 h, then warmed to room temperature.and stirred for a further 30 min. To the reaction mixture was added ice-cold water (50 mL), and the aqueous layer was washed with CH_2Cl_2 (3 x 50 mL). The combined organic extracts were washed with brine (50 mL), dried over sodium sulfate, then evaporated under reduced pressure to yield the crude mesylate intermediate.

This intermediate was then redissolved in anhydrous DMF (4 mL) to which was added sodium azide (258.6 mg, 3.3 equiv.). The reaction was stirred at 60 °C for 18 h. Upon completion by LCMS the solvent was removed under a stream of nitrogen. The residue was redissolved in CH_2Cl_2 (100 mL) then washed with water (5 x 100 mL), brine (100 mL), and dried over sodium sulfate. The solvent was removed under reduced pressure to afford the azide derivative **S14**.

A solution of the azide derivative (588.2 mg, 1.02 mmol, 1.0 equiv.) and triphenylphosphine (294.3 mg, 1.12 mmol, 1.1 equiv.) in THF (25 mL) was stirred at 50 °C for 2 h. Water (0.5 mL) was then added and the reaction was stirred at 50 °C for a further 11 h. Upon completion by LCMS, the solvent was removed under reduced pressure and the product was purified by RPFC on a Interchim *puriFlash*® C18-HP flash column (15 µm pore size, 25 g) (solvent A: H_2O + 0.1% FA, solvent B: MeCN + 0.1% FA, 0 to 50% B). Purified fractions were lyophilised. The residue was redissolved in CH_2Cl_2 (100 mL) and the organic layer was washed with saturated sodium bicarbonate (2 x 100 mL), then 1:1 brine:saturated sodium bicarbonate (100 mL), before being dried over sodium sulfate and concentrated under reduced pressure to afford **S15** as a white solid (562.1 mg, 1.02 mmol, 84%).

^{1}H NMR (400 MHz, DMSO-*d$_6$*), δ: 10.08 (s, 1H), 8.75 (d, *J* = 2.6 Hz, 1H), 8.22 (dd, *J* = 8.6, 2.5 Hz, 1H), 8.14 (dd, *J* = 5.0, 2.0 Hz, 1H), 7.68 – 7.59 (m, 2H), 7.56 (ddd, *J* = 8.9, 7.0, 2.0 Hz, 1H), 7.05 – 6.97 (m, 2H), 6.95 (d, *J* = 8.7 Hz, 1H), 6.89 (d, *J* = 8.5 Hz, 1H), 6.67 (dd, *J* = 7.2, 4.9 Hz, 1H), 4.50 – 4.41 (m, 2H), 3.79 – 3.73 (m, 2H), 3.65 – 3.47 (m, 12H), 3.35 (t, *J* =

5.8 Hz, 2H), 3.20 (t, J = 5.3 Hz, 4H), 2.64 (t, J = 5.8 Hz, 2H). **^{13}C NMR (101 MHz, chloroform-*d*), δ:** 164.8, 163.1, 159.0, 147.6, 147.5, 147.2, 138.6, 137.6, 131.2, 124.4, 121.5, 115.9, 113.2, 110.3, 107.3, 72.9, 69.9, 69.8, 69.8, 69.6, 68.7, 65.4, 48.6, 44.6, 41.3, 40.1, 39.9, 39.7, 39.5, 39.3, 39.1, 38.9. **HRMS (ESI+):** 551.2990 m/z: Calculated for $C_{29}H_{39}N_6O_5^+$ = 551.2982 $[M+H]^+$. **IR (ATR, cm^{-1}):** 3301, 2862, 1636, 1601, 1592, 1561, 1527, 1516, 1484, 1454, 1436, 1415, 1387, 1347, 1313, 1290, 1365, 1244, 1229, 1097, 1039, 980, 949, 899, 839, 815.

6-(2-(2-(2-(2-((4,6-bis((2-aminoethyl)amino)-1,3,5-triazin-2-yl)amino)ethoxy)ethoxy)ethoxy)ethoxy)-N-(4-(4-(pyridin-2-yl)piperazin-1-yl)phenyl)nicotinamide (**BF-79-1**)

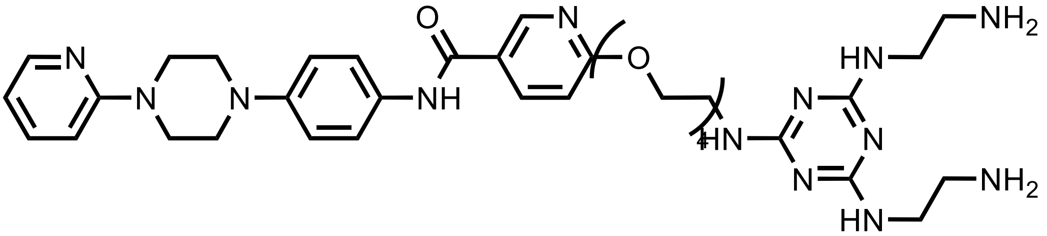

A solution of **S15** (11.4 mg, 0.021 mmol, 1.0 equiv.) in CH_2Cl_2 (0.20 mL) was added dropwise to a solution of cyanuric chloride (3.9 mg, 0.021 mmol, 1.0 equiv.) in CH_2Cl_2 (0.20 mL) at 0 °C. DIEA (14.6 µL, 0.084 mmol, 4.0 equiv.) was then added dropwise. The resultant solution was stirred at 0 °C for 2 h. Upon complete conversion to the mono-substituted triazine by LCMS, ethylene diamine (28 µL, 0.42 mmol, 20.0 equiv.) was added to the reaction solution at 0 °C. The reaction was then warmed to rt and stirred for 2 h. The reaction was then heated to 50° C under microwave irradiation for 50 min to afford complete conversion to the desired product. The solvent was removed *in vacuo*, and the crude reaction mixture was purified by reverse phase flash chromatography on an Interchim *puriFlash*® C18-HP flash column (15 µm pore size, 4 g) (solvent A: H_2O + 0.1% FA, solvent B: MeCN + 0.1% FA, 0 to 10% B) then normal phase flash chromatography (CH_2Cl_2 : 7 N NH_3 in MeOH, 0 to 100%) to afford **BF-79-1** as an off-white solid (9.0 mg, 0.012 mmol, 57%).

^{1}H NMR (400 MHz, *DMSO-d$_6$*), δ: 10.18 (s, 1H), 8.78 (d, J = 2.5 Hz, 1H), 8.26 (dt, J = 8.7, 2.3 Hz, 1H), 8.13 (dd, J = 4.9, 2.0 Hz, 1H), 7.69 – 7.61 (m, 2H), 7.56 (ddd, J = 8.9, 7.0, 2.0 Hz, 1H), 7.01 – 6.97 (m, 2H), 6.94 (d, J = 8.7 Hz, 1H), 6.90 (d, J = 8.7 Hz, 1H), 6.66 (dd, J = 7.1, 4.9 Hz, 1H), 4.48 – 4.39 (m, 2H), 3.98 (s, 1H), 3.79 – 3.74 (m, 2H), 3.63 (t, J = 5.1 Hz, 4H), 3.59 – 3.56 (m, 2H), 3.54 – 3.50 (m, 6H), 3.20 (dd, J = 6.4, 3.9 Hz, 4H), 2.94 (s, 2H), 2.84 (dt,

J = 11.0, 6.4 Hz, 2H). **^{13}C NMR (176 MHz, DMSO-*d$_6$*), δ:** 170.0, 164.8, 163.1, 161.8, 159.0, 147.6, 147.5, 147.3, 138.7, 137.6, 131.2, 124.4, 121.5, 115.9, 115.9, 113.2, 110.2, 107.3, 69.9, 69.8, 69.8, 69.6, 68.7, 65.4, 63.6, 48.6, 44.6, 38.6, 38.3, 36.5, 35.1, 22.6. **HRMS (ESI+):** 768.4013 m/z: Calculated for $C_{36}H_{51}N_{13}O_5Na^+$ = 768.4029 [M + Na]$^+$. 384.7062 m/z: Calculated for $C_{36}H_{52}N_{13}O_5Na^{2+}$ = 384.7051 [M + H + Na]$^+$.

Synthesis of BF-79-2

6,6'-((((((((((6-((2-aminoethyl)amino)-1,3,5-triazine-2,4-diyl)bis(azanediyl))bis(ethane-2,1-diyl))bis(oxy))bis(ethane-2,1-diyl))bis(oxy))bis(ethane-2,1-diyl))bis(oxy))bis(ethane-2,1-diyl))bis(oxy))bis(N-(4-(4-(pyridin-2-yl)piperazin-1-yl)phenyl)nicotinamide) (**BF-79-2**)

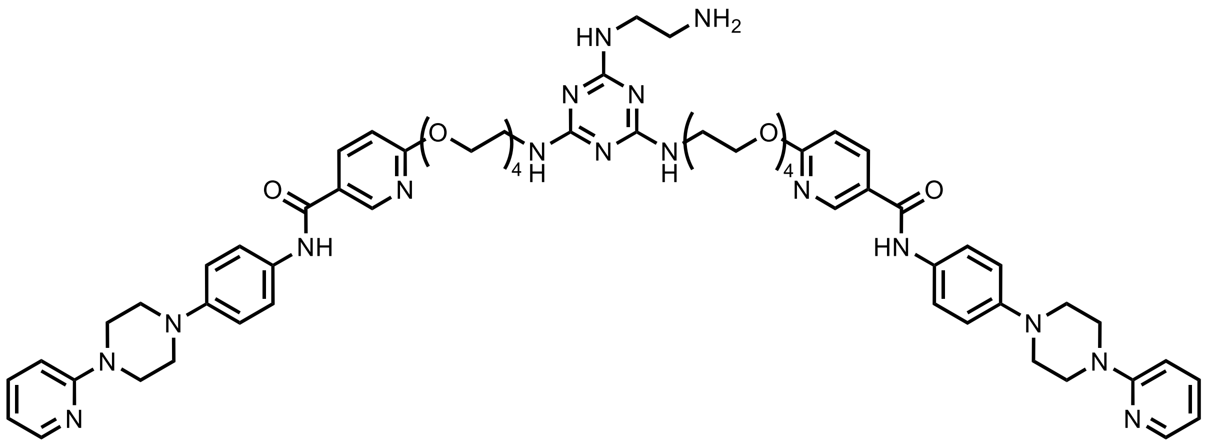

A solution of the amine **S15** (111 mg, 0.202 mmol, 2.7 equiv.), cyanuric chloride (13.7 mg, 0.074 mmol, 1.0 equiv.), and DIEA (52 µL, 0.30 mmol, 4.0 equiv.) in anhydrous THF (1.0 mL) and DMF (0.2 mL) was stirred at rt for 20 h. Upon complete reaction by LCMS, ethylene diamine (200 µL, 3.0 mmol, 40 equiv.) was added and the reaction was heated at 50 °C for 100 min in the microwave. Upon completion by LCMS, the solvent was removed under a stream of nitrogen and the product was purified by RPFC on an Interchim *puriFlash*® C18-HP flash column (15 µm pore size, 25 g) (solvent A: H_2O + 0.1% FA, solvent B: MeCN + 0.1% FA, 0 to 50% B). Purified fractions were lyophilized to afford **BF-79-2** as a white solid (38.3 mg, 0.031 mmol, 42%).

^{1}H NMR (700 MHz, 9:1 chloroform-*d*:methanol-*d$_4$*), δ: 8.60 (s, 2H), 8.11 (d, *J* = 5.0 Hz, 2H), 8.05 (d, *J* = 8.7 Hz, 2H), 7.50 (d, *J* = 8.4 Hz, 4H), 7.48 – 7.45 (m, 2H), 6.92 (d, *J* = 8.6 Hz, 4H), 6.75 (d, *J* = 8.6 Hz, 2H), 6.67 (d, *J* = 8.6 Hz, 2H), 6.61 (t, *J* = 6.1 Hz, 2H), 4.44 (t, *J* = 4.4 Hz, 4H), 3.80 (t, *J* = 4.7 Hz, 4H), 3.71 – 3.32 (m, 36H), 3.31 (h, *J* = 1.5 Hz, 6H), 2.82 (s, 2H). **^{13}C NMR (176 MHz, 9:1 chloroform-*d*:methanol-*d$_4$*), δ:** 165.4, 164.5, 159.5, 148.3, 147.7, 146.7, 138.4, 137.9, 131.0, 124.5, 122.1, 117.0, 113.8, 111.0, 107.8, 70.6, 70.6, 70.3, 70.0, 69.5, 65.8, 49.7, 49.6, 49.4, 49.3, 49.2, 49.1, 48.8, 45.4, 41.3, 40.3. **HRMS (ESI+):** 1236.6433 m/z: Calculated for $C_{63}H_{82}N_{17}O_{10}^{2+}$ = 1236.6431 [M+H]$^+$. **IR (ATR, cm^{-1}):** 3297, 2870, 1637, 1592, 1517, 1486, 1437, 1387, 1347, 1291, 1232, 1120, 1042, 981, 951, 813, 774, 730.

NMR Spectra

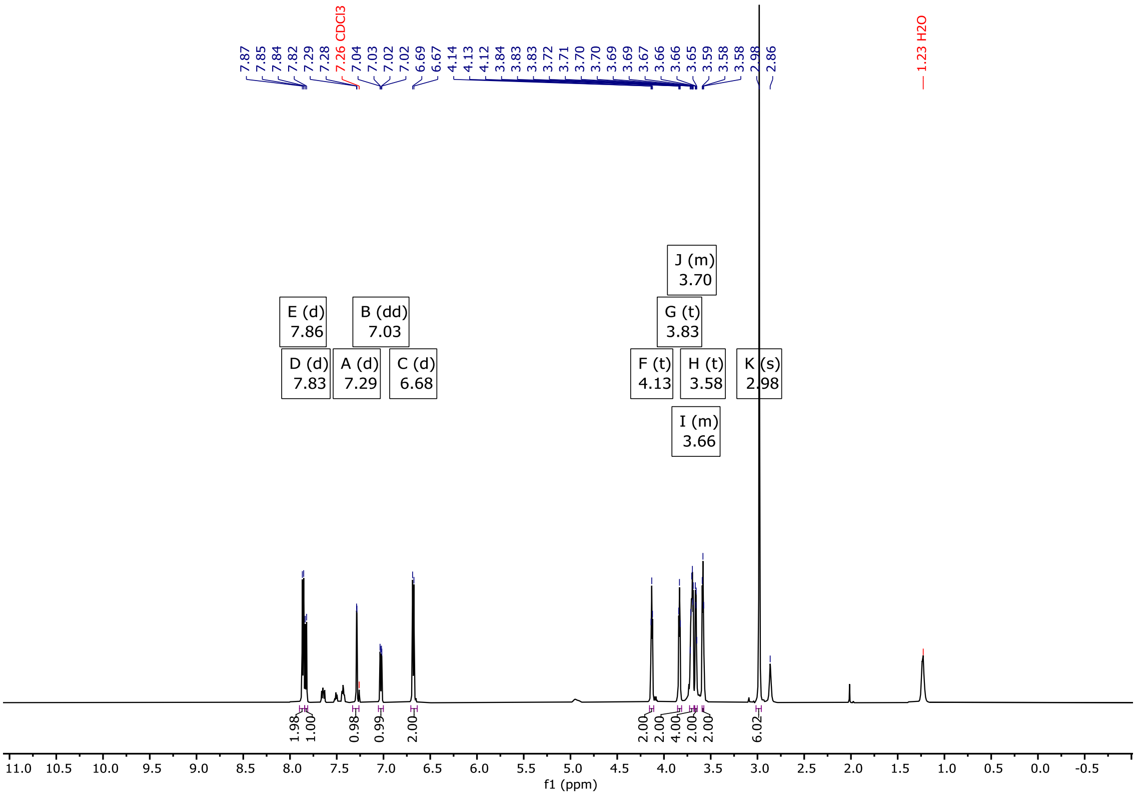

Figure S1. ^{1}H NMR (600 MHz, chloroform-*d*) spectra of **S1.**

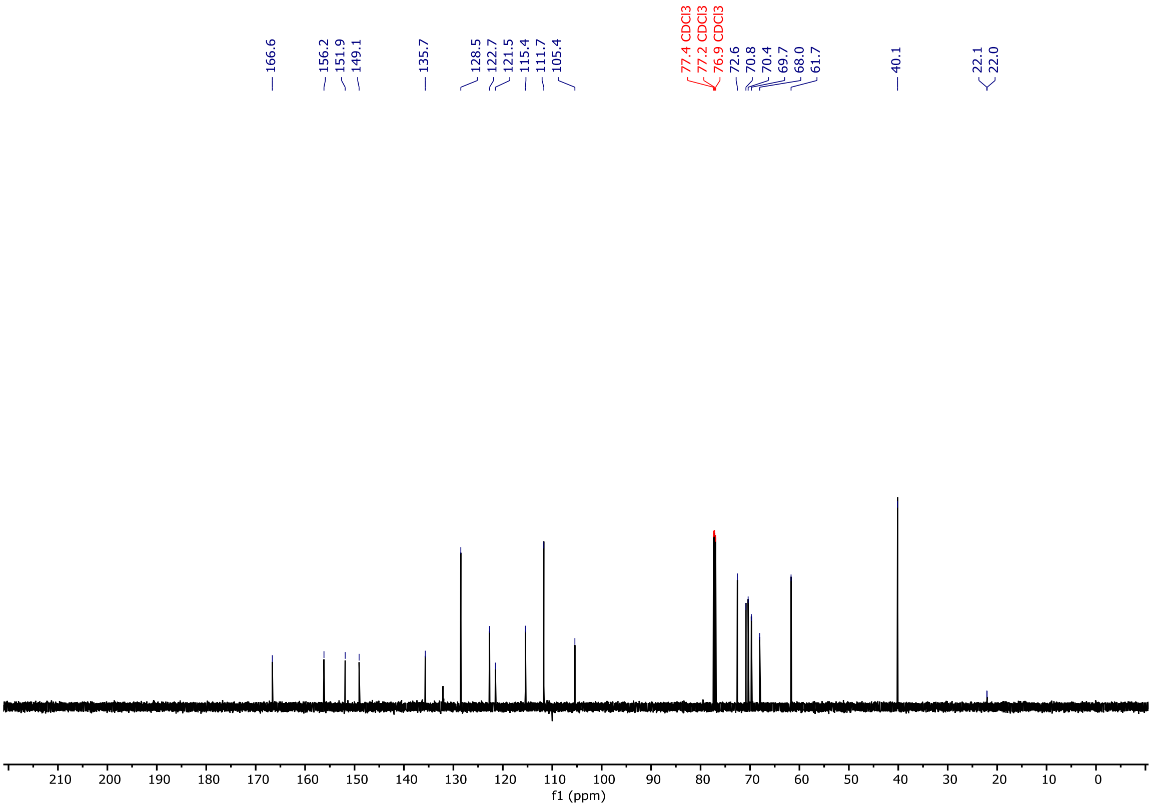

Figure S2. ^{13}C NMR (151 MHz, chloroform-*d*) spectra of **S1.**

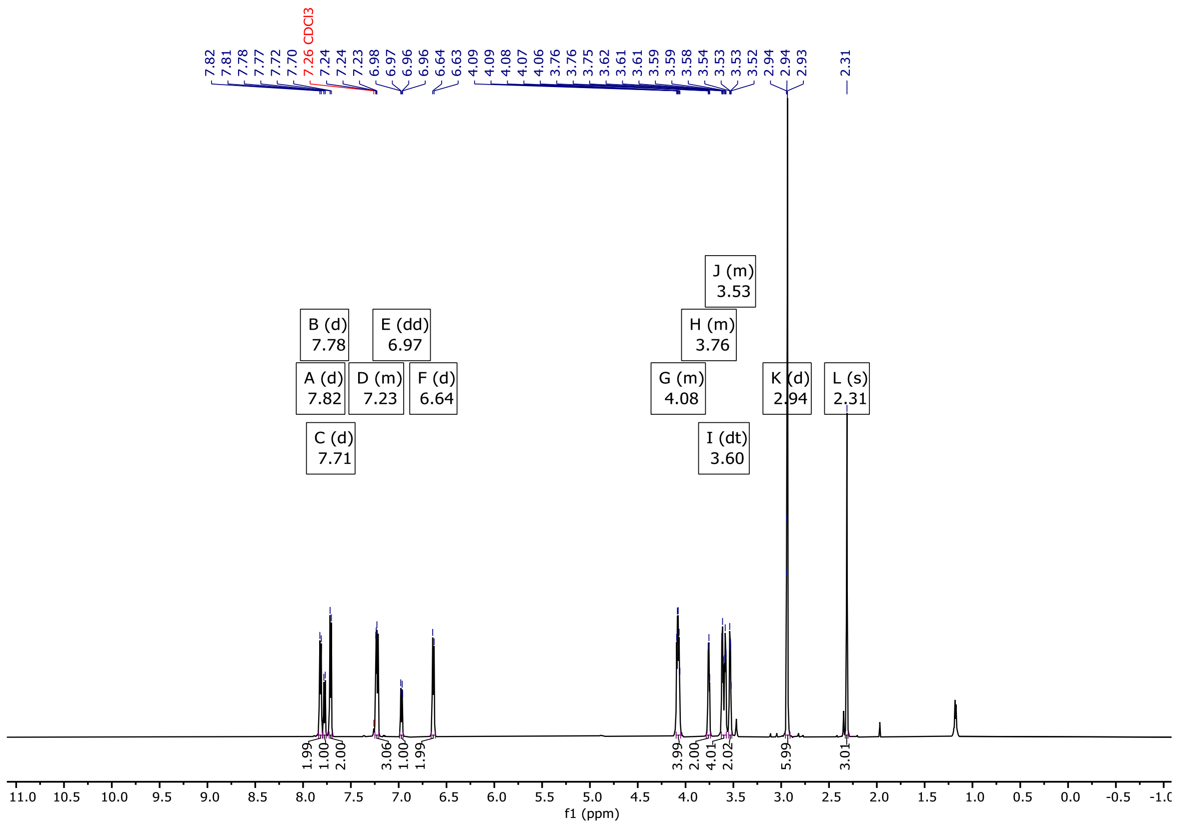

Figure S3. ^{1}H NMR (600 MHz, chloroform-*d*) spectra of **S2.**

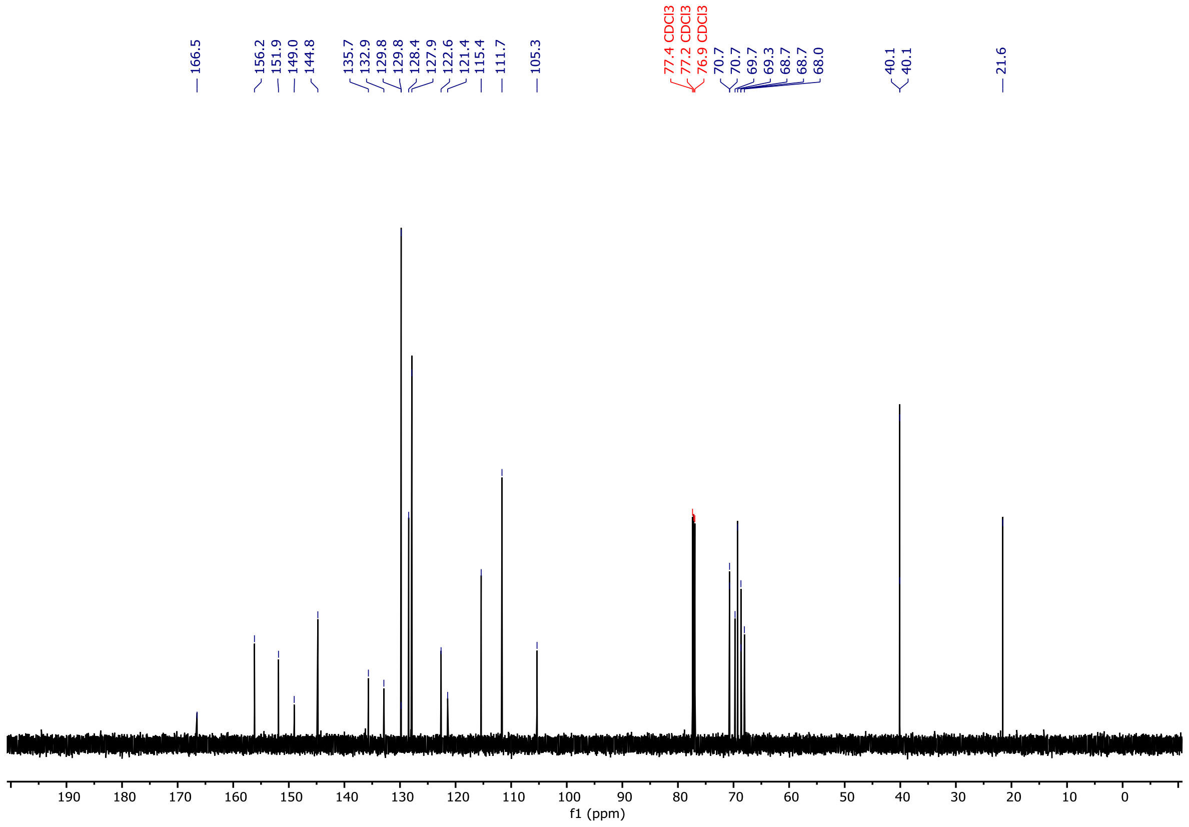

Figure S4. ^{13}C NMR (151 MHz, chloroform-*d*) spectra of **S2.**

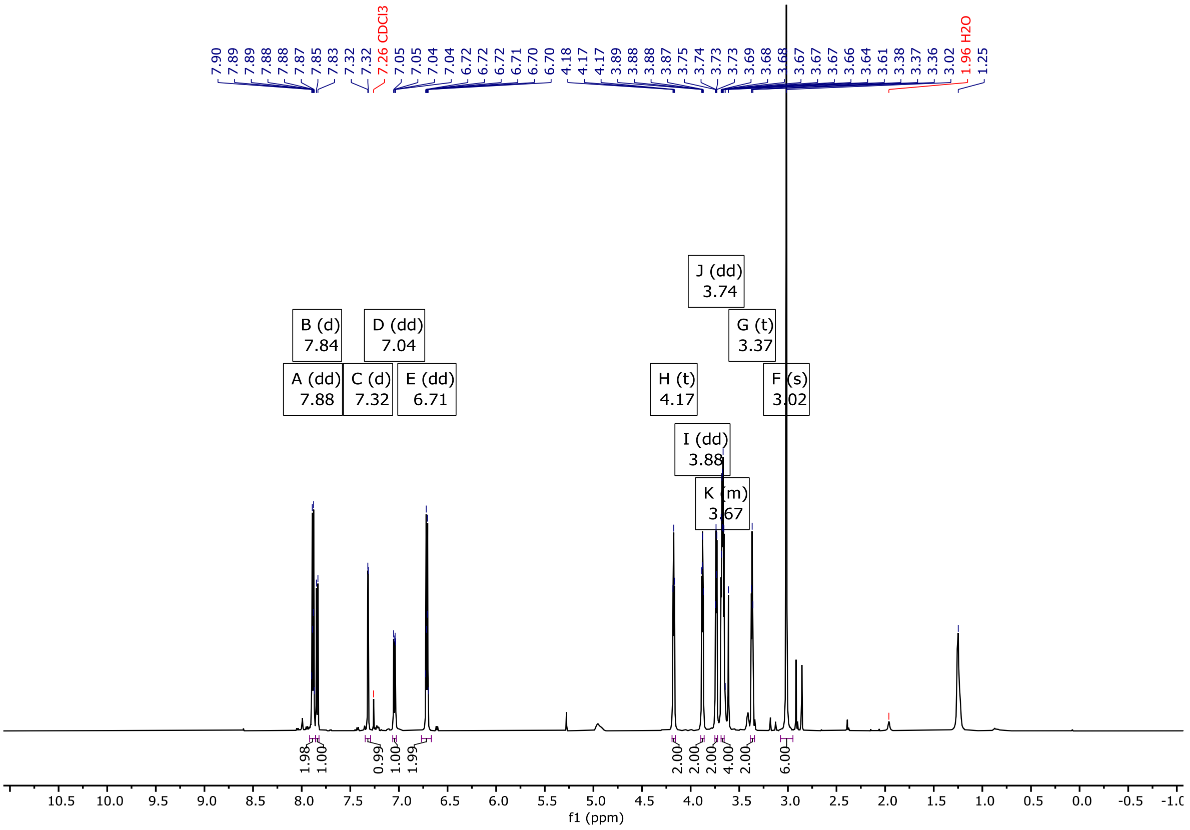

Figure S5. ^{1}H NMR (600 MHz, chloroform-*d*) spectra of **S3.**

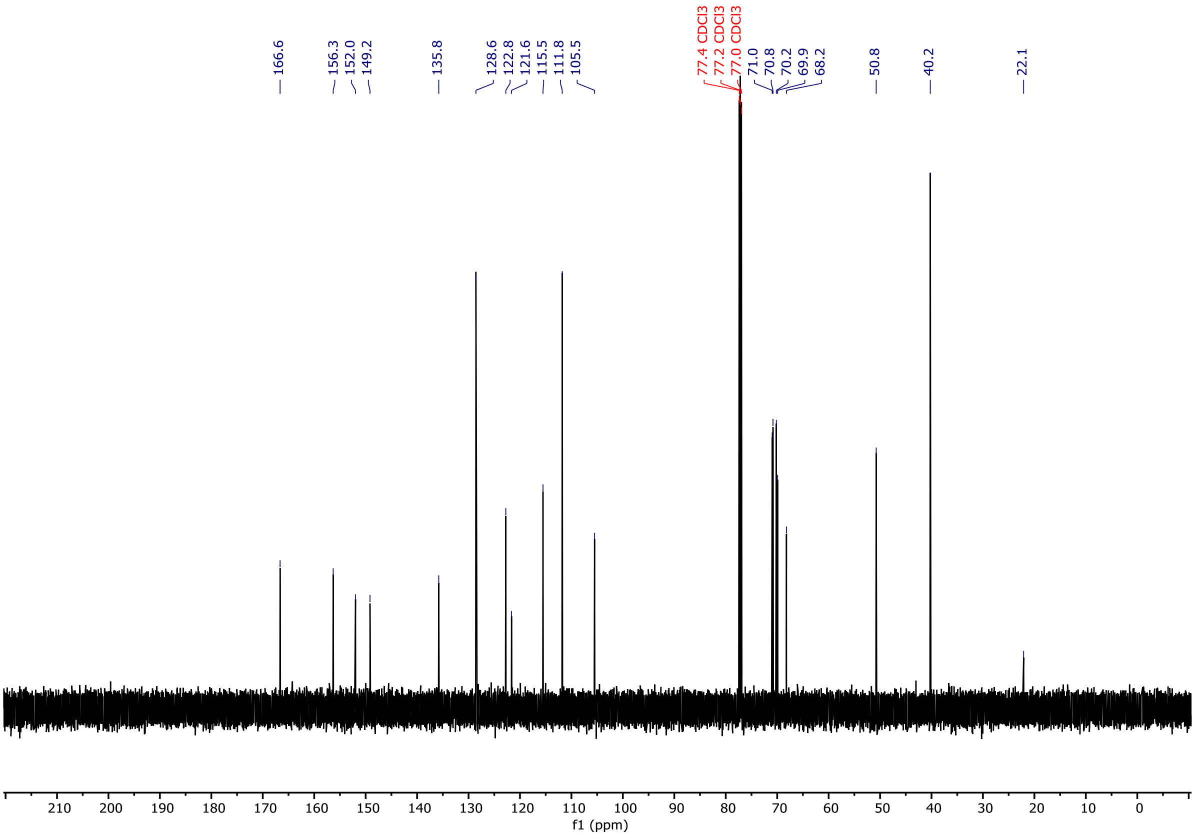

Figure S6. ^{13}C NMR (151 MHz, chloroform-*d*) spectra of **S3.**

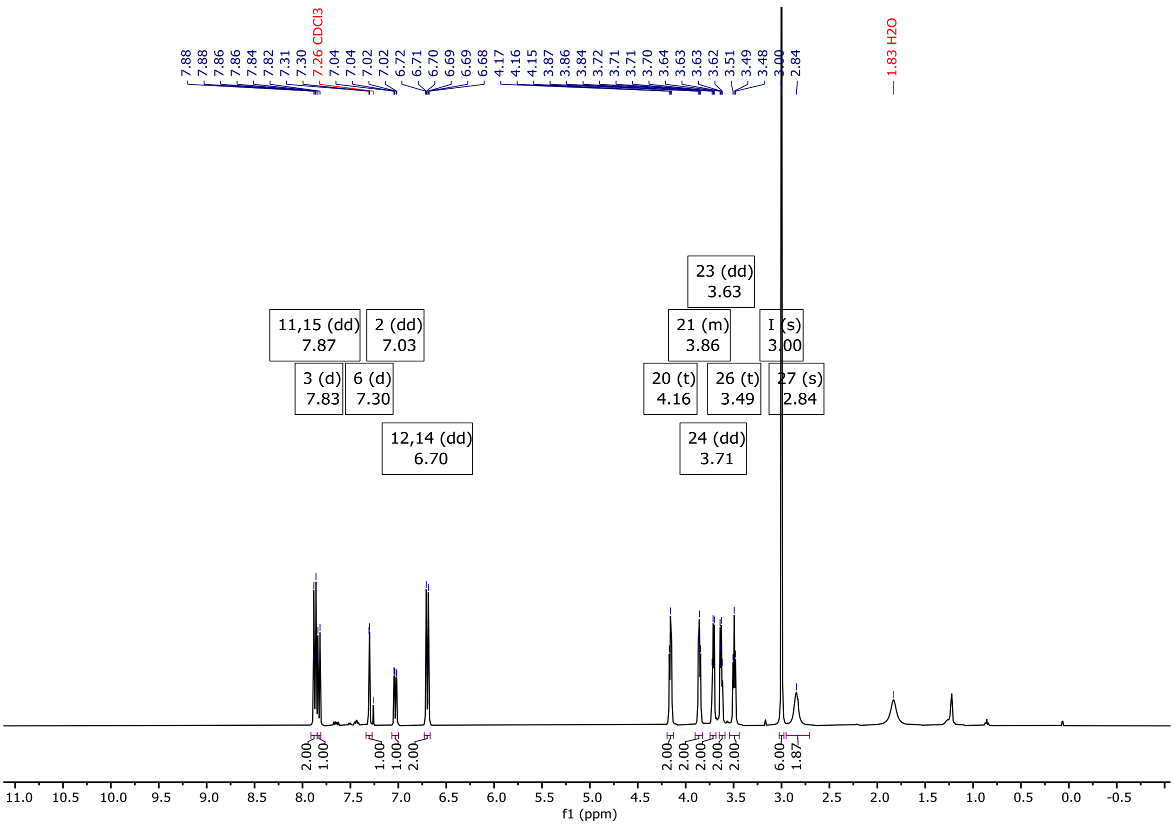

Figure S7. ^{1}H NMR (400 MHz, chloroform-*d*) spectra of **BTA-2-1.**

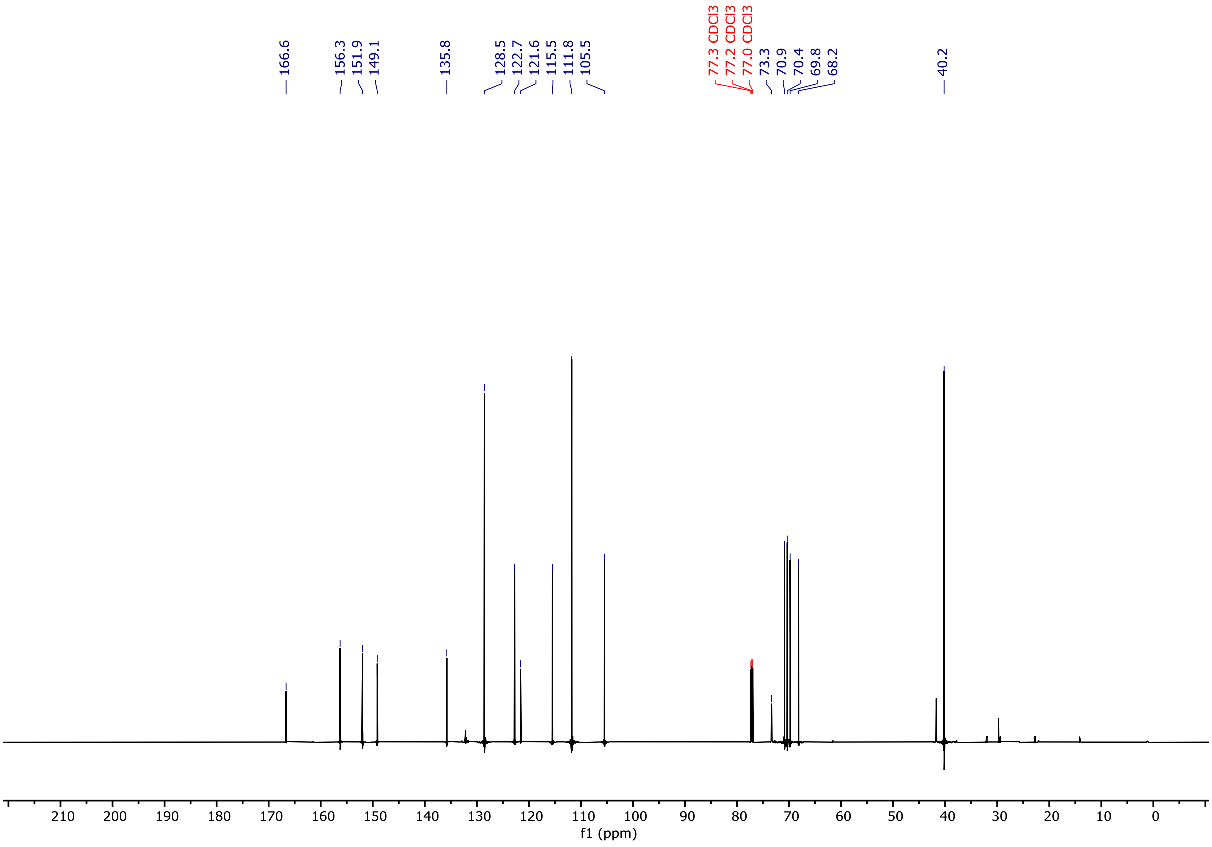

Figure S8. ^{13}C NMR (176 MHz, chloroform-*d*) spectra of **BTA-2-1.**

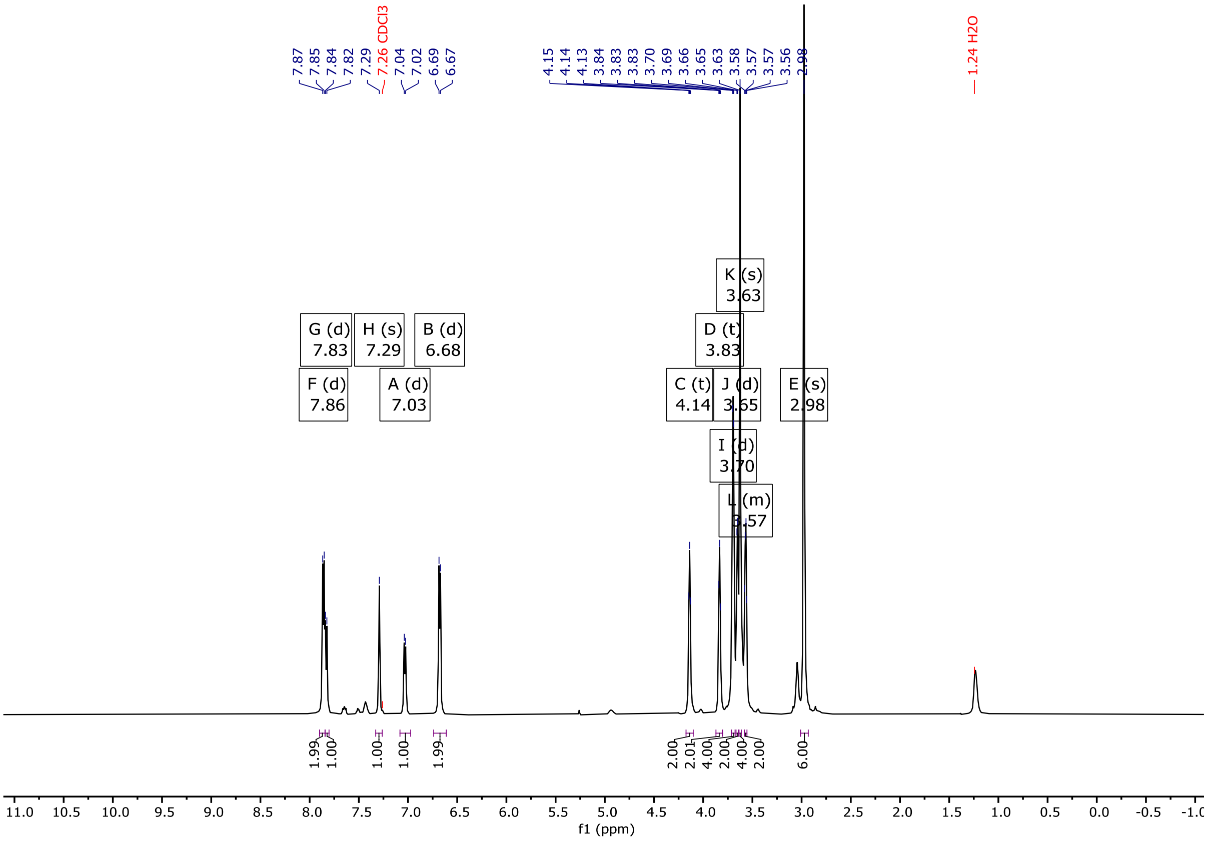

Figure S9. ^{1}H NMR (600 MHz, $CDCl_3$) spectra of **S4**.

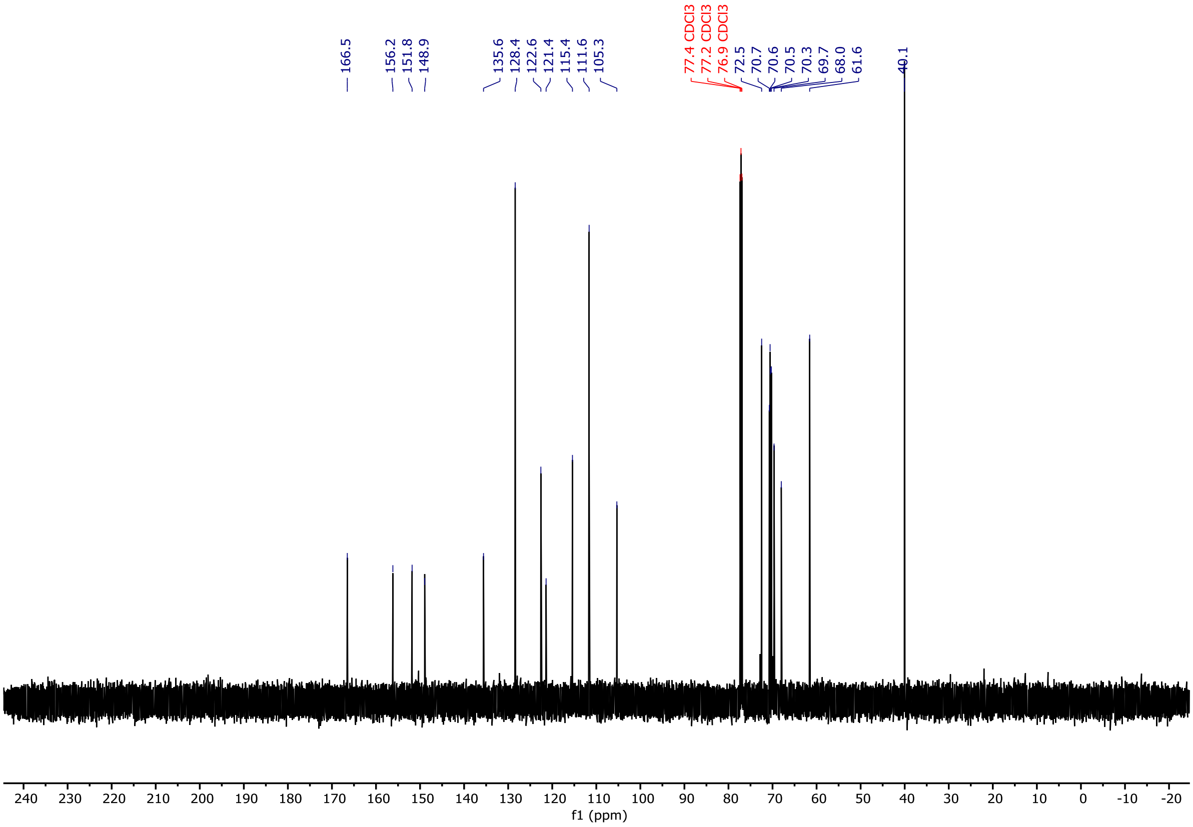

Figure S10. ^{13}C NMR (151 MHz, $CDCl_3$) spectra of **S4**.

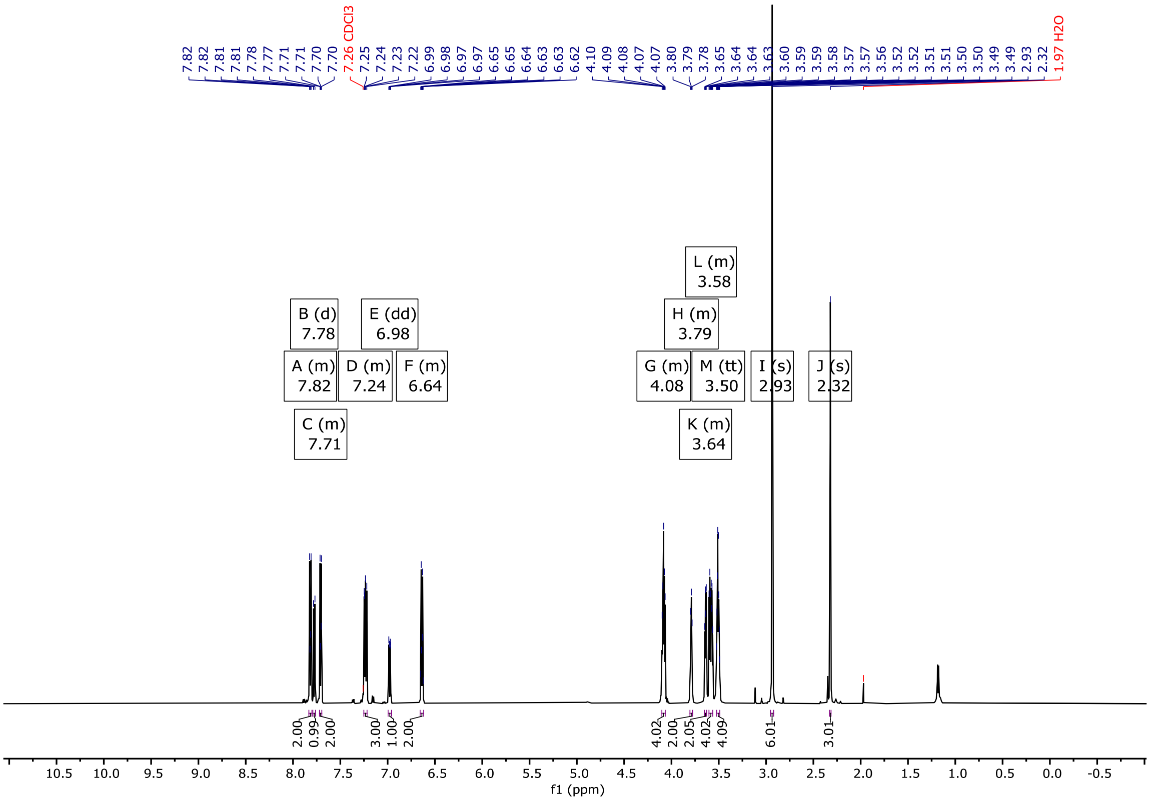

Figure S11. ^{1}H NMR (600 MHz, chloroform-*d*) spectra of **S5.**

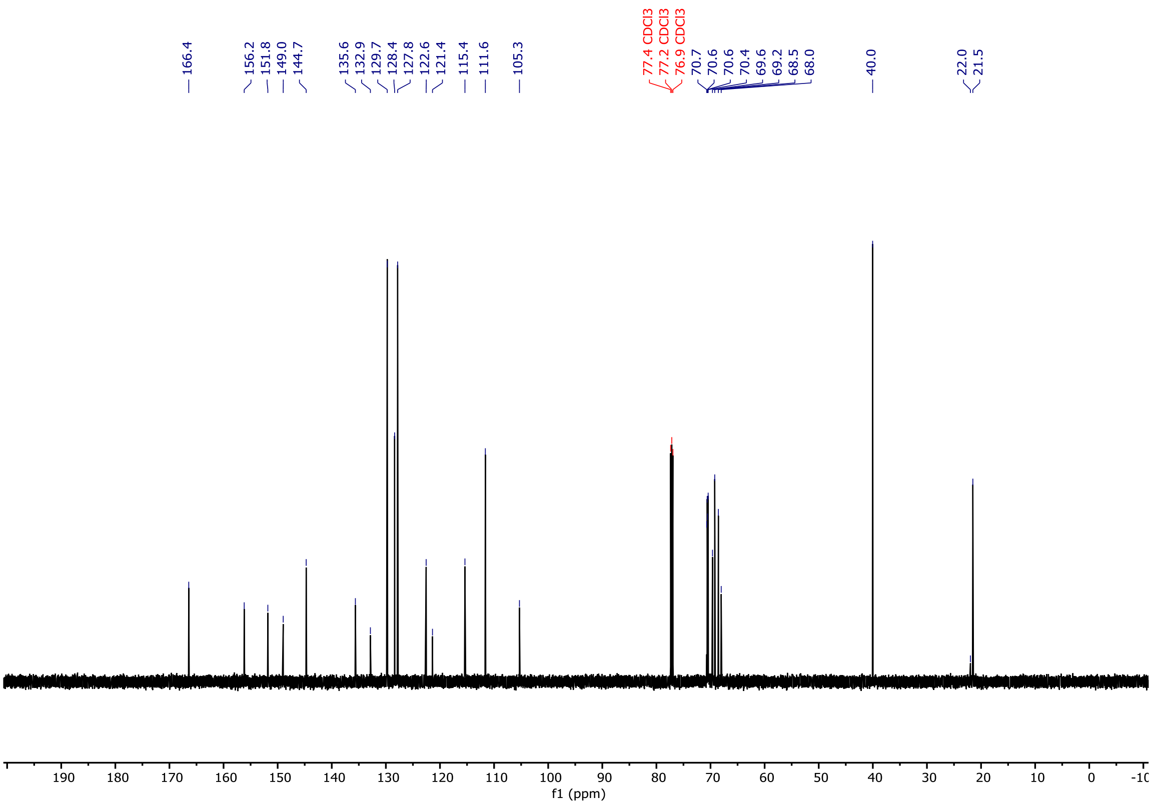

Figure S12. ^{13}C NMR (151 MHz, chloroform-*d*) spectra of **S5.**

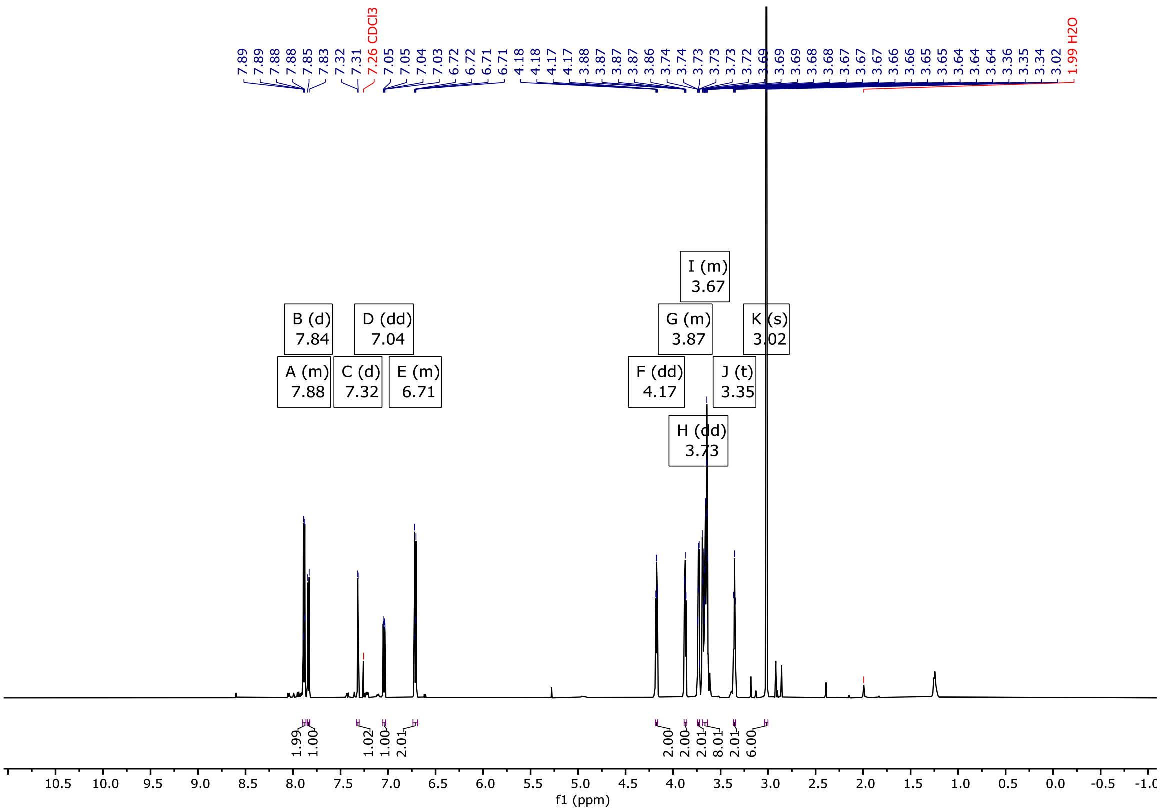

Figure S13. ^{1}H NMR (600 MHz, chloroform-*d*) spectra of **S6**.

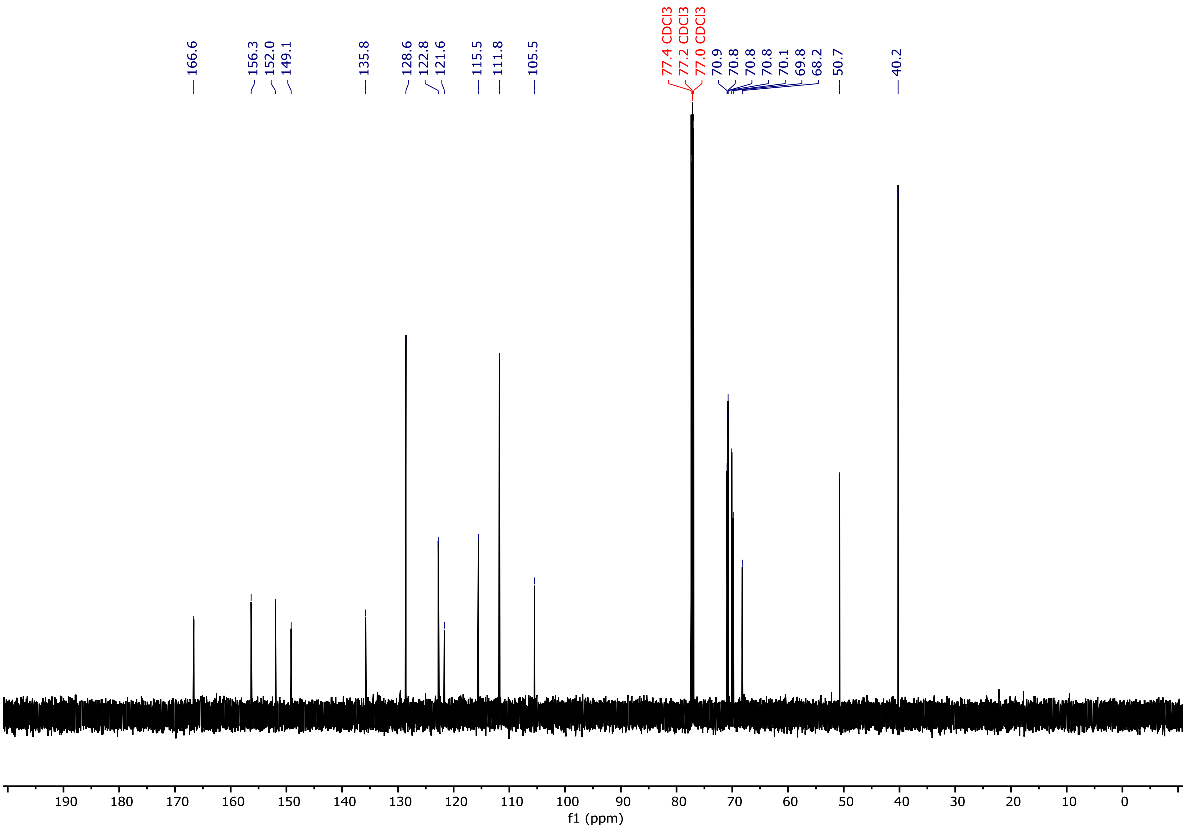

Figure S14. ^{13}C NMR (151 MHz, chloroform-*d*) spectra of **S6**.

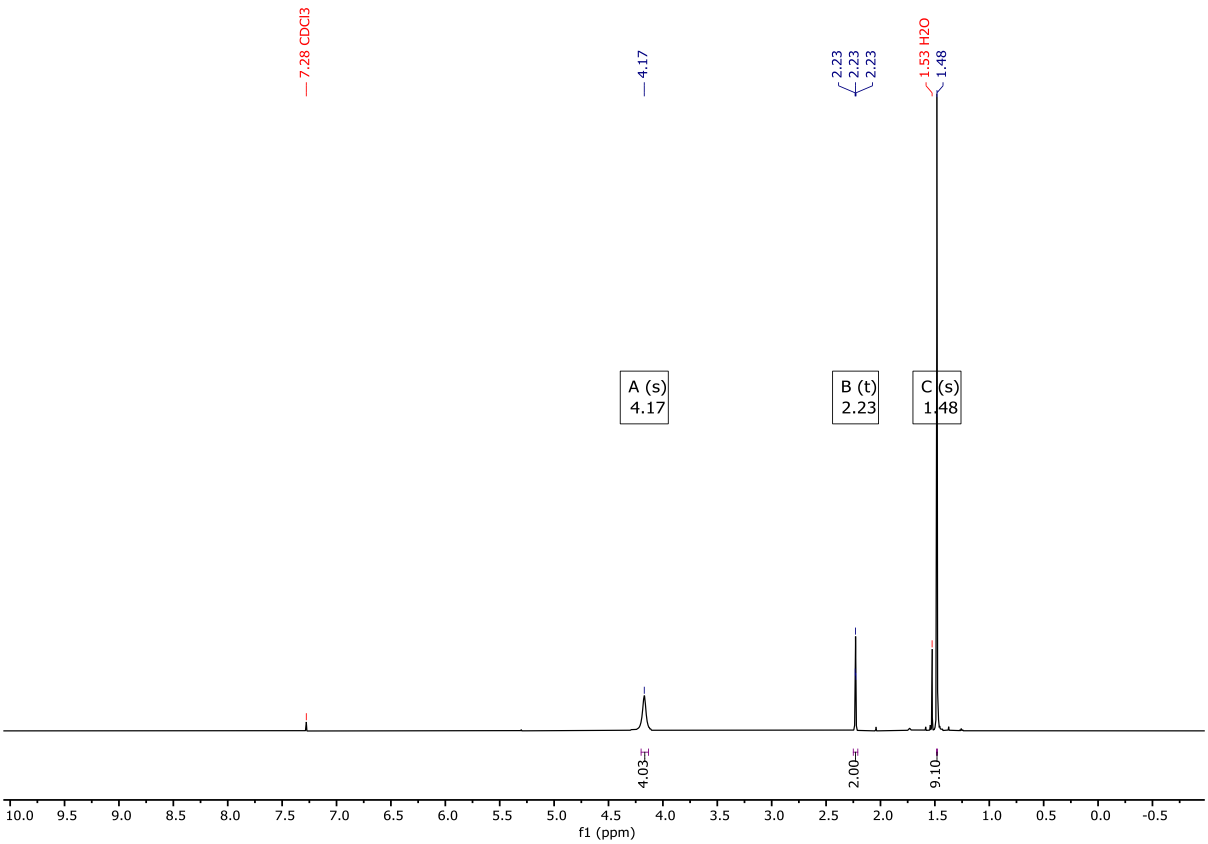

Figure S15. ^{1}H NMR (600 MHz, chloroform-*d*) spectra of **S7**.

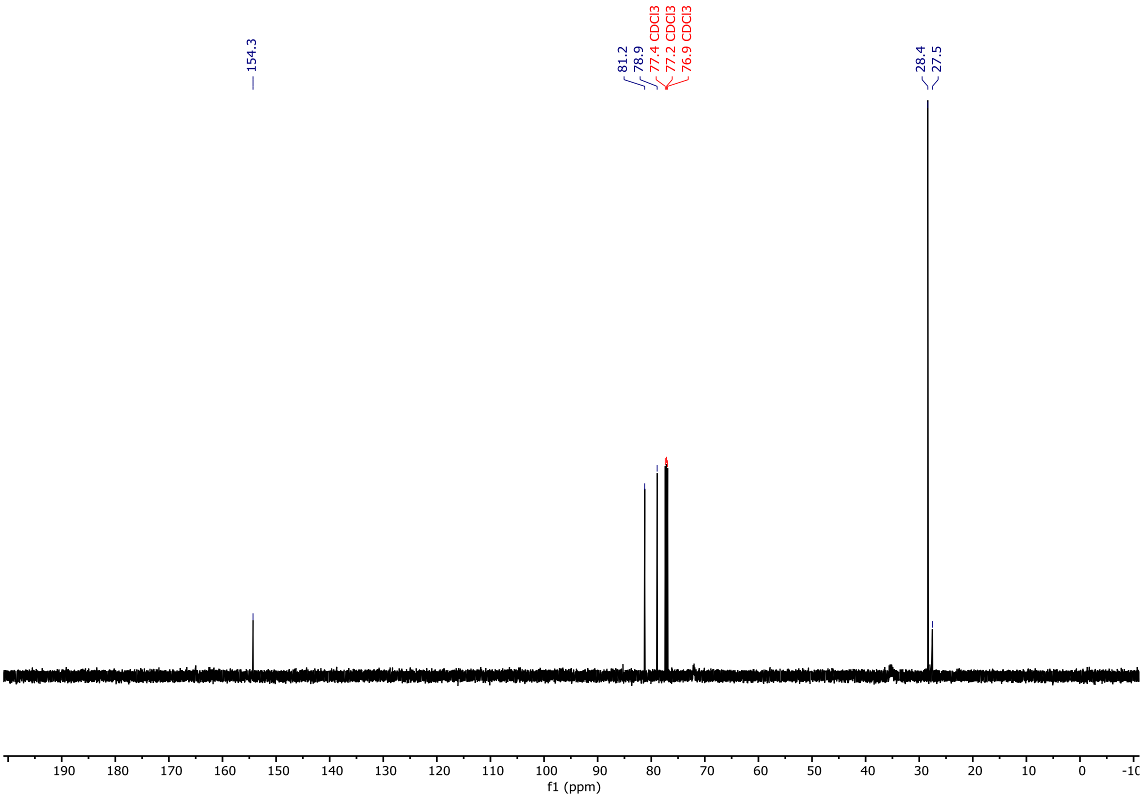

Figure S16. ^{13}C NMR (151 MHz, chloroform-*d*) spectra of **S7.**

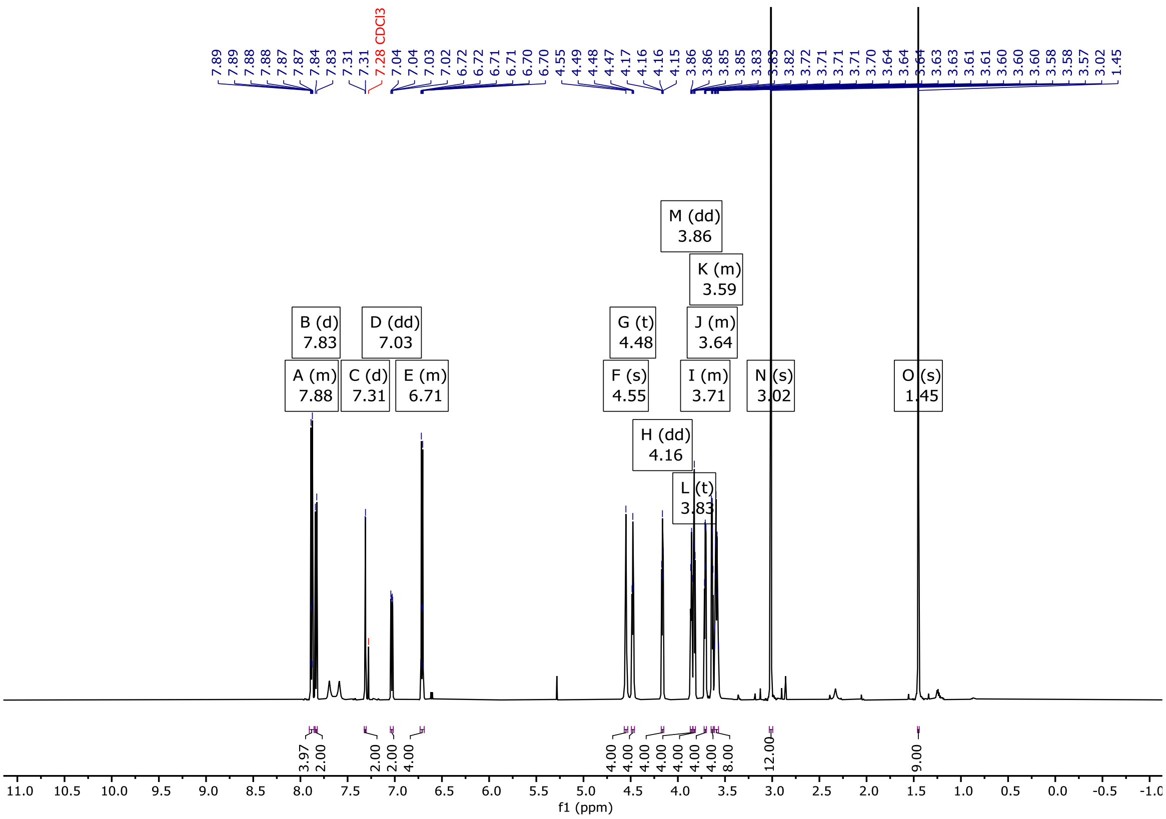

Figure S17. ^{1}H NMR (600 MHz, chloroform-*d*) spectra of **S8.**

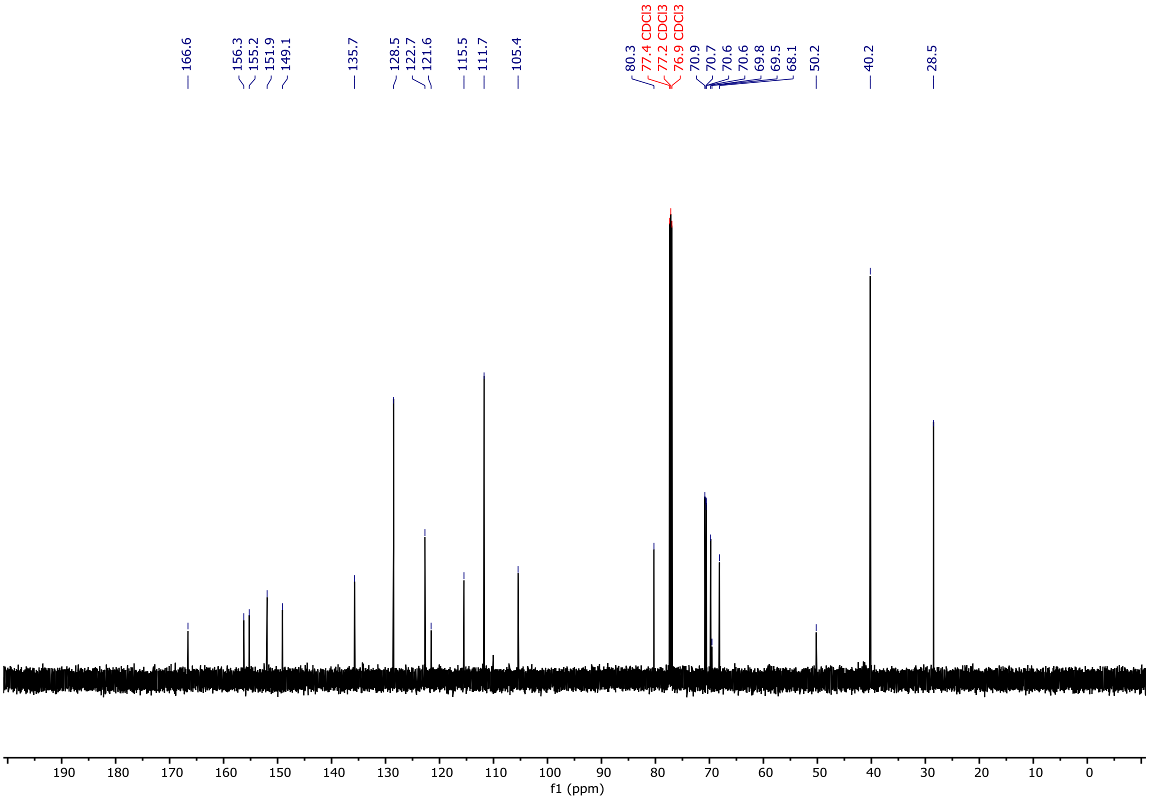

Figure S18. ^{13}C NMR (151 MHz, chloroform-*d*) spectra of **S8.**

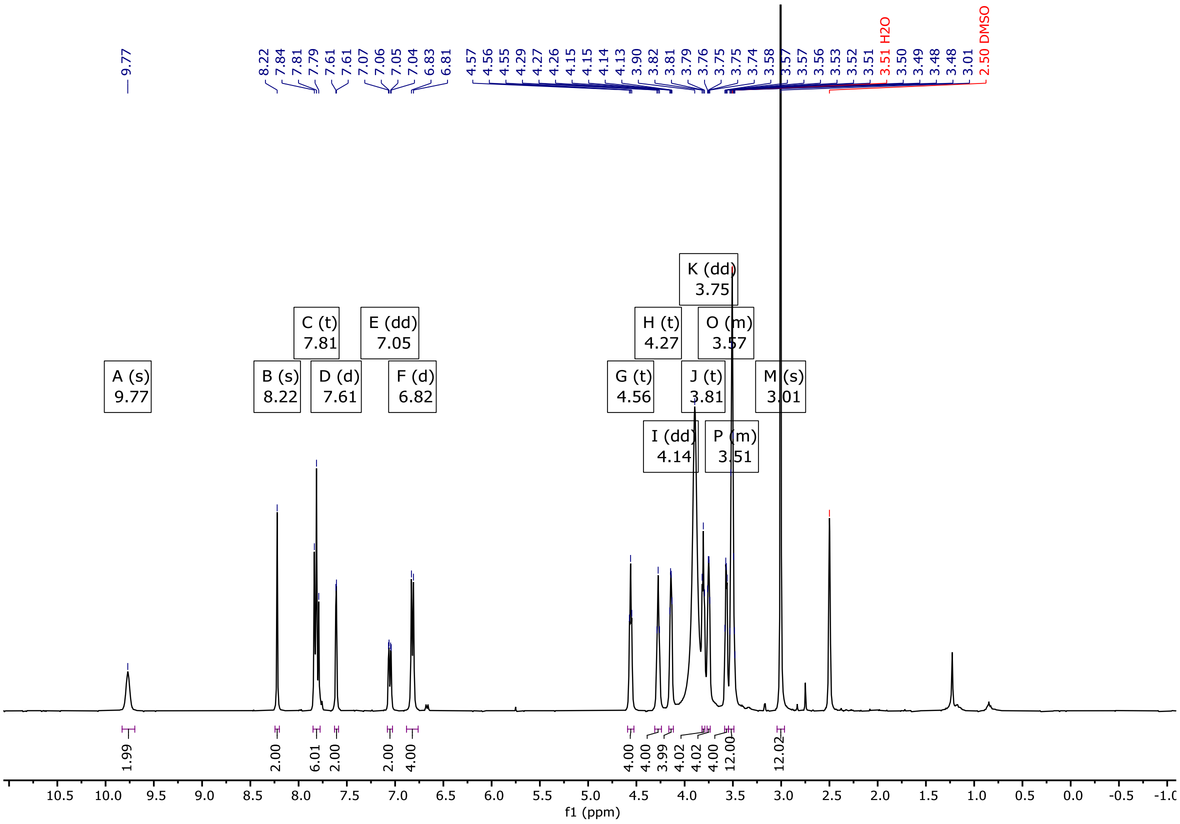

Figure S19. ^{1}H NMR (400 MHz, DMSO-d_6) spectra of **S9.**

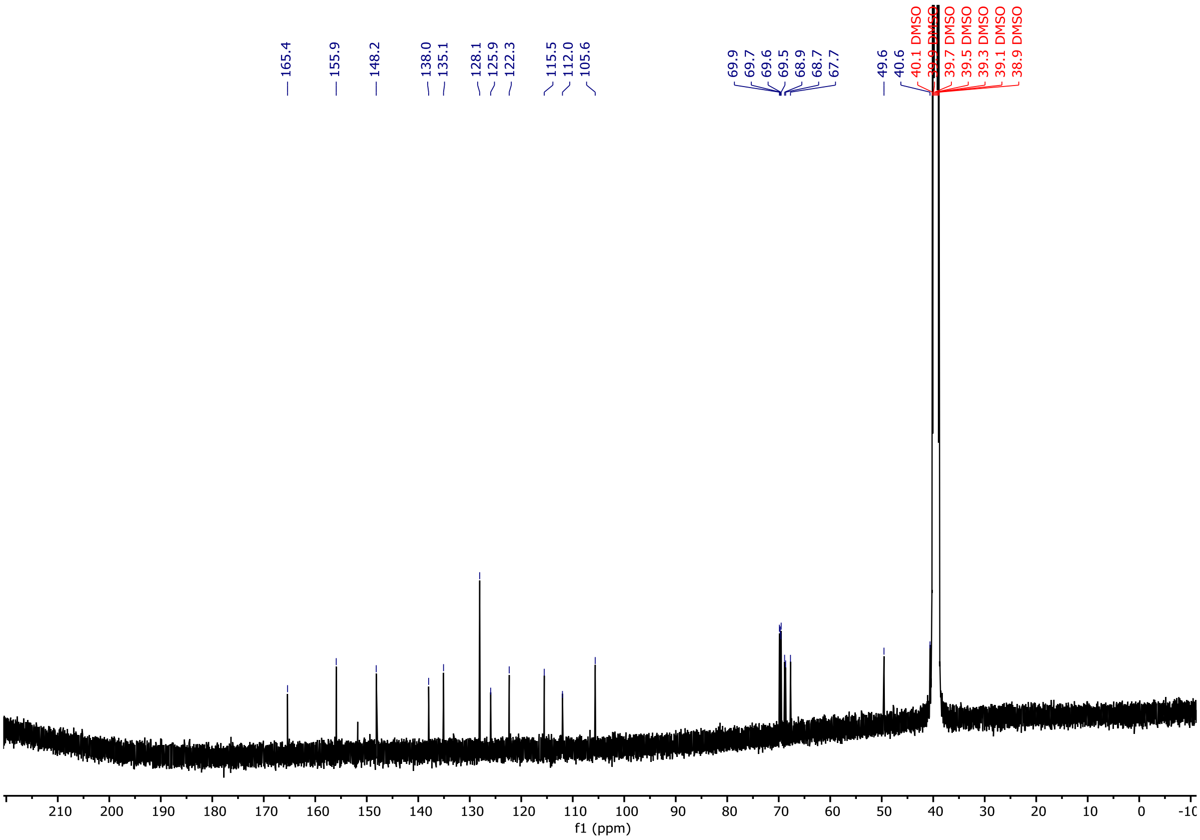

Figure S20. ^{13}C NMR (101 MHz, DMSO-d_6) spectra of **S9.**

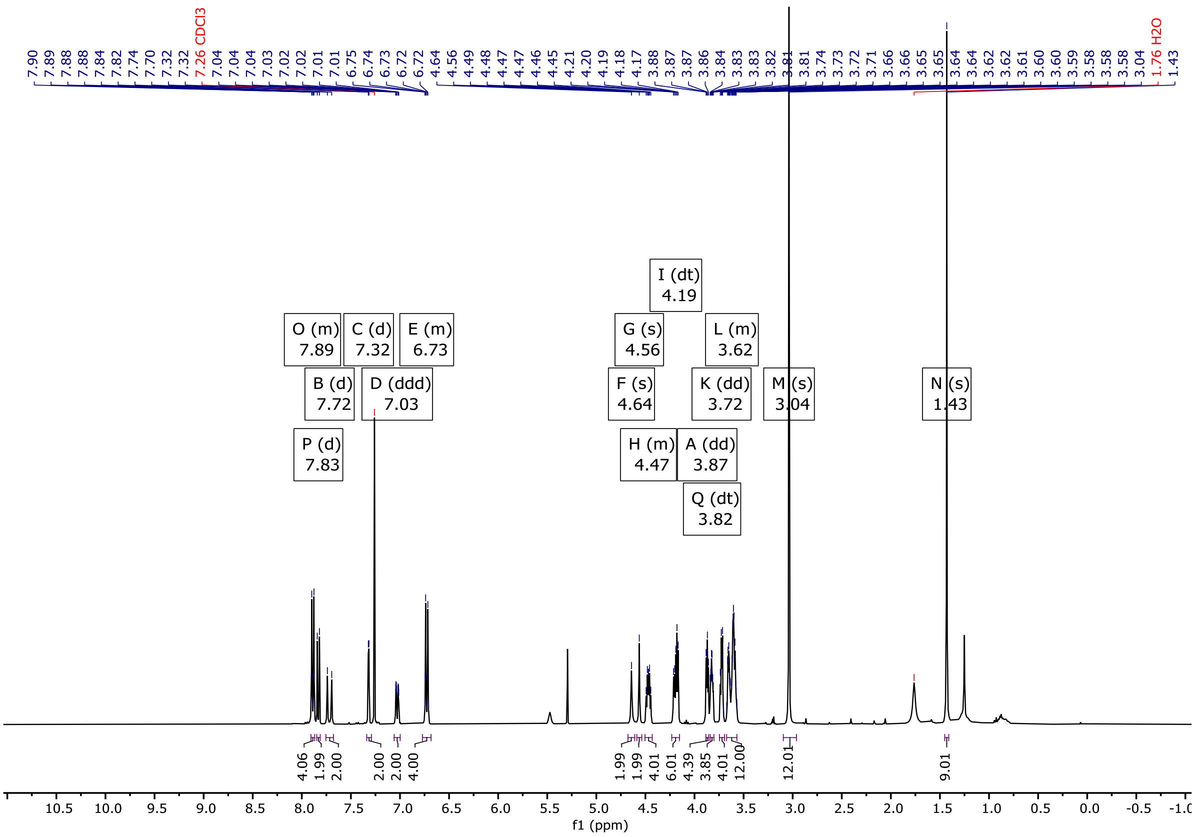

Figure S21. ^{1}H NMR (400 MHz, chloroform-*d*) spectra of **S10**.

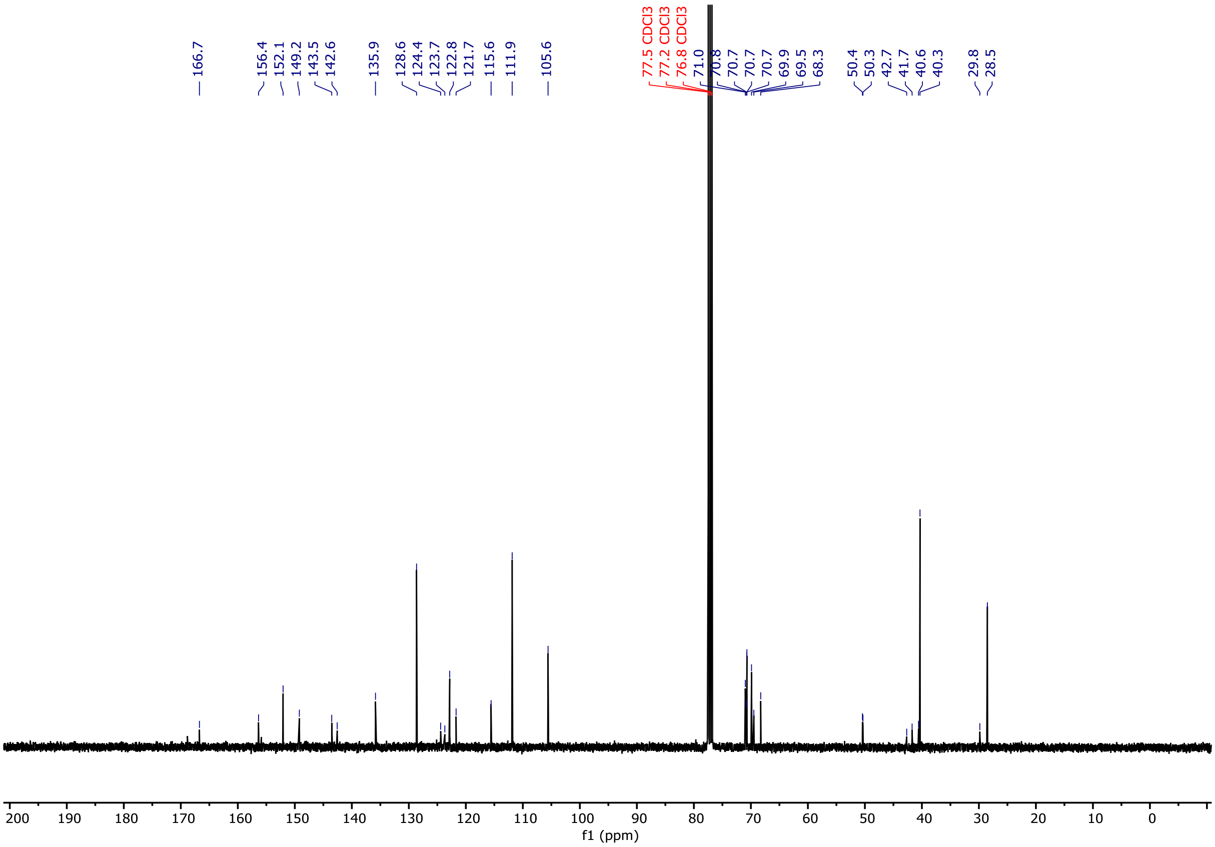

Figure S22. ^{13}C NMR (101 MHz, chloroform-*d*) spectra of **S10**.

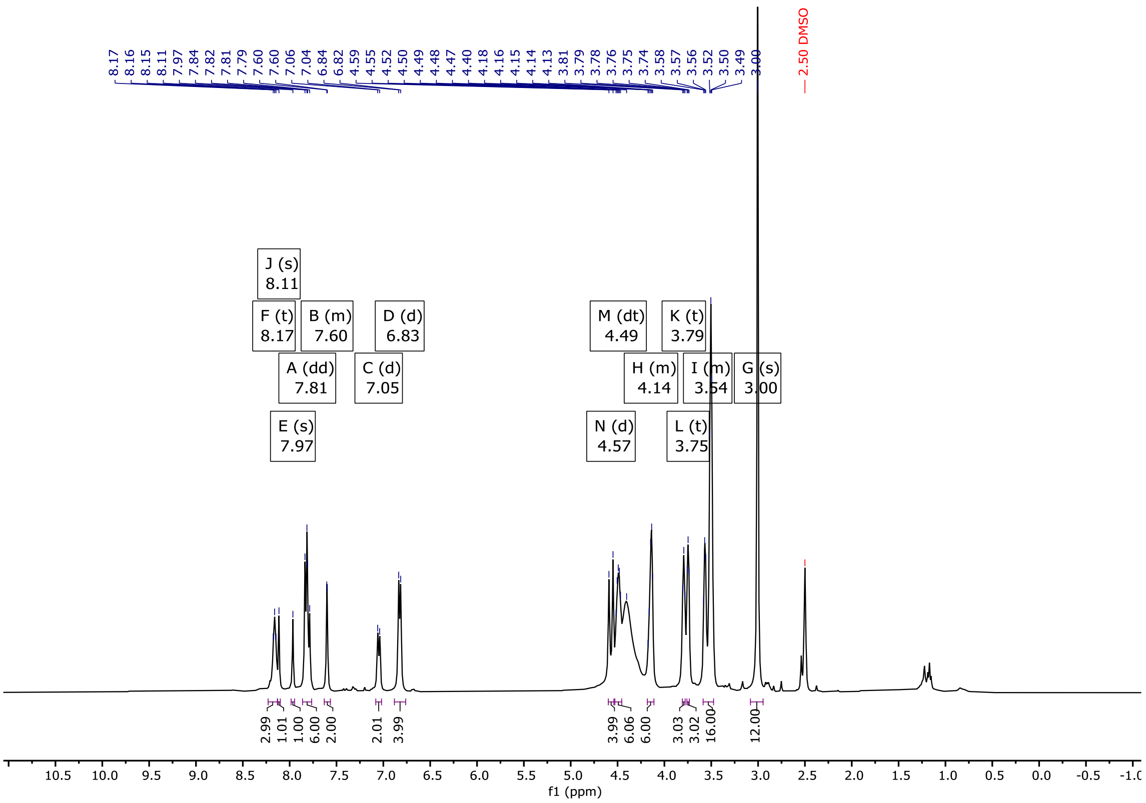

Figure S23. ^{1}H NMR (400 MHz, DMSO-d_6) spectra of **BTA-2-2.**

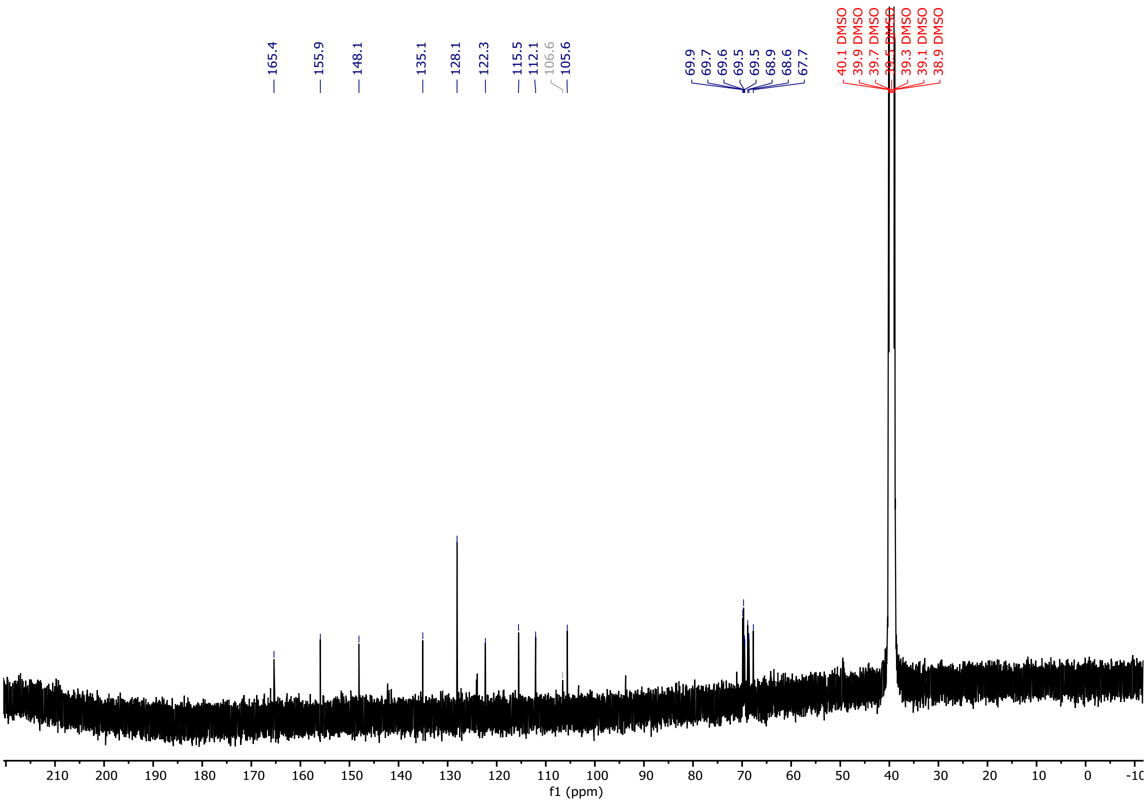

Figure S24. ^{13}C NMR (101 MHz, DMSO-d_6) spectra of **BTA-2-2.**

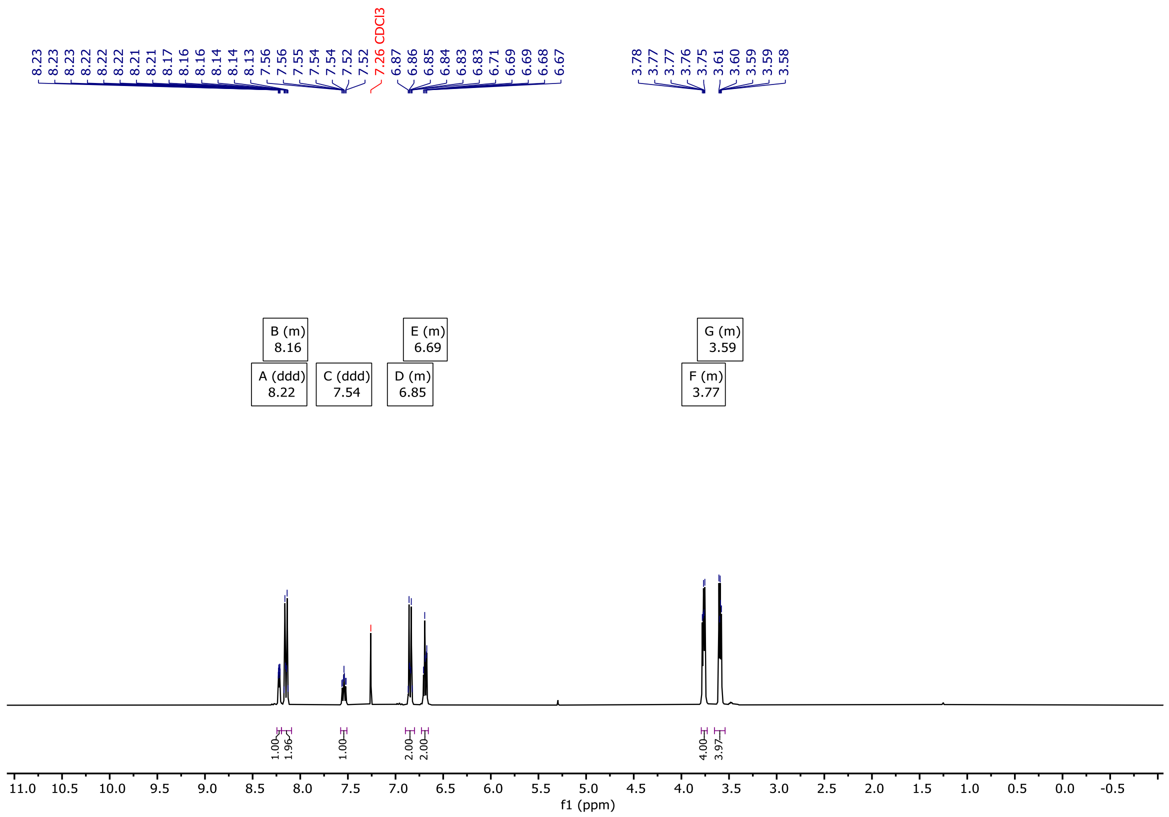

Figure S25. ^{1}H NMR (400 MHz, $CDCl_3$) spectra of **S11.**

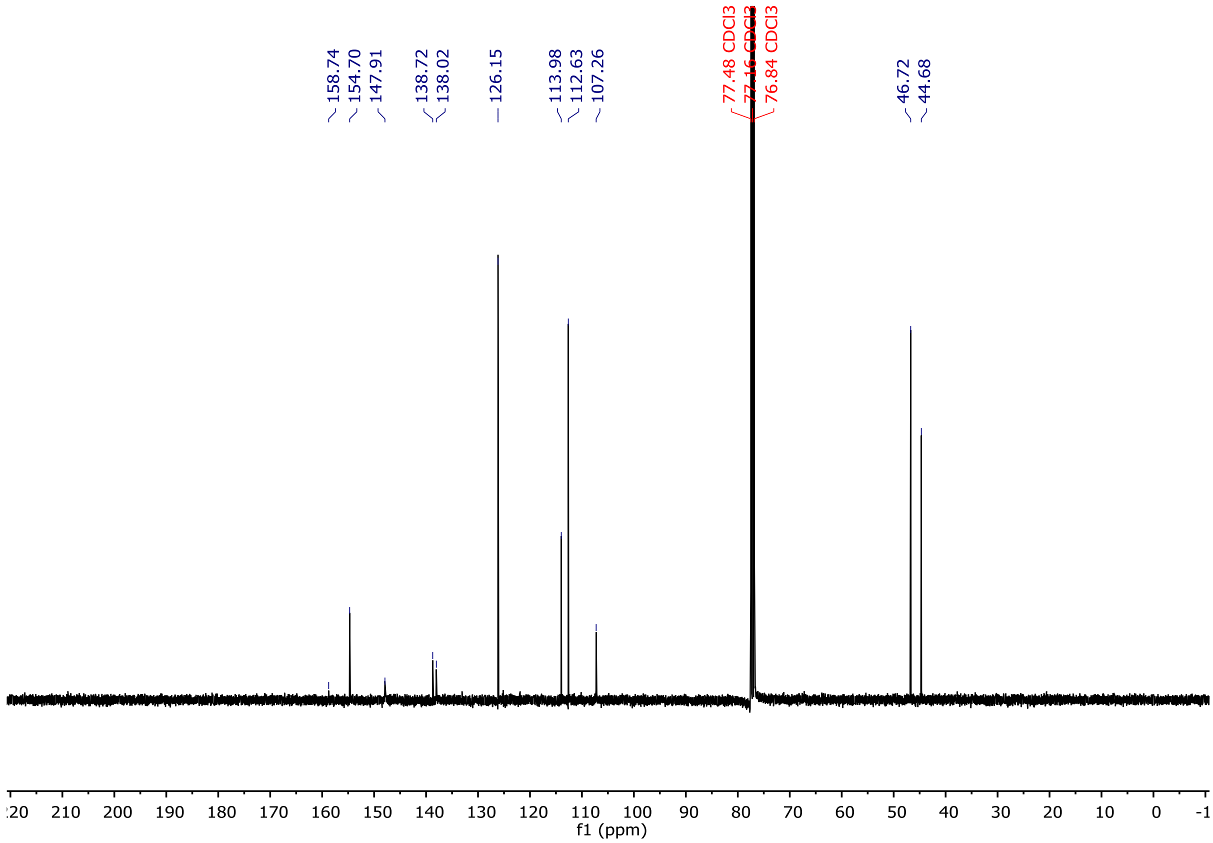

Figure S26. ^{13}C NMR (101 MHz, $CDCl_3$) spectra of **S11.**

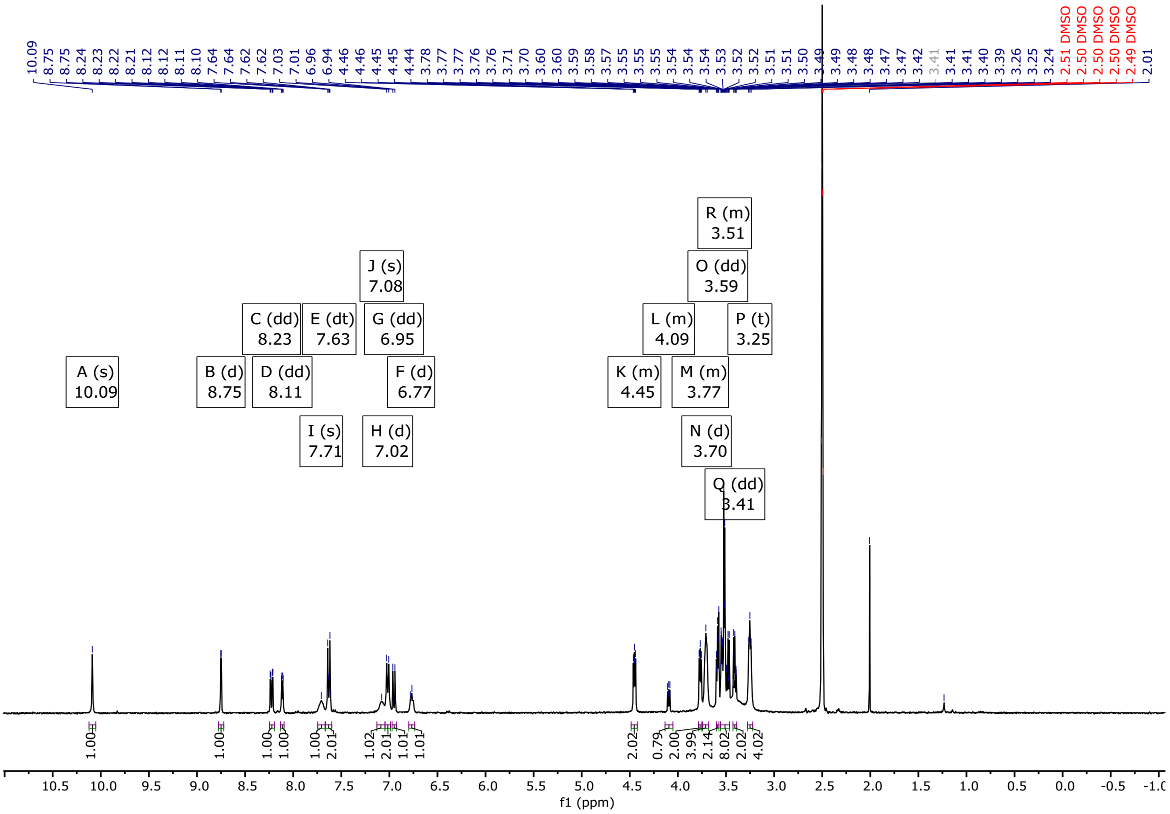

Figure S27. ^{1}H NMR (400 MHz, DMSO-d_6) spectra of **S10.**

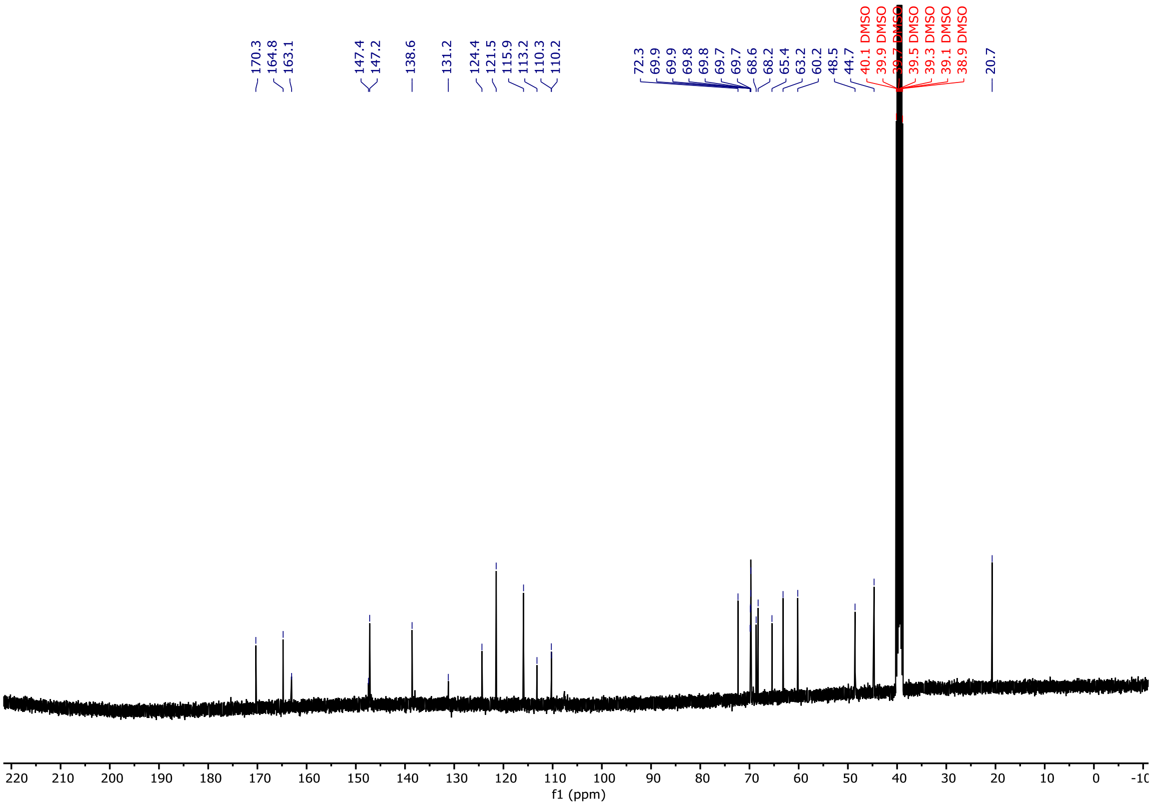

Figure S28. ^{13}C NMR (101 MHz, DMSO-d_6) spectra of **S10.**

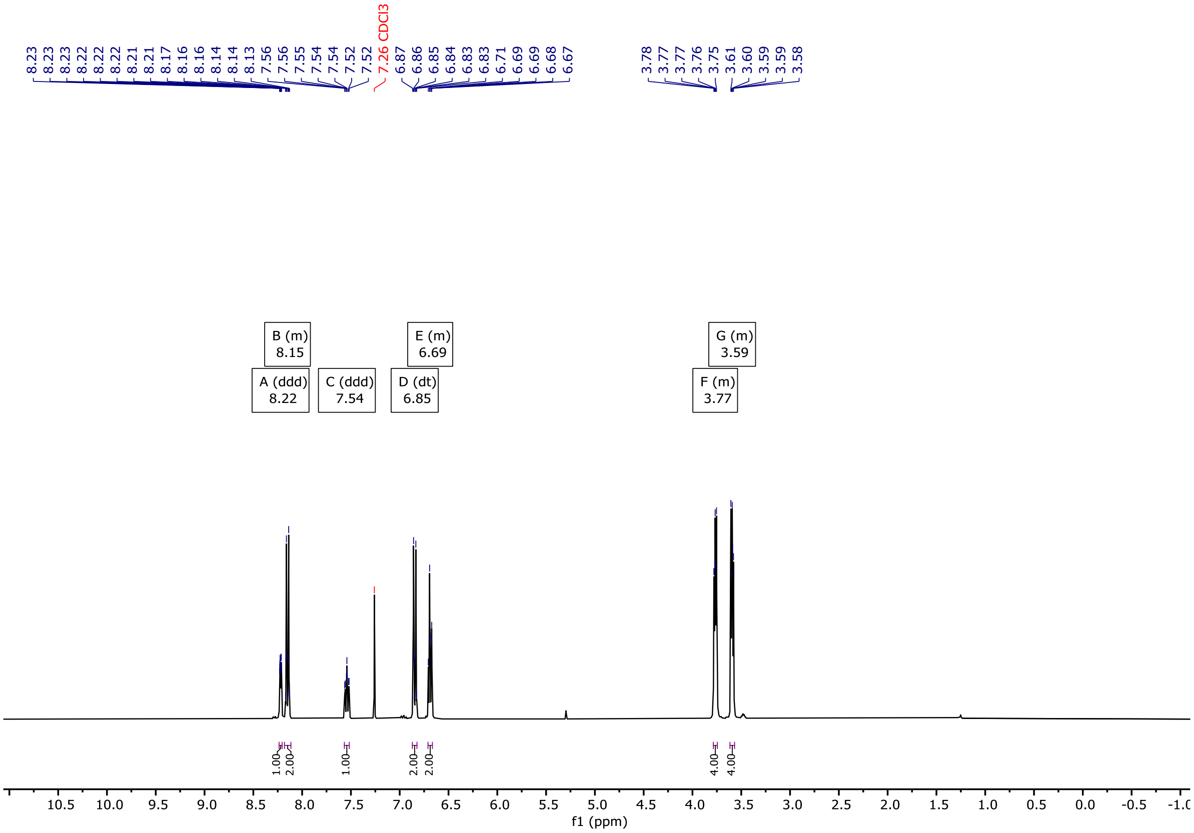

Figure S29. ^{1}H NMR (400 MHz, $CDCl_3$) spectra of **S11.**

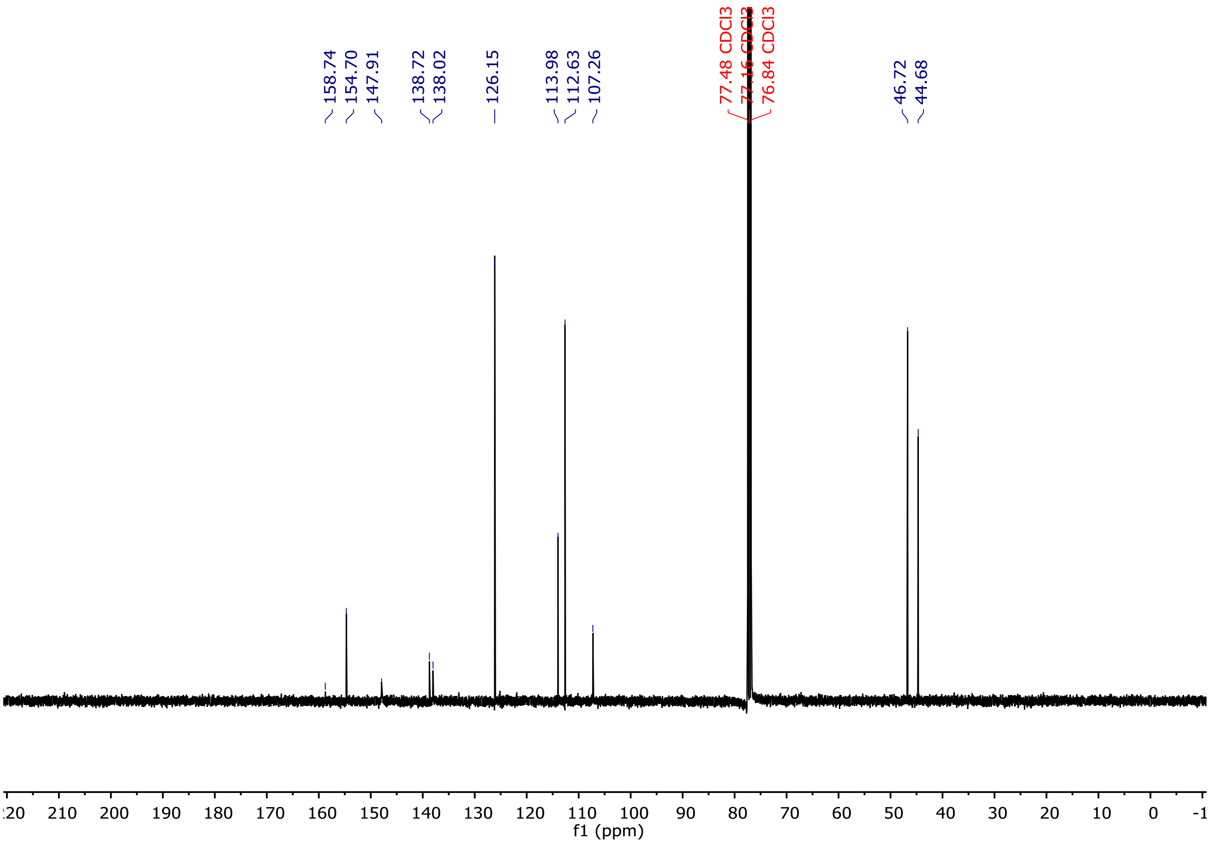

Figure S30. ^{13}C NMR (101 MHz, $CDCl_3$) spectra of **S11.**

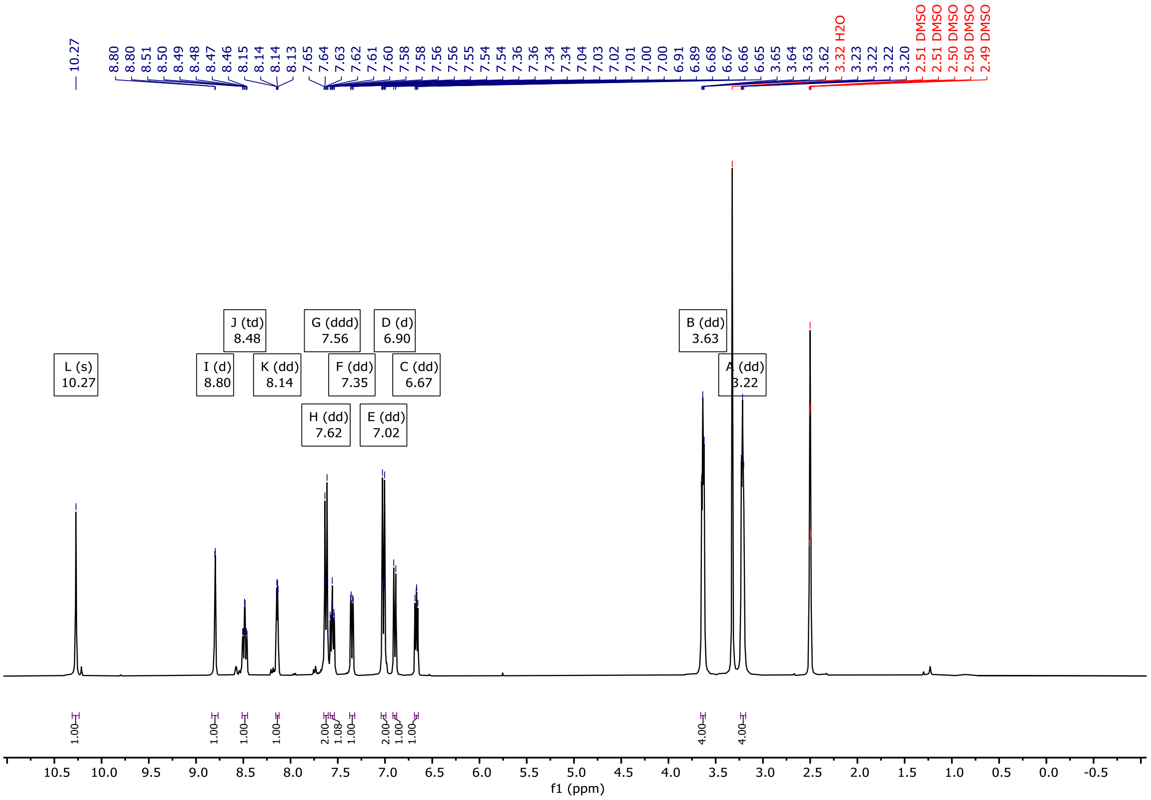

Figure S31. ^{1}H NMR (400 MHz, DMSO-d_6) spectra of **S12.**

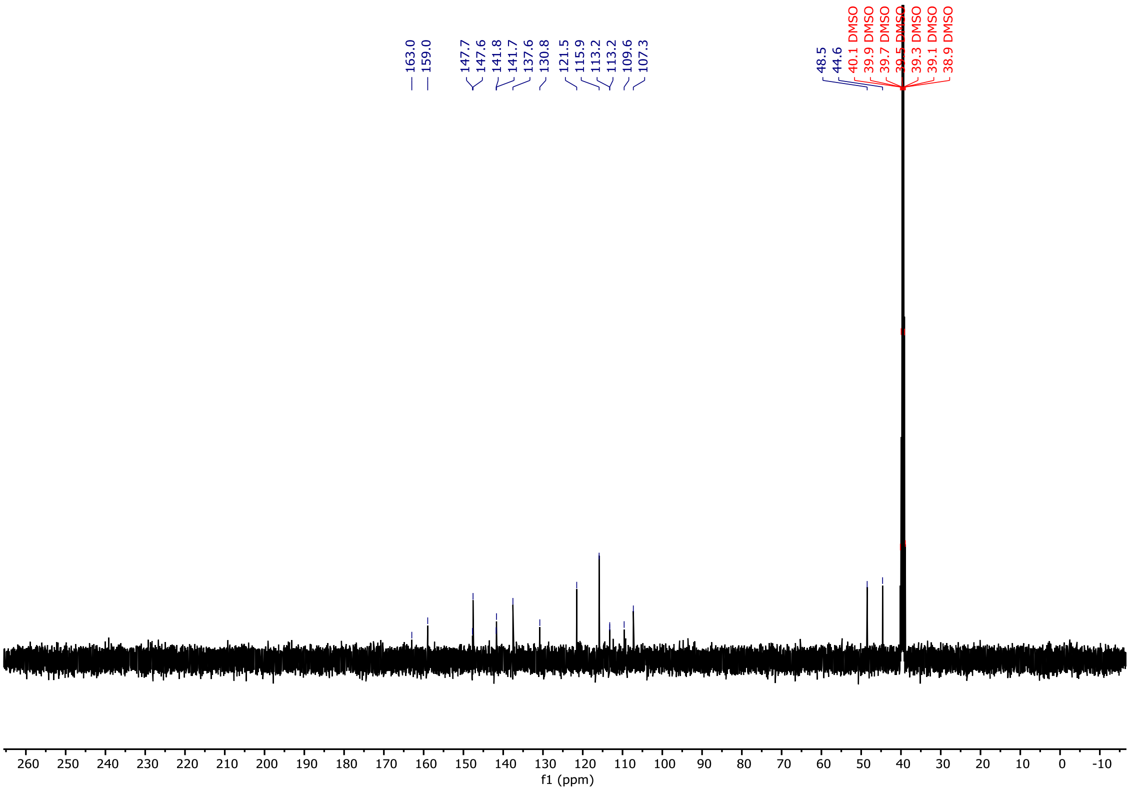

Figure S32. ^{13}C NMR (101 MHz, DMSO-d_6) spectra of **S12**.

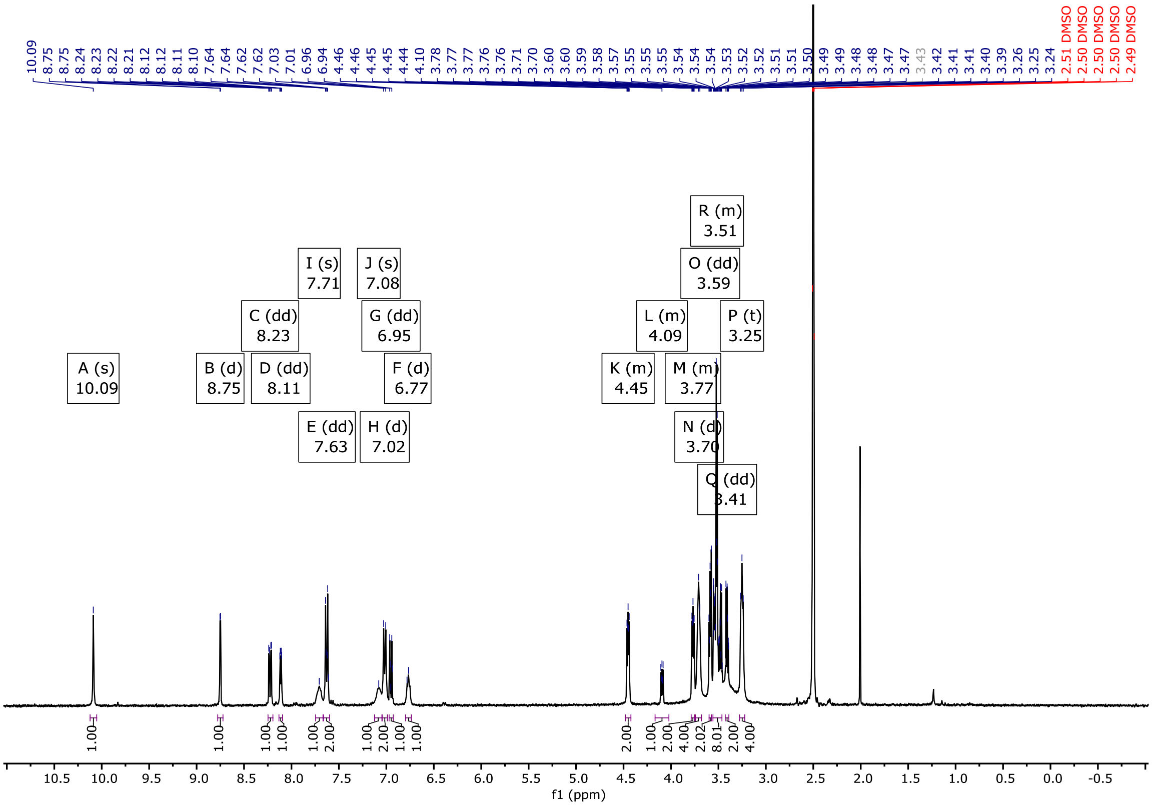

Figure S33. ^{1}H NMR (400 MHz, DMSO-d_6) spectra of **S13.**

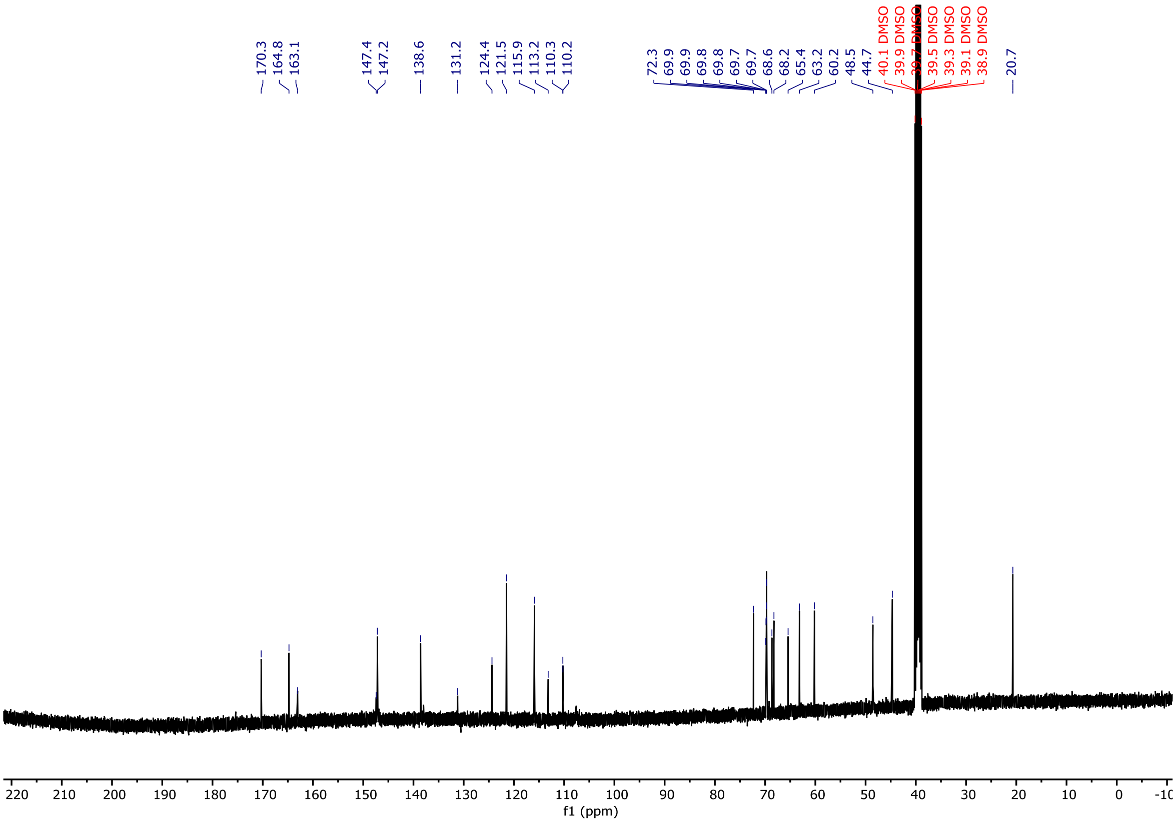

Figure S34. ^{13}C NMR (101 MHz, DMSO-d_6) spectra of **S13**.

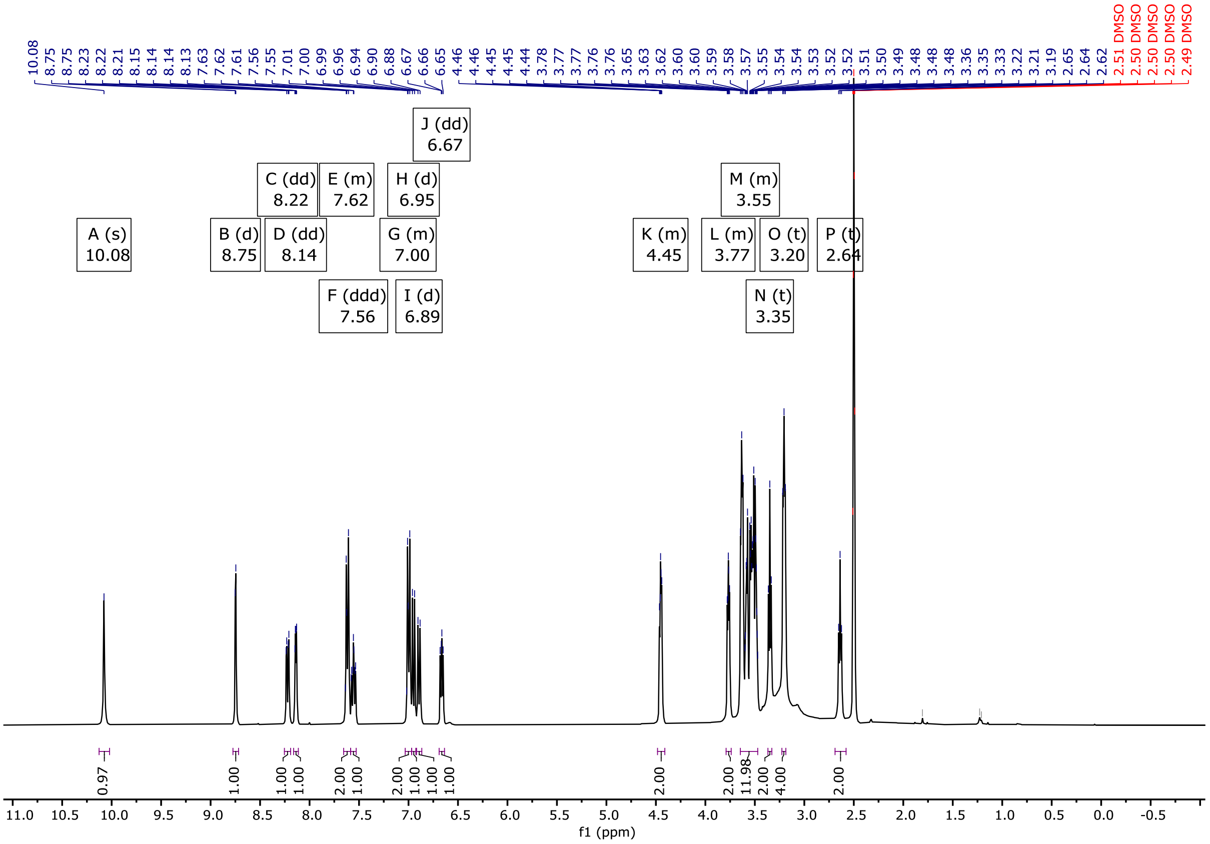

Figure S35. ^{1}H NMR (400 MHz, DMSO-d_6) spectra of **S15.**

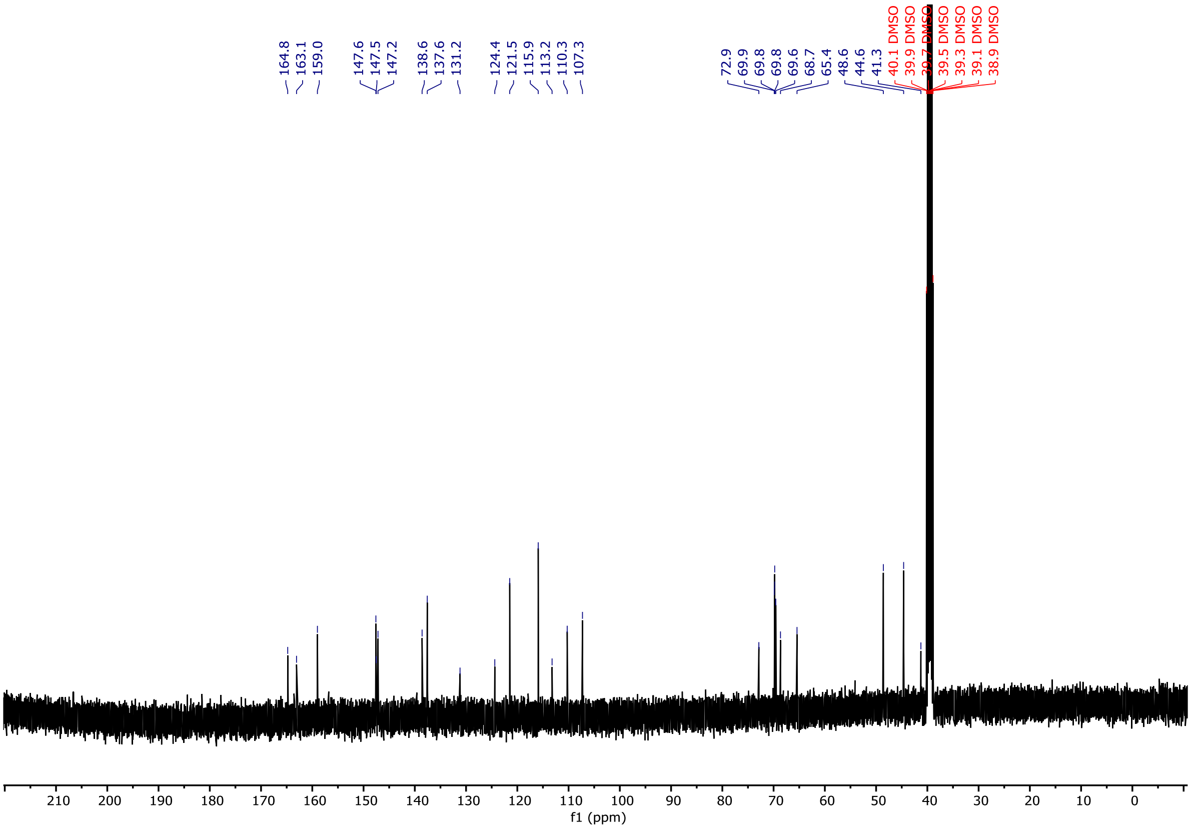

Figure S36. ^{13}C NMR (11 MHz, DMSO-d_6) spectra of **S15.**

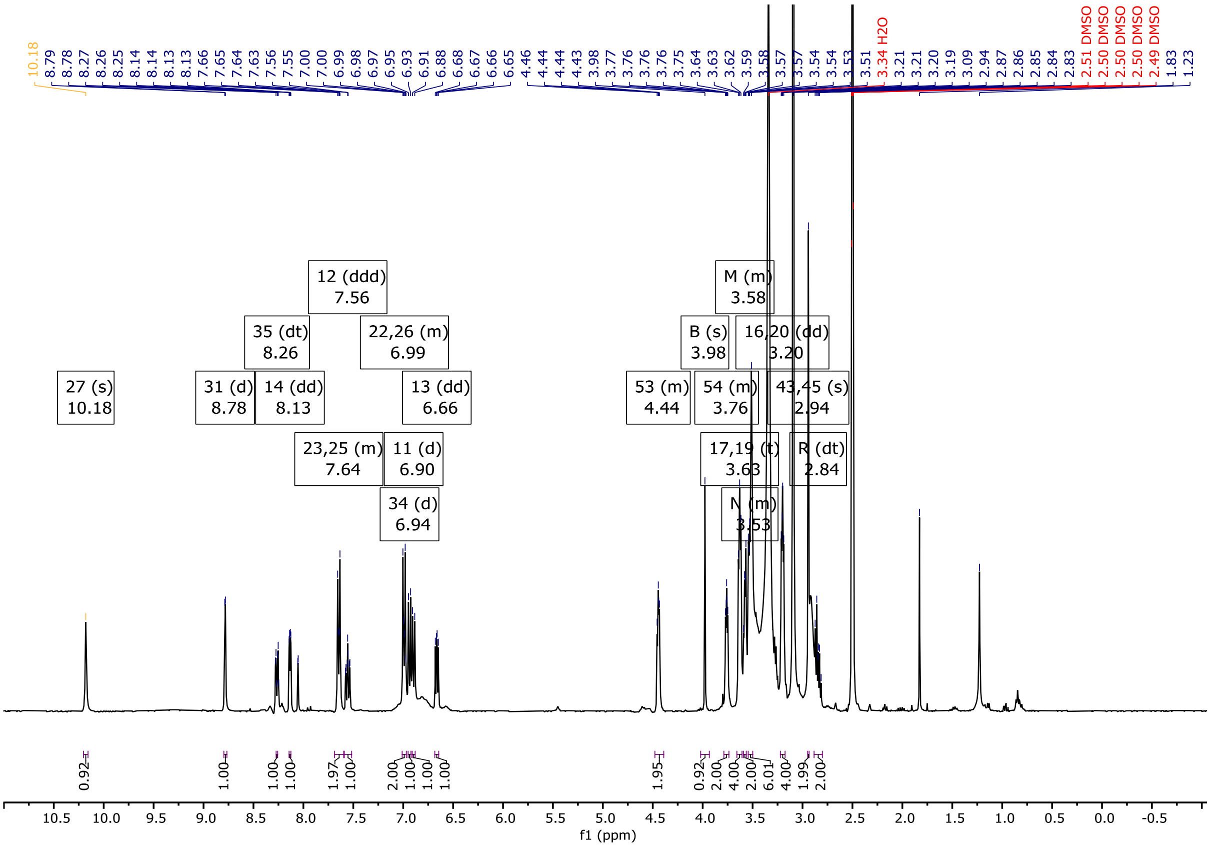

Figure S37. ^{1}H NMR (400 MHz, DMSO-d_6) spectra of **BF-79-1.**

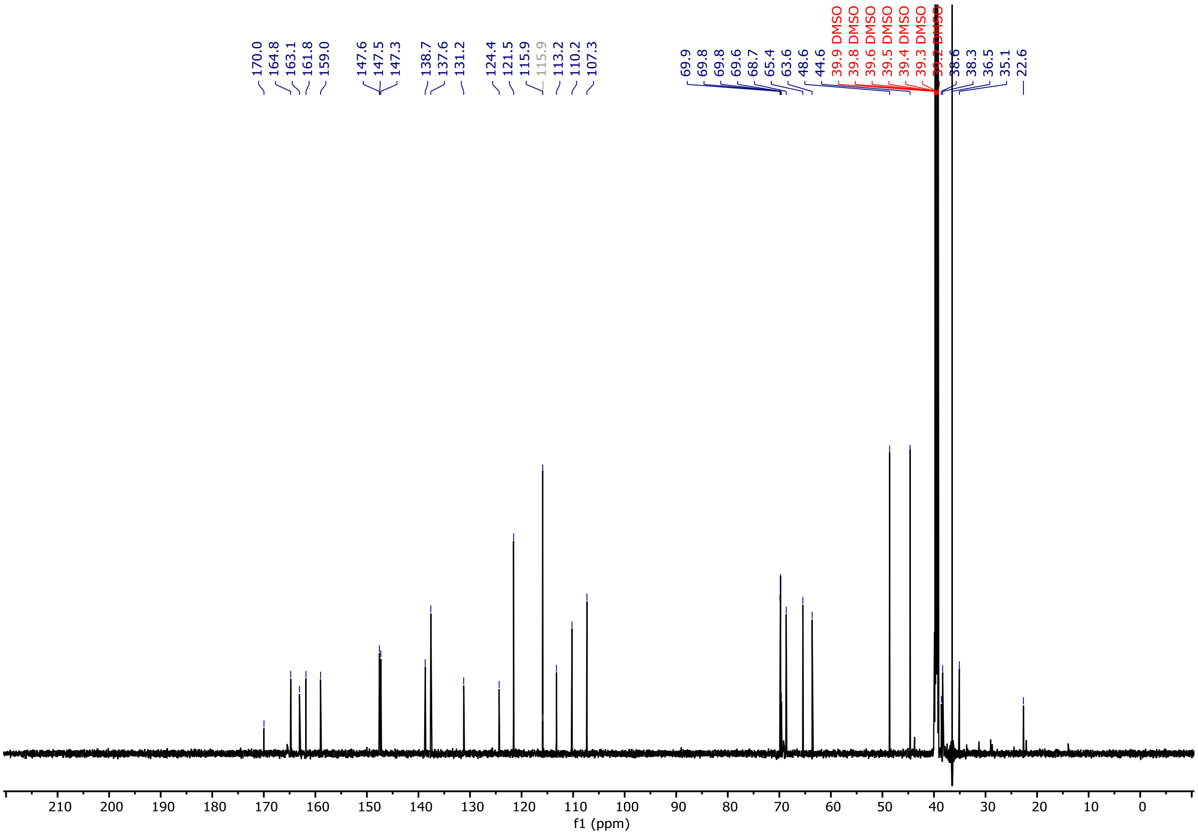

Figure S38. ^{1}H NMR (176 MHz, DMSO-d_6) spectra of **BF-79-1.**

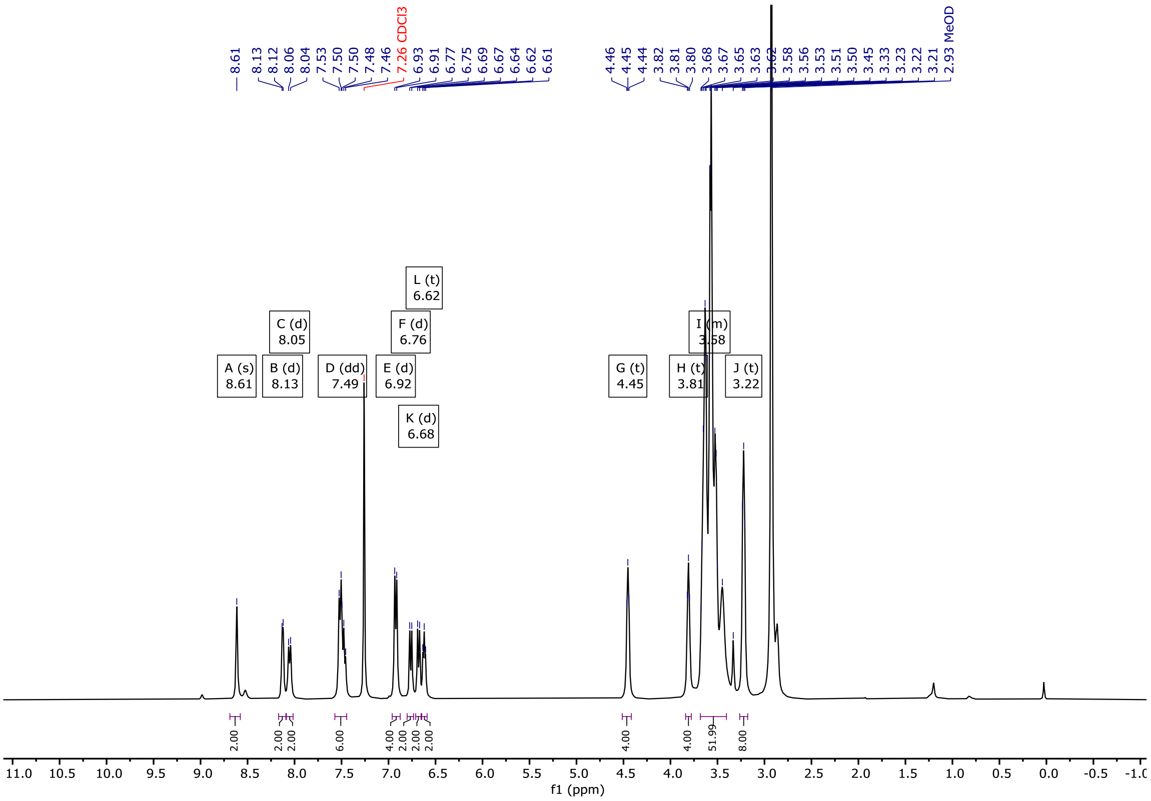

Figure S39. ^{1}H NMR (400 MHz, 9:1 chloroform-*d:*methanol-d_4) spectra of **BF-79-2.**

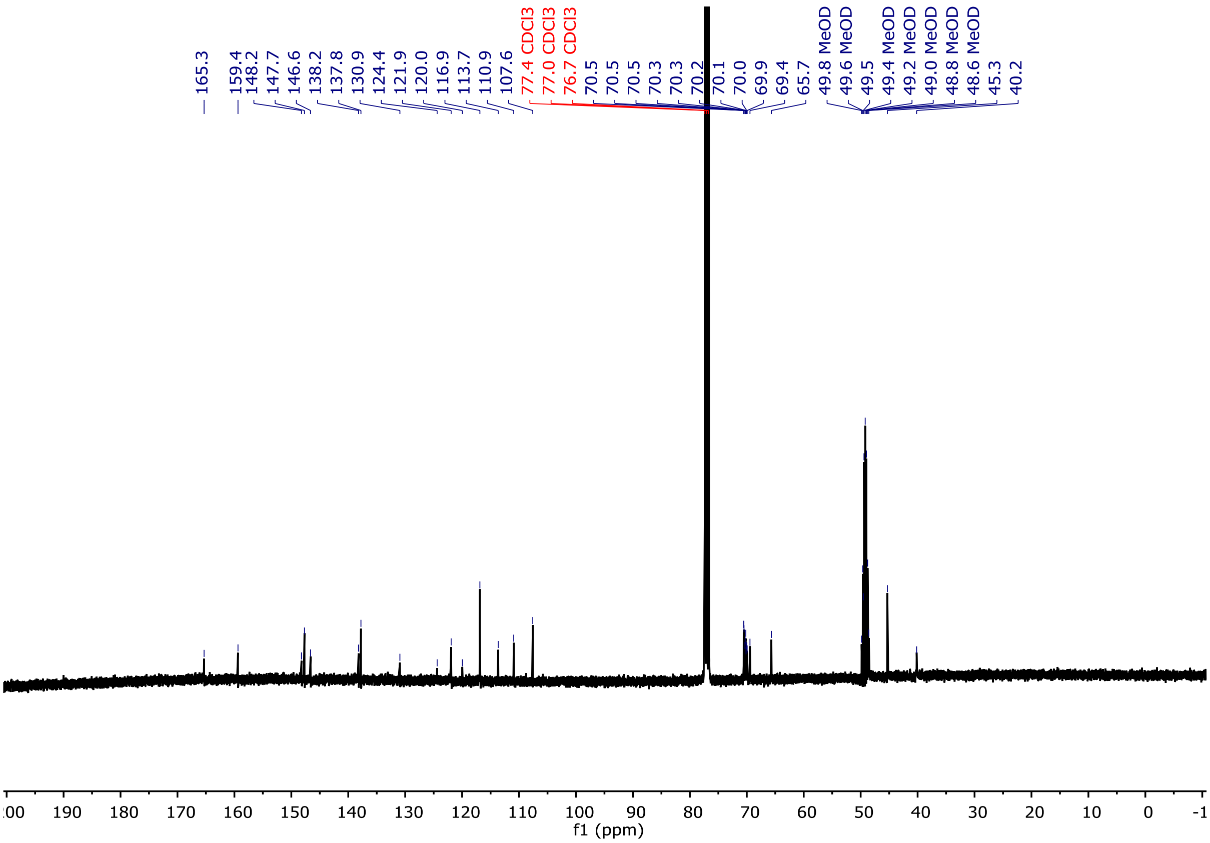

Figure S40. ^{13}C NMR (101 MHz, 9:1 chloroform-*d:* methanol-d_4) spectra of **BF-79-2**.

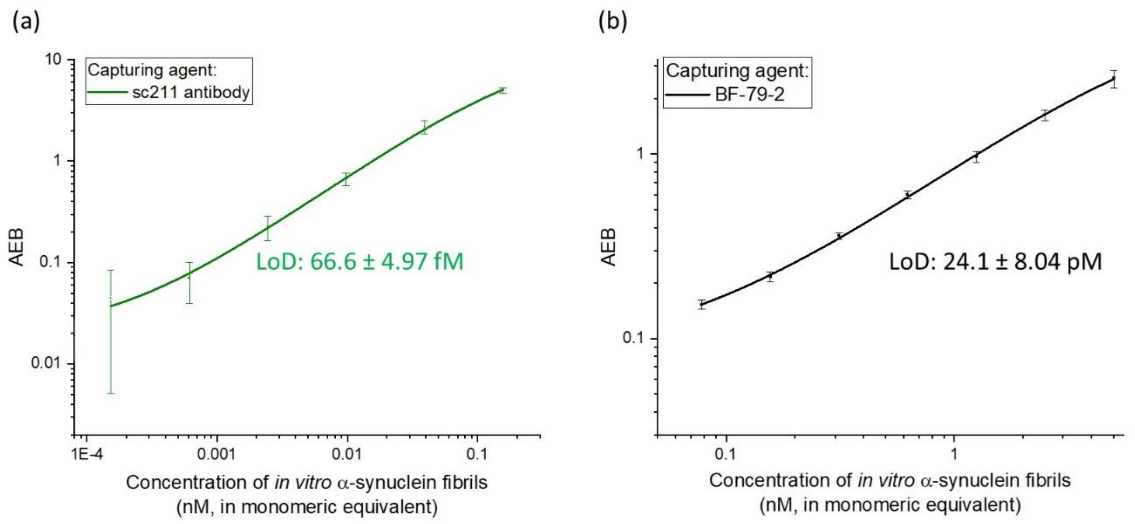

Figure S41. The performance of SiMoA assay with *in vitro* α-synuclein fibrils using (a) sc211 antibody and (b) BF-79-2 as capturing agent. All graphs were fitted by four-parameter logistic (4PL) curve. LoD represents the limit of detection, i.e. the concentration of α-synuclein fibrils (in monomeric equivalent) at 1.3 times background AEB level. n = 6 technical replicates. All data are expressed as mean ± standard error of the mean.

Table S1. Details of commercially available sample diluents.

Name	Abbreviation	Components and details
Sample diluent A	A	Phosphate buffer (pH 7.35-7.45) with bovine serum components, a heterophilic blocker, and a surfactant
Sample diluent B	B	Phosphate buffer (pH 7.35-7.45) with bovine serum components, a heterophilic blocker, and a high surfactant concentration
Sample diluent C	C	Phosphate buffer (pH 7.35-7.45) with low concentration of bovine serum components, a heterophilic blocker, and a surfactant
Sample diluent D	D	Phosphate buffer (pH 7.35-7.45) with newborn calf serum, a heterophilic blocker, and a surfactant
Sample diluent E	E	Tris buffer with high pH (pH 8.95-9.05), bovine serum components, a heterophilic blocker, and a surfactant
Homebrew sample diluent	Standard	Phosphate buffer (pH 7.35-7.45) with bovine serum components, and a surfactant
